# Quality appraisal for systematic literature reviews of health state utility values: a descriptive analysis

**DOI:** 10.1186/s12874-022-01784-6

**Published:** 2022-11-25

**Authors:** Muchandifunga Trust Muchadeyi, Karla Hernandez-Villafuerte, Michael Schlander

**Affiliations:** 1 Division of Health Economics, German Cancer Research Center (DKFZ), Foundation Under Public Law, Im Neuenheimer Feld 280, 69120 Heidelberg, Germany; 2 Medical Faculty Mannheim, University of Heidelberg, Mannheim, Germany; 3 Health Economics, WifOR institute, Rheinstraße 22, Darmstadt, 64283 Germany; 4 Alfred Weber Institute for Economics (AWI), University of Heidelberg, Heidelberg, Germany

**Keywords:** Quality appraisal, Health state utility values, Preferences, Checklists, Critical appraisal, Risk of bias

## Abstract

**Background:**

Health state utility values (HSUVs) are an essential input parameter to cost-utility analysis (CUA). Systematic literature reviews (SLRs) provide summarized information for selecting utility values from an increasing number of primary studies eliciting HSUVs. Quality appraisal (QA) of such SLRs is an important process towards the credibility of HSUVs estimates; yet, authors often overlook this crucial process. A scientifically developed and widely accepted QA tool for this purpose is lacking and warranted.

**Objectives:**

To comprehensively describe the nature of QA in published SRLs of studies eliciting HSUVs and generate a list of commonly used items.

**Methods:**

A comprehensive literature search was conducted in PubMed and Embase from 01.01.2015 to 15.05.2021. SLRs of empirical studies eliciting HSUVs that were published in English were included. We extracted descriptive data, which included QA tools checklists or good practice recommendations used or cited, items used, and the methods of incorporating QA results into study findings. Descriptive statistics (frequencies of use and occurrences of items, acceptance and counterfactual acceptance rates) were computed and a comprehensive list of QA items was generated.

**Results:**

A total of 73 SLRs were included, comprising 93 items and 35 QA tools and good recommendation practices. The prevalence of QA was 55% (40/73). Recommendations by NICE and ISPOR guidelines appeared in 42% (16/40) of the SLRs that appraised quality. The most commonly used QA items in SLRs were response rates (27/40), statistical analysis (22/40), sample size (21/40) and loss of follow up (21/40). Yet, the most commonly featured items in QA tools and GPRs were statistical analysis (23/35), confounding or baseline equivalency (20/35), and blinding (14/35). Only 5% of the SLRS used QA to inform the data analysis, with acceptance rates of 100% (in two studies) 67%, 53% and 33%. The mean counterfactual acceptance rate was 55% (median 53% and IQR 56%).

**Conclusions:**

There is a considerably low prevalence of QA in the SLRs of HSUVs. Also, there is a wide variation in the QA dimensions and items included in both SLRs and extracted tools. This underscores the need for a scientifically developed QA tool for multi-variable primary studies of HSUVs.

**Supplementary Information:**

The online version contains supplementary material available at 10.1186/s12874-022-01784-6.

## Introduction

The concept of evidence-based medicine (EBM) originated in the mid-nineteenth century in response to the need for a conscientious, explicit, and judicious use of current, best evidence in making healthcare decisions [1]. Emerging from the notion of evidence-based medicine is the systematic and transparent process of Health Technology Assessment (HTA). HTA can be defined as a state-of-the-art method to gather, synthesize and report on the best available evidence on health technologies at different points in their lifecycle [2]. This evidence informs policymakers, insurance companies and national health systems during approval, pricing, and reimbursement decisions. As the world continues to grapple with increased healthcare costs (mainly due to an ageing population and the rapid influx of innovative and expensive treatments), health economic evaluations are increasingly becoming an integral part of the HTA process.

Comparative health economic assessments, mainly in the form of cost-effectiveness analysis and cost-utility analysis (CUA), are currently the mainstay tools for the applied health economic evaluation of new technologies and interventions [3]. Within the framework of CUA, the quality-adjusted life years (QALY) is a generic outcome measure widely used by economic researchers and HTA bodies across the globe [3]. Quality-adjusted life years are calculated by adjusting (multiplying) the length of life gained (e.g. the number of years lived in each health state) by a single weight representing a cardinal preference for that particular state or outcome. These cardinal preferences are often called health state utility values (HSUVs), utilities or preferences in the context of health economics.

Notably, HSUVs are regarded as one of the most critical and uncertain input parameters in CUA studies [4]. A considerable body of evidence on cost-effectiveness analyses suggests that CUA results are sensitive to the utility values used [3, 5, 6]. A small margin of error in the HSUVs used in CUA can be enough to alter the reimbursement and pricing decision and have far-reaching consequences on drug quality-adjusted life years, the incremental cost-effectiveness ratios, and may potentially impact an intervention’s accessibility [3, 5, 6]. Besides, HSUVs are inherently heterogeneous. Applying different population groups (patients, general population, caregivers or spouses, and in some instances, experts or physicians), context, assumptions (theoretical grounding), and elicitation methods may generate different utility values for the same health state [7–9]. Thus, selecting appropriate, relevant and valid HSUVs is germane to comparative health economic assessments [3, 4, 10].

The preferences reflected in the HSUVs can be directly elicited using direct methods such as the time trade-off (TTO), the standard gamble (SG) or the visual analogue scale (VAS) [11]. Alternatively, indirect methods using multi-attribute health status classification systems with preference scores such as the EuroQol- 5 Dimension (EQ-5D), Short-Form Six-Dimension (SF6D), Health Utilities Index (HUI) or mapping from non-preference-based measures onto generic preference-based health measures can also be employed [12]. However, methodological infeasibility, costs, and time constraints make empirical elicitation of HSUVs a problematic and sometimes an unachievable task. Consequently, researchers often resort to synthesising evidence on HSUVs through rapid or systematic literature reviews (SLRs) [12]. Correspondingly, the number of SLRs of studies eliciting HSUVs has been growing exponentially over the years, particularly in the last five years [13].

The cornerstone of all SLRs is the process of Quality Appraisal (QA) [14, 15]. Regardless of the source of utility values, HSUVs should be “free from known sources of bias, and measured using a validated method appropriate to the condition and population of interest and the perspective of the decision-maker for whom the economic model is being developed [4]”. The term garbage in garbage out (GIGO), originates from the information technology world, and is often referred to in quality discussions. The use of biased, low-quality HSUVs estimates will undoubtedly result in wrong and misleading outcomes, regardless of how robust the other elements of the model are. To avoid using biased estimates, it is imperative that empirical work on HSUVs, the reporting of such work, and subsequent reviews of studies eliciting HSUVs are of the highest level of quality. A robust, scientifically developed and commonly accepted QA tool is one step towards achieving such a requirement.

Over the years, some research groups and HTA agencies have developed checklists, ad-hoc tools, and good practice recommendations (GPRs) describing or listing the essential elements to consider when assessing the quality of primary studies eliciting HSUVs. Prominent among these GPRs are the International Society for Pharmacoeconomics and Outcomes Research (ISPOR) Taskforce report [16], the National Institute for Health and Care Excellence (NICE) Technical Document 9 [17], and related peer-reviewed publications [4, 10, 12, 18], hereafter referred to as”*NICE/ISPOR tools".* Despite this effort and the importance placed on HSUVs and their QA process, there is still no accepted gold standard, scientifically developed, and widely accepted QA tool for studies eliciting HSUVs.

Several challenges impede the critical appraisal of studies eliciting HSUVs. Common to all QA processes is the significant heterogeneity in using the term QA. This heterogeneity leads to a misunderstanding of and or disagreements on what should and should not constitute QA [19, 20]. The term quality represents an amorphous and multidimensional concept that should include the quality of reporting, methodological (e.g. risk of bias [RoB]) and external validity (applicability) [15, 21, 22]. However, it is often incompletely and or inappropriately applied by restricting quality only to a subset of its components (mostly one dimension). For example, many SLR authors use the term QA to refer to the RoB assessment [15, 23–25], while others refer to the reporting quality assessment [19, 26, 27]. Similarly, several terms to define QA have also been used interchangeably in the literature. These terms include: quality assessment, methodological quality, methodological review, critical appraisal, critical assessment, grading of evidence, data appropriateness, and credibility check [22]. Resultantly, the domains, components and or items considered to evaluate the studies' quality also vary considerably [22].

Another challenge in appraising the quality of studies contributing to SLRs is the lack of guidance for applying the QA results into the subsequent stages of a review, particularly summarizing and data synthesis; interpreting the findings, and drawing conclusions [14, 28]. The trend over the years has been shifting away from scale-based QA to domain-based RoB assessments [29, 30]. Moreover, there is no consensus regarding the quality threshold for the scale-based approach nor risk summary judgment for the domain-based approaches [28].

Specific to SLRs of studies eliciting HSUVs is the unique nature and characteristics of these studies, mainly study design. While randomised controlled trials (RCTs) are the gold standard for intervention studies of effect size [31], multiple study designs, including experimental (e.g. RCTs) and observational (e.g. cohort, case–control, cross-sectional) designs, can be used in primary studies on HSUVs [14]. On the one hand, RCTs may suffer from a lack of representation of the real-world setting, mainly due to strict inclusion and exclusion criteria (which is a form of selection bias). On the other hand, observational studies, by design, are inherently prone to several problems that may bias their results, for example, confounding or baseline population heterogeneity. While confounding is mainly controlled at the design stage through randomisation in RCTs, statistical and analytical methods are vital for controlling confounding in observational studies. More so, some QA items such as the randomisation process, blinding of investigators/assessors, description of the treatment protocol for both intervention and control groups and use of intention-to-treat analysis [22] tend to be more specific to RCTs of intervention studies and of less value to observational and or primary studies of HSUVs.

By design, all intervention studies of measure of effect size should ideally be comparative and define at least one intervention. The gold standard is to include a control or comparator group that is “equivalent” to the intervention group, with only the intervention under investigation varying. On the contrary, not all studies eliciting HSUVs are intervention and comparative studies. Oftentimes, HSUVs are elicited from the population of interest (or the whole population) without regard to an intervention. This distinction between primary studies of HSUVs and intervention studies presents another unique feature to primary studies of HSUVs. QA of empirical studies of HSUVs (except when there is an intervention in question) may not find QA items such as intervention measurement, adherence to prescribed intervention, randomisation, concealment of allocation, blinding of subjects, and outcomes relevant or feasible.

Furthermore, the various methodologies used to elicit utility values make it challenging to identify a QA tool that allows an adequate comparison between studies. Direct methods are frequently used alongside indirect methods [12]. Consequently, using a single QA tool is insufficient; however, it remains unclear if using multiple tools would remedy the above-mentioned challenges.

Few studies in the literature where QA tools were used reflected the previously described multi-factorial nature of the QA of studies eliciting HSUVs. More recently, Yepes-Nuñez et al. [13] summarised the methodological quality (examining RoB) of SLRs of HSUVs published in top-ranking journals. The review culminated in a list of 23 items (grouped in 7 domains) pertinent to the RoB assessment. Nevertheless, RoB is only one necessary quality dimension, by itself insufficient [15].

Ara et al. mentioned that a researcher needs a well-reported study to perform any meaningful assessment of other quality dimensions [10, 18]. Correspondingly, the completeness and transparency of the reporting (i.e., reporting quality dimension) is also needed. Similar to RoB, a focus on reporting quality without attention to RoB is also necessary, but alone, insufficient. Notably, an article can be of good reporting quality—reporting all aspects of the methods, presenting their findings in a clear and easy-to-understand manner—and still be subject to considerable methodological flaws that can bias the reported estimates [3, 32].

Since HSUVs as an outcome can be highly subjective and context-driven compared to the commonly assessed clinical outcomes in clinical effectiveness studies, limiting the QA of studies eliciting HSUVs to reporting and methodological quality dimensions is not enough (necessary but insufficient rule). The relevance and applicability (i.e., external validity) of the included studies also matter. Relevance and applicability questions are equally crucial to the decision-maker, including whose utility values and when and where the assessment was done.

Gathering evidence on the current practices of SLRs authors to appraise the quality of primary studies eliciting HSUVs is key to solving the above-mentioned challenges. It forms the precursor to the development, based on a systematic process, of a QA tool that assures a consistent and comparable evaluation of the evidence available. Therefore, the main objective of this study is to review, consolidate, and comprehensively describe the current (within the last five years) nature of QA (methodological, reporting and relevance) in SLRs of HSUVs. Given the challenges hampering QA of studies eliciting HSUVs, we hypothesise that many SLR authors are reluctant to perform QA; hence we expected a low prevalence. We also hypothesise that there is significant heterogeneity in how the QAs are currently done. We precisely aim at:

¬ Evaluating the prevalence of QA in published systematic reviews of HSUVs.
¬ Determining the nature of QA in SLRs of HSUVs.
¬ Exploring the impact of QA on the SLR analysis, its results, and recommendations.
¬ Identifying and listing all items commonly used for appraising the quality in the SLR of HSUVs and comparing these to items of existing checklist, tools and GPRs.
¬ Identifying and listing all checklists, tools and GPRs commonly used for QA of studies eliciting HSUVs

## Methodology

A rapid review (RR) of evidence was conducted to identify peer-reviewed and published SLRs of studies eliciting HSUVs from 01.01.2015 to 11.05.2021. Cochrane RRs guidelines were followed with minor adjustments throughout the RR process [33].

### Definition of terms

Table 1 defines some key terms applicable to quality and quality appraisal. Notably, since not all published QA tools have been validated, in this study, we define a standardised tool as a tool that has been scientifically developed and published with or without validation.

**Table 1:** Definitions of key terms related to quality appraisal

**Key terms**	**Definition**
**Critical Appraisal (CA)**	Critical appraisal is the process of **carefully** and **systematically** examining research to judge its **trustworthiness**, **value** and **relevance** in a particular context (Burls, [34]).
**Study quality**	Study quality is the extent to which a study is conducted to the highest methodological standards possible (Büttner et al., [29, 30]). Study quality is a multidimensional term referring to a set of parameters in **the design**, **conduct** and **reporting of a study** that reflects **the validity of the outcome**, related to the **external (relevance and applicability**) and **internal validity** and the **statistical model** used (Verhagen et al., [35]). Therefore, by assessing the study quality (Quality Assessment), one should be able to make informed judgements on a study’s **trustworthiness** and **its value** and **relevance** in a particular context.
**Reporting quality**	Reporting quality refers to the extent to which a set of parameters in the design and conduct of a study have been described to allow judgements on relevance and RoB. The purpose of reporting quality is to provide complete and transparent information about a study’s design, conduct, analysis, and results (Büttner et al., [29, 30]).
**Methodological quality**	Methodological quality or methodological review refers to the extent to which the study has been executed, for example, whether randomization or blinding (of participants and investigators) were done and how they were done. Notably, a randomized controlled trial (RCT) that cannot blind participants might be considered high-quality because it may be the only way for investigators to conduct such a RCT (Büttner et al., [29, 30]).
**Risk of bias (RoB)**	The term RoB is often used interchangeably with methodological quality or review, although the two terms are different. Bias is a systematic error, or deviation from the true findings, in results or inferences (which should not be confused with imprecision-a random error). Risk of bias refers to the likelihood that features of the study design or conduct will give misleading results or inferences. Notably, not all methodological shortcomings (low methodological quality) may result in biased estimates (or a high risk of bias)
**Quality checklist**	Quality checklists contain items that relate to study quality without assigning numeric values or producing a summary score (Büttner et al., [29, 30]).
**Quality scale**	Quality scales assign numeric values to scale items and combine information about several methodological features in a study to produce a summary score (Büttner et al., [29, 30]).
**Domain-based RoB tools**	Domain-based tools evaluate study limitations in specific domains that represent different biases. Example include bias arising from the randomization process or selection of participants into the study (Büttner et al., [29, 30]).
**Standardized tool**	A standardized tool is an instrument that is evidence-based, scientifically developed and tested for its psychometric properties (reliability, reproducibility, validity and feasibility). Therefore a standardized tool offers consistent procedures and uniform application, and it has the potential to compare findings across studies.
**Technical document**	A document containing information created to describe (in technical language) how the empirical elicitation of HSUVs or how the QA of HSUVs should be conducted. This can be in the form of recommendations or guidelines.

### Data sources and study eligibility

A search strategy adopted from Petrou et al. 2018 [12] that combines terms related to HSUVs, preference-based instruments and systematic literature reviews (SLRs) was run in the PubMed electronic database on 11.05.2021. The search strategy did not impose restrictions on the disease entity or health states, population, intervention, comparators and setting. All retrieved articles were exported to EndNote version X9 software (Clarivate Analytics, Boston, MA, USA), and duplicate cases were deleted. The remaining articles were exported to Microsoft Excel for a step-wise screening process. To ensure we did not miss any relevant articles, the PubMed search strategy was translated into Embase search terms and run on 05.09.2022. For example, we converted MeSH and other search terms to Emtree and replaced the PubMed-specific field codes with Embase-specific codes. All articles retrieved were exported to Microsoft Excel for a step-wise screening process. Search strings and hits for both databases are summarised in the Additional file 1, Supplementary Material 1 and 2, Table A.1 and A.2.

One author (MTM) developed the inclusion and exclusion criteria based on study objectives and previous reviews. All identified SLRs that performed a descriptive synthesis and or meta-analysis of primary HSUVs studies (direct or indirect elicitation) and were published in English from January, 01, 2015, to April, 29, 2021 were included. A pilot exercise was done on 50 randomly chosen titles and abstracts. Refinement of the inclusion and exclusion was done after this initial round of screening. Two experienced/senior health economists (KHV and MS) reviewed the inclusion and exclusion criteria with minor adjustments. The final inclusion and exclusion criteria is summarised in the Additional file 1, Supplementary Material 3, Table A.3.

### Data screening

A step-wise screening process starting with titles, followed by abstracts and the full text, was done by one reviewer (MTM) using the pre-developed inclusion and exclusion criteria. Full-text SLRs that matched the stage-wise inclusions and exclusion criteria (See Additional file 1, Supplementary Material 3, Fig A.1) were retained for further analysis. The reference lists of the selected SLRs were further examined to identify any relevant additional reviews, tools, and GPRs. MTM repeated the same steps as described above (i.e., title, abstract and full-text scan) based on the mentioned inclusion and exclusion criteria to identify additional articles from the reference list of the initially selected SLRs.

MTM and KHV discussed any uncertainty about including certain studies and mutually decided on the final list of included articles.

### Data extraction

A two-stage data extraction process was done using two predefined Microsoft Excel spreadsheet data extraction matrices. MTM designed the first drafts of the data extraction matrices based on a similar review [29, 30] and research objectives. KHV and MS reviewed both matrices with minor adjustments. First, all the relevant bibliography and descriptive information on the QA process done by the SRL authors were extracted (See Additional file 1, Supplementary Material 4, Table A.4a). One of our aims was to determine the prevalence of QA in the included SLRs. Therefore, we did not appraise the quality of included SLRs. Since high-quality SLRs must incorporate all the recommended review stages, including the QA stage; we assumed that including only high-quality SLRs may potentially bias our prevalence point estimates.

Second, all QA tools, checklists and GPRs, identified or cited in the included SLRs were extracted. A backward tracking was undertaken to identify all the original publications of these QA tools, checklists and GPRs. Authors’ names and affiliations, year of first use or publication, domains, items or signalling questions contained in each extracted QA tool, checklist and GPR were then harvested using the second data extraction sheet (see Additional file 1, Supplementary Material 4 Table A. 4b).

### Data synthesis

Narrative and descriptive statistics (i.e., frequencies, percentages, counterfactual acceptance rate [CAR], listing and ranking of items used) were performed on the selected SLRs and the identified QA tools, checklists and GPRs. All graphical visualizations were plotted using the ggplot2 package in R.

#### Descriptive analysis of included SLRs, checklists, tools and GPRs practices extracted

The SLRs were first categorised into those that performed a QA of the contributing studies or not. For those SLRs that appraised the quality of studies, descriptive statistics were calculated based on six stratifications: 1) QA appraisal tool type (i.e., an ad-hoc tool or custom-made, standardised or adapted tool); 2) critical assessment tool format (i.e., scale, domain-based or checklist); 3) QA dimensions used (i.e., reporting quality, RoB and/or relevancy); 4) how the QA results were summarised (i.e., summary scores, threshold summary score or risk judgments); 5) type of data synthesis used (quantitative including meta-analysis or qualitative), and 6) how QA results were used to inform subsequent stages of the analysis (i.e., synthesis/results and/or the conclusion-drawing). The distribution of the number of QA items and existing checklists, tools and GRPs used to generate these items were also tabulated (see Additional file 1, Supplementary Material 4, Table A.4a).

Similarly, QA tools, checklists, and GPRs extracted in the second step of the review were categorised according to 1) document type (i.e., technical document [recommendations], technical document [recommendations] with a QA tool added, a previous SLR, reviews, SLRs or standardised tool), 2) critical assessment tool format (i.e., domain-based, checklists or scale-based tools) and 3) QA dimensions included in the tool (i.e., any of RoB or methodological, reporting or relevancy dimension) and items as they are listed [original items] (see Additional file 1, Supplementary Material 4, Table A.4b).

#### Quality appraisal – Impact of QA on the synthesis of results

To explore the impact of the QA on the eligibility of studies for data synthesis, we first analysed the acceptance rate for each SLR that used the QA assessment results to exclude articles. We defined the acceptance rate of a SLR as the proportion of primary studies eliciting HSUVs that meet a predetermined (by the SLR’s authors) quality threshold. The threshold can be presented as a particular score for scale-based or an overall quality rating (e.g., high quality) for domain-based QA.

Second, a counterfactual analysis was done on a subset of SLRs that appraised the quality of contributing studies but did not incorporate the QA's results in the data synthesis. A counterfactual acceptance rate (CAR) was defined as the proportion of studies that would have been included if the QA results had informed such a decision. Based on a predetermined QA threshold, we defined the counterfactual acceptance rate as follows:

1 $$CAR =\frac{ number\ of\ studies\ with\ quality\ >\ 60\mathrm{\% }\ (or\ a \ high-quality\ rating\ in\ all\ domains)}{total\ number\ of\ eligible\ studies.}$$

In the SLR by Marušić et al. [14], the majority (52%, *N* = 90) of included SLRs used a quality score as a threshold to inform which primary studies qualify for data synthesis. A quality threshold of 3 out of 5 (60%) was used for the Jadad [36] and Oxford [14] scales and 6 out of 9 (67%) for the Newcastle–Ottawa scale. Consequently, we used a quality threshold of 60% in the CAR calculations (see Eq. 1). Reporting checklists with Yes, No, and Unclear responses were converted into a scale-based (Yes = 1, No = 0 and Unclear = 0). The resulting scores were summed to calculate the overall score percentage.

Regarding domain-based tools, the ROBINS-I tool [37] gives guidelines to make summary judgments of the overall RoB as follows: 1) a study is judged "low" risk of bias if it scores "low" in all RoB domains; 2) a study is judged "moderate" if it scores "moderate" to "low" in any of the RoB domains; 3) a study is judged "serious risk of bias” if scores "serious or critical" in any domain. By so doing, the tools assume that any RoB domain could contribute equally to the overall RoB assessment. On the contrary, the Cochrane RoB tool [28] requires review authors to pre-specify (depending on outcomes of interest) which domains are most important in the review context. In order to apply the Cochrane RoB, it is necessary to first rank the domains according to their level of importance. The level of importance, thus the ranking, depends on both the research question and context. A context-based ranking approach would be highly recommendable. However, given that the relevant SLR articles refer to different contexts, it was not feasible to establish an informed and justified ranking of the domains for each article based on context. Therefore, while considering that the context-based approach is highly desirable, we chose the method applied in the ROBINS-I tool [37] to evaluate the CAR of SLRs that used domain-based ratings and did not provide a summary judgment.

#### Quality appraisal – items used and their relative importance

We separately extracted and listed all original QA items: 1) used in the SLRs and 2) found in the original publications of QA tools, checklists and GPRs cited, adapted or customised by the SLR authors of included reviews. Based on a similar approach used by Yepes et al. [13], we iteratively and visually inspected the two mentioned lists for items that used similar wording and or reflected the same construct. Where plausible and feasible, we retained the original names of the items as spelt out in QA tools, checklists and GPRs or by the SLR authors. A new name or description was assigned to those items that used similar wording and or reflected the same constructs. For example, we assigned the name ‘missing (incomplete) data’ for all original items phrased as ‘incomplete information’, ‘missing data’, and ‘the extent of incomplete data’. Similarly, items reflecting preference elicitation groups, preference valuation methods, scaling methods, and or choice versus feeling-based methods were named ‘technique used to value the health states (see Additional file 1, Supplementary Material 5a; Table A.5 and Table A.6 for the assignment process). In this way, apparent discrepancies in wording, spellings and expressions in the items were matched. All duplicate items and redundancies were concurrently removed. A single comprehensive list of items used in SLRs or extracted QA tools, checklists and GPRs was produced (see Additional file 1, Supplementary Material 5a; Table A.7).

Using the comprehensive list of the items with assigned names, we counted the frequency of occurrence of each item included in 1) the SLRs of studies eliciting HSUVs and 2) identified QA tools, checklists and GPRs. We regard the frequency of each item in SLRs as a reasonable proxy to the relative importance that SLR authors place on the items. Similarly, the frequency of occurrence in QA tools and GRPs can be regarded as a reasonable proxy for what items are valued more highly in the currently existing tools that are commonly used for QA of studies eliciting HSUVs.

Additionally, we narrowed the above analysis to two selected groups of items: 1) one composed of the 14 items corresponding to the recommendations by the ISPOR Taskforce report [16], NICE Technical Document 9 [17] and related peer-reviewed publications [4, 10, 12, 18] (hereafter referred to as ‘ISPOR items’), and 2) an additional list of 14 items (hereafter as ‘Additional items’ (see Additional file 1, Supplementary Material 5b and 5c). Additional items were informed mainly by literature [38], theoretical considerations [39–45] and the study team’s conceptual understanding of HSUV elicitation process. Specifically, Additional items represent those that we considered "relevant" (based on the literature and theoretical considerations and were not included in the ISPOR items). For example, statistical consideration and the handling of confounders do not appear in the ISPOR items, yet they are relevant to the QA of studies eliciting HSUVs. We considered the combination of both lists (28 combined items) to be a comprehensive but not exclusive list of items that can be deemed "relevant" to QA of contributing studies to the SLR of studies eliciting HSUVs. Correspondingly, the frequency of ISPOR items in SLRs can be considered a reasonable proxy measure of the extent to which SLR authors are following the currently existing GPRs, while the frequency of the Additional items as a proxy of the importance of other “relevant” items in the QA process. The frequency of the ISPOR items and the Additional items in the existing QA tools, checklists and GPRs can be considered a proxy measure of how well the currently used tools covered the “relevant” items for the QA of studies eliciting HSUVs (i.e., suitability of purpose).

All analyses on SLRs that appraised quality were further stratified by considering separately: 1) the 16 SLRs [9, 26, 46–59] that either adapted or used one or more of the 6 QA tools, checklists and GPRs were considered to be NICE, ISPOR and related publications report [4, 10, 12, 16–18] (hereafter ‘*QA based on NICE/ISPOR tools’*) and 2) the 24 SLRs that adapted, customised or used other QA tools, checklists and GPRs (hereafter “*QA based on other tools*”). Similarly, all analyses on QA tools, checklists and GPRs were further stratified by considering separately: 1) the 6 QA tools, checklists that are considered to be NICE, ISPOR and related publications [4, 10, 12, 16–18] (hereafter *‘NICE/ISPOR tools’*) and 2) 29 QA tools, checklists and GPRs (hereafter “*Other tools*”).

## Results

**Fig. 1:**
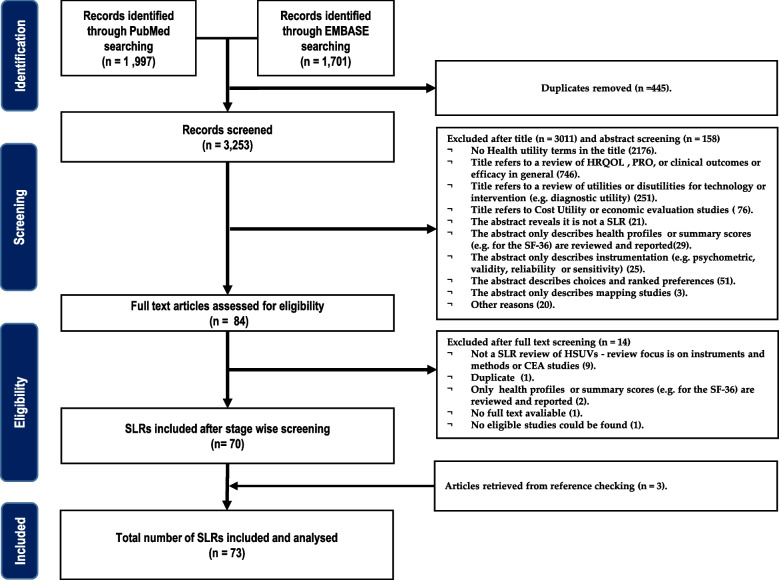
PRISMA flow diagram summarising the study selection process. HRQOL, Health Related Quality of Life; PRO, Patient Reported Outcomes; CEA, cost-effectiveness analysis; CUA, cost-utility analysis; PRISMA, Preferred Reporting Items for Systematic Review and Meta-Analyses; SLR, Systematic Literature Review

The initial electronic search retrieved 3,253 records (1,997 from PubMed and 1,701 from Embase). After the initial step-wise screening process, 70 articles were selected. Three additional articles were retrieved from the snowball method of selecting relevant articles identified from the chosen SLRs. Thus in total, 73 SLRs were analysed (see Fig. 1).

### Characteristics of included SRLs, checklists, tools and GRPs

The SLRs included in the analysis consist of utility values for health states covering a wide range of disease areas: cardiovascular diseases (10%); neurological diseases, including Alzheimer’s disease, mild cognitive impairment and dementia (10%); cancers of all types (21%); infectious diseases, including human immunodeficiency virus and tuberculosis (10%); musculoskeletal disorders, including rheumatoid arthritis, osteoporosis, chronic pain, osteoarthritis, ankylosing spondylitis, psoriatic arthritis, total hip replacement, and scleroderma (6%); metabolic disorders, including diabetes (3%); gastrointestinal disorders (4%); respiratory disorders (non-infectious) including asthma (4%) and non-specific conditions, including injuries and surgeries (20%). Special attention was also given to mental health and childhood utilities which accounted for 1% and 10% of the eligible SRLs (see Additional file 1, Supplementary Material 6, Table A.8).

Table 2 shows the characteristics of the QA tools, checklists and GPRs used to evaluate the quality of studies eliciting HSUVs in the SLRs analysed. A total of 35 tools, checklists and GPRs were extracted directly from the SLRs analysed. Most of these (37%) were standardised tools that are scientifically developed for QA of either RCTs or observational studies. Technical documents, which merely seek guidance on appraising quality, accounted for another 37%. Notably, a few SLRs (8%) of studies eliciting HSUVs [60–63] based their QA appraisal methods on those used in previous SLRs [21, 64, 65] or reviews [66, 67], in which the authors of those SLRs had used guidance from a previous SLR [68].

**Table 2:** Characteristics of 35 QA tools, checklists and GPRs

**Description of data item analysed**	**QA tools and GPRs identified**	
	***N* = 35**	**%**
**Document type**^a^		
Reviews	1	2.9%
Technical documents (recommendations) with a QA tool developed or added	5	14.3%
Technical documents (recommendations)	8	22.9%
Systematic literature reviews (SLRs)	8	22.9%
Standardized tools	13	37.1%
**NICE/ISPOR tool**		
Yes	6	17.1%
No	29	82.9%
**Critical assessment format**^a^		
Domain based (ranking)	9	25.7%
Checklist	7	20.0%
Scale based	6	17.1%
Not specific	1	2.9%
NA	12	34.3%
**QA dimensions incorporated**^b^		
Rob only	12	34.3%
Reporting quality only	4	11.4%
Rob and relevancy	2	5.7%
Reporting and Rob	3	8.6%
Reporting, Rob (methodological) and relevancy	9	25.7%
NA	5	14.3%

Source: Authors’ elaboration

*QA* Quality appraisal, *NA* Not applicable, *Rob* Risk of bias, *SLR* Systematic Literature review, *GPR* Good practice recommendations

^a^Definition of terms explained in Table 1

^b^Additional details on the definition of each category is provided in Additional file 1, Supplementary material 4, Table A.4

Regarding the critical assessment format (see Table 1 for the definition of terms), domain-based tools contributed 26% to the total number of tools, checklists and GPRs extracted. In comparison, checklist and scale-based tools accounted for 20% and 17%, respectively, representing 37%. (see Additional file 1, Supplementary Material 6, Table A.9, for more details on the 35 QA tools and GPRs).

### Prevalence and characteristics of QA in included SLRs

Table 3 shows the prevalence of QA and the current nature of QA in the included SLRs. The number of QA tools and GPRs used or cited ranged from 1 to 9 (equal mean and median of 2 and IQR of 1). Notably, the observed prevalence of QA is 55%. Around a third of the SLR authors (33%) used all three QA dimensions (reporting, RoB [methodological] and relevancy) to appraise the quality of studies eliciting HSUVs. Of the 40 SLRs that appraised quality, 16 (42%) were based on NICE/ISPOR tools [9, 26, 46–59].

**Table 3:** Prevalence and characteristics of QA in included SLRs

**Description of data item analysed**	**SLRs**	
	**#**	**%**
**1. All studies included in the review (*N* = 73)**		
**Prevalence of quality appraise**		
Appraised the quality of contributing studies	40	54.8%
Did not appraise quality of contributing studies	33	45.2%
**2. A subset of studies that appraised quality of individual studies (*N* = 40)**		
**Critical assessment tool type**		
Custom-made (ad-hoc)	16	40.0%
Adapted existing tool(s)	13	32.5%
Standardized tool	7	17.5%
Both standard and a custom-made tool	2	5.0%
Both adapted and a custom-made tool	1	2.5%
Not reported	1	2.5%
**Based on NICE/ISPOR tools**		
Yes	16	40.0%
Other tools and good practice recommendations	24	60.0%
**Critical assessment format**		
Scale (score based)	12	30.0%
Checklist	9	22.5%
Domain based	11	27.5%
Both checklist and domain based	2	5.0%
Both scale and checklist	2	5.0%
Both scale and domain based	3	7.5%
Not reported	1	2.5%
**Quality appraisal dimensions used**		
Reporting quality only	7	17.5%
RoB only	9	22.5%
Relevancy only	2	5.0%
RoB and Relevancy	3	7.5%
RoB and Reporting	6	15.0%
Reporting, RoB (Methodological) and Relevancy	13	32.5%
**Use of QA results to inform data synthesis and conclusions**		
No attempt to incorporate quality assessment findings into systematic review findings	15	37.5%
Narrative discussion (with minimal evidence for incorporation deemed acceptable)	18	45.0%
Sensitivity analysis	1	2.5%
Exclude studies at high or unclear risk of bias (or moderate or low quality) from the synthesis	5	12.5%
Unknown	1	2.5%
**Type of data synthesis**		
Qualitative (descriptive) synthesis	20	50.0%
Quantitative synthesis (meta-analysis or regression)	4	10.0%
Both Qualitative and Quantitative	16	40.0%
**Distribution of the number identified QA tools, checklists and GPRs per SRL included**	#	
Median	2	
Mean	2	
Q1	1	
Q3	2	
Minimum	1	
Max	9	
IQR	1	
**Distribution the number items used to appraise quality per SRL included**		
Median	8	
Mean	10	
Q1	7	
Q3	13	
Minimum	1	
Max	30	
IQR	6	

Source: Authors’ elaboration

*IQR* Interquartile range, *Q1* 25^th^ percentile value, *Q2* 50^th^ percentile (median), *Q3* 75^th^ % percentile value, *QA* Quality appraisal (or assessment), *SLR* Systematic literature review

### Impact of the QA on study outcomes

The 40 studies that appraised quality included 1,653 primary studies eliciting HSUVs, with the number of included studies ranging from 4 to 272 (median = 28, mean = 41 and IQR = 33). Surprisingly, most (35/40) SLRs that appraised the quality of their included studies did not use the QA findings to synthesise final results and overall review conclusions. Of the remaining five articles, three [47, 60, 62] used the QA results to inform the inclusion of studies for meta-analysis (acceptance rate was 100% for Afshari et al. [60] and Jiang et al. [62], and 53% for Blom et al. [47]). These represent only 15% (3/20) of the studies that performed a quantitative synthesis (i.e., meta-analysis or meta-regression). In the fourth [50] and fifth study [69], the QA results were used as a basis of inclusion for the qualitative synthesis, with 33% and 67% of the eligible studies being included in the final analysis.

We estimated the counterfactual acceptance rate (CAR) for those SLRs that appraised the quality of contributing studies but did not incorporate the QA's results in the data synthesis. Six of the 40 SLRs [48, 53, 55, 56, 70, 71] did not provide sufficient information to calculate the threshold or summarize the judgement of risk of bias. For the other 6 studies [47, 50, 60, 62, 69, 72], the actual acceptance rate was as reported by the SLR authors. CAR in the remaining 28 SRLs ranged from 0 to 100% (mean = 53%, median = 48% and IQR 56%).

**Fig. 2:**
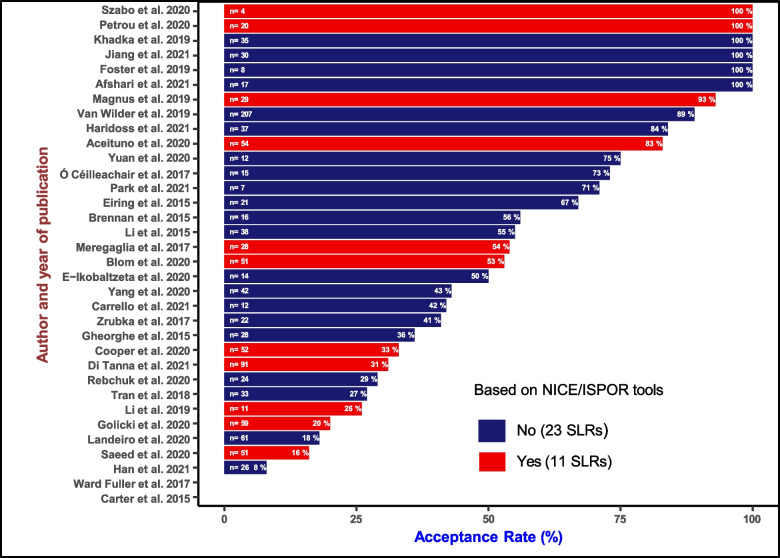
Counterfactual acceptance rates (CAR) across the SLRs evaluated. Note: For Blom et al. [47], Copper et al. [50], Afshari et al. [60], Jiang et al. [62], Etxeandia-Ikobaltzeta et al. [72] and Eiring et al. [69], the actual acceptance rates reported by authors are presented. *n* = xx represents the total number of articles considered eligible and evaluated for quality after screening. SLRs = Systematic Literature Reviews

If all the 28 SLRs for which a CAR was estimated had considered the QA results, on average, 57% of 1053 individual studies eliciting HSUVs would have been deemed ineligible for data synthesis. Had the 28 SLRs used QA results to decide on the inclusion of studies for the analysis stage, 52% (15/28) would have rejected at least 50% of the eligible studies. Figure 2 shows the estimated CAR and acceptance rates across the 32 analysed studies.

### Items used for the QA of primary studies in the included SLRs

The majority of the included SLRs (39/40) comprehensively described how the QA process was conducted. One study [70] mentioned that QA was done but did not describe how it was implemented. Furthermore, the terminology used to describe the QA process varied considerably among the SLRs. Terms such as quality appraisal or assessment [9, 23, 24, 48, 49, 51, 53, 55, 57–60, 73–78], critical appraisal [47], risk of bias assessment [25, 62, 63, 72, 79–82], relevancy and quality assessment [52, 56], assessment of quality and data appropriateness [50], methodological quality assessment [26, 27, 46, 54, 61, 69], reporting quality [71, 83] credibility checks and methodological review [70] were used loosely and interchangeably. One study [84] mentioned three terms, RoB, methodological quality and reporting quality, in their description of the QA process. Notably, most SLRs that used the term quality assessment incorporated all three QA dimensions (RoB [methodology], reporting and relevance) in the QA.

A comprehensive list of 93 items remained after reviewing the original list of items, assigning new names where necessary, and removing duplicates (see Additional file 1, Supplementary Material 5a Table A.7). Only 70 out of the 93 items found a place in the 40 SLRs that appraised the quality of studies eliciting HSUVs. The number of items used per SLR ranged from 1 to 29 (mean = 10, median = 8, and IQR = 8).

Of the 70 items used in SLRs, only five were used in at least 50% of the 40 SLRs: ‘response rates’ (68%); ‘statistical and/or data analysis’ (55%), ‘loss to follow-up [attrition or withdrawals]’ (53%), ‘sample size’ (53%) and ‘missing (incomplete) data’. Some of the least frequently used items include: ‘sources of funding’, ‘administration procedures’, ‘ethical approval’, ‘reporting of *p*-values’, ‘appropriateness of endpoints’, ‘the generalizability of findings’ and ‘non-normal distribution of utility values’. Each was used in only one SLR (3%). Twenty-three items (23/93) were not used in the SLRs but appeared in QA tools, checklists and/or GPRs. Some of these include ‘allocation sequence concealment’, ‘questionnaire response time’ ‘description and use of anchor states’, ‘misclassification (bias) of interventions’, ‘reporting of adverse events’, ‘the integrity of intervention’ and ‘duration in health states’ (see Additional file 1, Supplementary material 7, Table A.10).

**Fig. 3:**
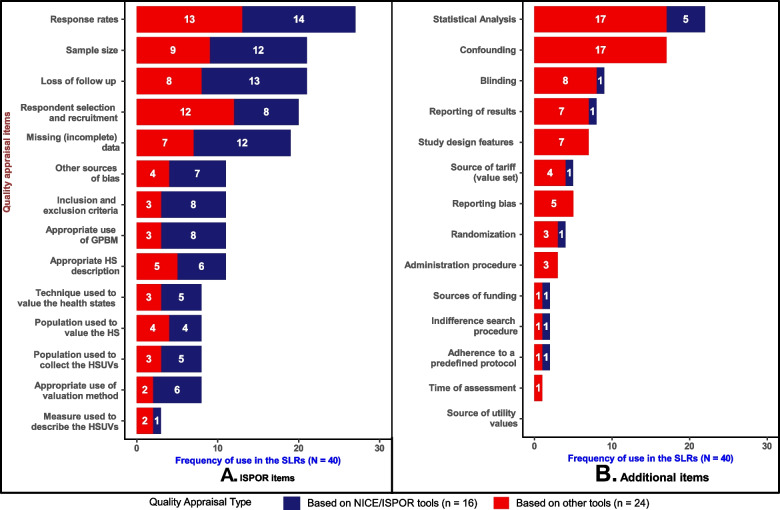
Frequency of use of ISPOR and Additional items in SLRs. GPBM, generic preference-based measure; HS, health states and HSUVs, Health state utility values

Results of the ISPOR and Additional items are depicted in Fig. 3. The ISPOR item (Panel A) that most frequently occurred in SLRs was ‘response rates’ (27/40). Notably, most SLRs that evaluated ‘response rates’ developed their QA based on NICE/ISPOR tools (14/27). Similarly, QA based on NICE/ISPOR tools tended to include items such as ‘sample size’ (12 vs 9), ‘loss of follow up’ (13 vs 8), ‘inclusion and exclusion criteria’ (8 vs 3) and ‘missing data’ (12 vs 7) more so than those based on other checklists, tools and GPRs. Moreover, among ISPOR items, the measure used to describe the health states appeared the least frequently (3/40) in the SLRs. Additionally, none of the 40 SLRs evaluated all the 14 ISPOR items, and 10 of these items were considered by less than 50% of the SLRs. This observed trend indicated that adherence to the currently published guidelines is limited.

Similar to the ISPOR items, most of the Additional items (Panel B) were used in just a few SLRs, with 12 appearing in less than 25% of the SLRs. The Additional item that appeared most frequently was ‘statistical and/or data analysis’ (22/40). Five out of these 22 articles were SLRs that based their QA on NICE/ISPOR tools. Items related to administration procedures’, ‘indifferent search procedures’ and ‘time of assessment’ were the least used, each appearing only one to three times out of the 40 SLRs analysed. Of note, no SLR that based its QA on NICE/ISPOR tools included items related to ‘confounding and baseline equivalence'; study design features; ‘reporting biases and administration procedure, which were used in 17, 9, 5 and 3 of the 40 SLRs, respectively. The figure also suggests that QA, based on other currently existing QA tools, checklists, and GPRs, focused more on statistical and data analysis issues (17 vs 5) and blinding (8 vs 1).

### Items occurring in the checklists, tools, and GRPs extracted from the SLRs

Out of the 93 items identified, 81 items appeared in the identified checklists, tools, and GPRs (see Additional file 1, Supplementary Material 7, Table A.11). The most frequently featured items were ‘statistical/data analysis’ (23/30) and ‘confounding or baseline equivalency of groups’ (20/30). The least occurring items included instrument properties (feasibility, reliability, and responsiveness), ‘generalisability of findings’, ‘administration procedure’ and ‘ethical approval’, all of which were featured once. Twelve items (12/93) were not found in the checklists, tools, and GRPs, for instance, ‘bibliographic details (including the year of publication)’, ‘credible extrapolation of health state valuations’, and ‘source of tariff (value set)’.

**Fig. 4:**
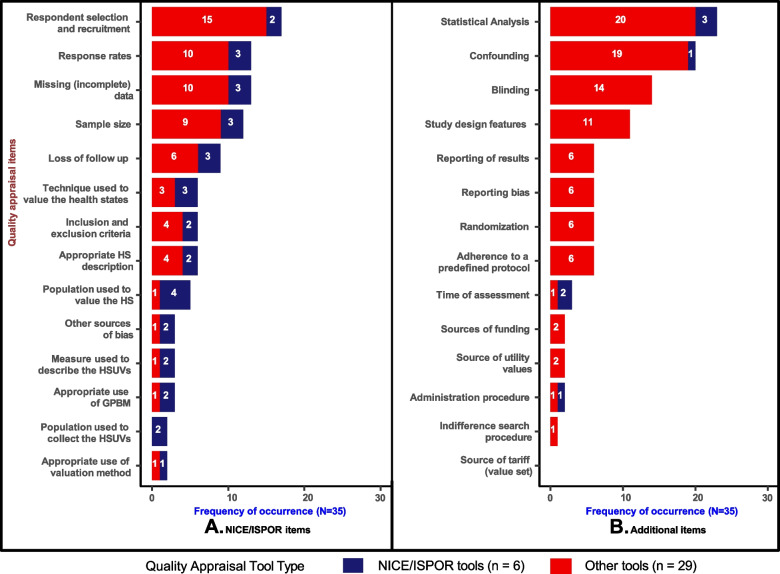
Frequency of occurrence in QA tools, checklist, and good practice recommendations. GPBM, generic preference based measure; HS, health states and HSUVs, Health state utility values

Figure 4 shows the occurrence frequency of ISPOR (Panel A) and Additional items (Panel B) in the 35 QA tools, checklists and GPRs. Notably, each ISPOR item featured in less than 50% (18) of the 35 QA tools, checklists, and GPRs analysed. The most frequently appearing ISPOR item was ‘respondent and recruitment selection’ (17/35), followed by ‘response rates (13/35) and ‘missing or incomplete date’ 13/35, and ‘sample size’ (11/30). The most frequently occurring Additional item was ‘statistical/data analysis’ (23/35), which appeared in 3 out of the 6 of the NICE/ISPOR tools and 20 out of the 29 other checklists, tools and GPRs. This was followed by confounding (20/35), which also appeared in only 1 out of the 6 NICE/ISPOR tools. Remarkably, items such as ‘blinding’ (14/35), ‘study design features’ (11/35) and ‘randomisation’ (6/35) only appeared in other checklists, tools and GPRs which are not considered NICE/ISPOR tools.

**Fig. 5:**
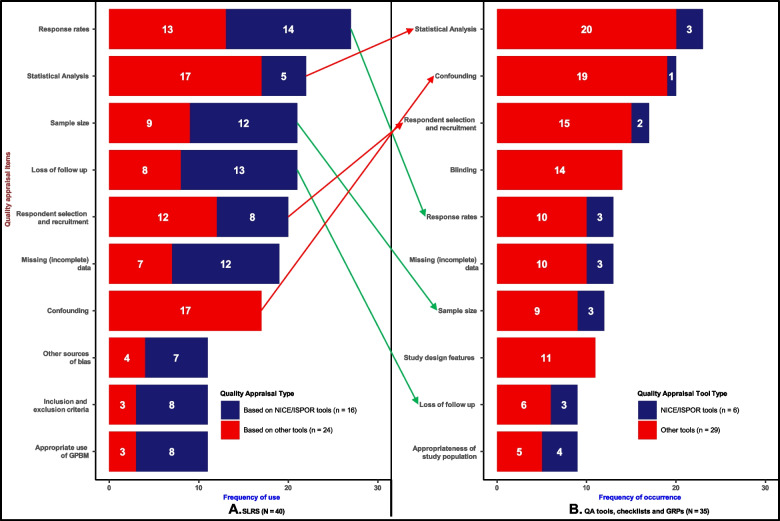
Top ten most occurring items in (**A**) SLRs and (**B**) QA tools, checklists and GPRs. GPBM, generic preference-based measure; HS, health states and HSUVs, Health state utility values

Out of the 93 items from the comprehensive list, Fig. 5 displays the ten most used items in SLRs (Panel A) and the ten most frequently occurring items in the QA tools, checklists, and GPRs analysed (Panel B). On the one hand, although ‘blinding’ and ‘study/experimental design features’ were not among the ten most frequent items in the SLRs, they were highly ranked among the QA tools, checklists, and GPRs (fourth [with 40% occurrence rate] and eighth [with 31% occurrence rate], respectively). On the other hand, items related to ‘response rates’ and ‘loss of follow up’ had a higher ranking among the SLRs (first [68%] and third [53%], respectively) than among the checklists, tools and GPRs (seventh [33%] and tenth [26%] and respectively).

## Discussion

We reviewed 73 SLRs of studies eliciting HSUVs and comprehensively described the nature of QA undertaken. We identified 35 QA tools, checklists, and GPRs considered or mentioned in the selected SLRs and extracted their main characteristics. We then used the two sets of information to generate a comprehensive list of 93 items used in 1) SLRs (70 items) and 2) in the QA tools, checklists, and GPRs (81 items) (see Additional file 1, Supplementary file 5).

With only 55% of SLRs appraising the quality of included studies, the results supported our hypothesis of a low prevalence of QA in SLRs of studies eliciting HSUVs. This is evident when compared to other fields such as sports and exercise medicine, in which the prevalence of QA in SLRs was 99% [30], general medicine, general practice public health and paediatrics (90%) [15], surgery, alternative medicine rheumatology, dentistry and hepatogastroenterology (97%), and anesthesiology 76% [14]. In these fields, the high prevalence is in part linked to the availability of standardised QA tools and the presence of generally accepted standards [15, 30]. For instance, a study on sports and exercise medicine [30] estimated that standardised QA tools were used in 65% of the SLRs analysed compared to 16% in the current study. The majority of the SRLs in the Büttner et al. [30] study were either healthcare interventions (32/66) or observational epidemiology (26/66) reviews, where standardised QA tools are widely available and accepted. Examples include: the Jadad Tool [36], Downs and Black [85], Newcastle–Ottawa Scale (NOS) scale, Cochrane tool for RoB assessment tools [28, 86], RoB 1 [37] and RoB 2 [87].

Our results showed that SRL authors incorporate heterogeneous QA dimensions in their QAs. These variations can be attributed to a strong and long-standing lack of consensus on the definition of quality and the overall aim of doing a QA [31]. Overall, the present review identified three QA dimensions, RoB, reporting and relevancy\applicability, which were evaluated to varying extents (see the breakdown in Table 2). This heterogeneity in dimensions often leads to considerable variations in the QA items considered and the overall conclusions drawn [22, 38]. For instance, Büttner et al. [29, 30] compared the QA results based on the Downs and Black checklist^1^ and the Cochrane Risk of Bias 2 tool (RoB2).^2^ Interestingly, QA using the RoB 2 resulted in 11/11 of the RCTs being rated high overall RoB, while using the Downs and Black checklist resulted in 8/11 of the same studies being judged as high-quality trials.

The result from the study by Büttner et al. [29, 30] described above is in favour of focusing only on RoB when appraising the quality of studies included in a SLR. Nevertheless, additional challenges exist when the studies are not well reported. It is different from concluding that a study is prone to RoB because it had several methodological flaws and that another is prone to RoB because the reporting was unclear. In effect, we do not know anything about the RoB in a study that does not provide sufficient details for such an assessment.

Pivotal to any QA in a SLR process is the reporting quality of included studies. A well-reported study allows reviewers to judge whether the results of primary studies can be trusted and whether they should contribute to meta-analyses [14]. First, the reviewers should assess the studies' methodological characteristics (based on the reported information). Only then, based on the methodological rigour (or flaws) identified, should risk judgements, the perceived risk that the results of a research study deviate from the truth [29, 30], be inferred. Inevitably, all three quality dimensions are necessary and sufficient components of a robust QA [88].

A challenge to the QA of studies eliciting HSUVs is the apparent lack of standardised and widely accepted QA tools to evaluate them. First, this is evident in some of the SLRs [89–92] that did not appraise the quality of contributing studies and cited a lack of a gold standard as the main barrier to conducting such. Second, most of the SLRs that appraised quality did this by customising elements from the different checklist(s) [24, 27, 75, 79, 80], or using standardised tools designed to evaluate quality in other types of studies, and not primarily for eliciting HSUVs [23, 27, 52, 62], and GPRs [9, 26, 46, 47, 50, 54–56, 61, 63, 74, 84]. In this regard, we estimated that SLR authors used, on average, two QA tools, checklists or GPRs (Max = 9) to construct their customised QA tools, with only 14/40 (35%) SLRs using one tool [24, 25, 49, 51, 53, 57–60, 73, 75, 79, 80, 82]. This finding is not consistent with other fields of research. For instance, Katikireddi et al. [15] conducted a comprehensive review of QA in general practice public health and paediatrics. Their study estimated that, out of the 678 selected SLRs, 513 (76%) used a single quality/RoB assessment tool. Tools used included the non-modified versions of the Cochrane tool for RoB assessment (36%), the Jadad tool (14%), and the Newcastle–Ottawa scale (6%) [15].

The observed use of multiple tools leads to a critical question regarding the appropriateness of combining or developing custom-made tools to address the challenges present in the QA in SLRs of HSUVs studies. Petrou et al.’s guide to conducting systematic reviews and meta-analyses of studies eliciting HSUVs stated that "*In the absence of generic tools that encompass all potentially relevant features, it is incumbent on those involved in the review process to describe the quality of contributing studies in holistic terms, drawing where necessary upon the relevant features of multiple checklists*” [12]. While this may sound plausible and pragmatic to many pundits, it requires comprehension and an agreement on what should be considered “relevant features”. Here is where the evidence delineated in this comprehensive review may call into question the notion of Petrou et al. [12].

The analysis of the comprehensive list of 93 items (see Fig. 5 and Additional file 1, Supplementary Material 7 Table A.10 and A.11) showed: 1) a high heterogeneity among the QA items included in the SLRs and 2) a considerable mismatch of what is included in the existing QA tools, checklists and GPRs — which may be relevant for those who created the tools and the specific fields they were created for — with what is used by SLR authors in the QA of studies eliciting HSUVs.

The plethora of QA tools that authors of SLRs can choose from are designed with a strong focus on healthcare intervention studies measuring effect size. Yet, primary studies of HSUVs are not restricted to intervention studies. Accordingly, features that could be considered more relevant to intervention studies than to studies eliciting HSUVs such as the blinding of participants and outcomes, appeared in 40% of the checklists and GPRs and did not appear in any of the QAs of studies eliciting HSUVs. Their exclusion could indicate that the SLRs authors omitted less "relevant” features.

However, authors of SLRs overlooked an essential set of core elements of the empirical elicitation of HSUVs. For instance, Stalmeier et al. [39] provided a shortlist of 10 items necessary to report in the methods sections of studies eliciting HSUVs. The list includes items on how utility questions were administered, how health states were described, which utility assessment method or methods were used, the response and completion rates, specification of the duration of the health states, which software program (if any) was used, the description of the worst health state (lower anchor of the scale), whether a matching or choice indifference search procedure was used, when the assessment was conducted relative to treatment, and which (if any) visual aids were used. Similarly, the Checklist for Reporting Valuation Studies of Multi-Attribute Utility-Based Instruments (CREATE)[43]—which can be considered to be very close to HSUVs elicitation—includes attribute levels and scoring algorithms used for the valuation process. Regrettably, core elements such as instrument administration procedures, respondent burden, construction of tasks, indifferent search procedures, and scoring algorithms were used in less than 22% of the SLRs (see Fig. 3). The lack of these core elements strongly suggests that existing tools may not be suitable for QA of empirical HSUVs studies.

Additionally, the most highly ranked items in existing tools are statistical analysis and confounding and baseline equivalence, which appeared in 66% and 57%, of QA tools, checklists and GPRs evaluated. These items are only used in 55% and 43% of the SLRs that appraised quality. Undeniably, studies eliciting HSUVs are not just limited to experimental and randomised protocols, where the investigator has the flexibility to choose which variables to account for and control for during the design stage. It becomes extremely relevant to control for confounding variables in HSUVs primary studies (both observational and experimental) and employ robust statistical methods to control for any remaining confounders.

Furthermore, several items found in currently existing checklists, tools and GPRs reviewed and used by SLR review authors may be considered redundant. These include, as examples, ‘items on sources of funding’, ‘study objectives and research questions’, ‘bibliographic details, including the year of publication’ and ‘reporting of ethical approval’.

Another argument against Petrou et al.’s recommendation to resort to multiple QA tools when customising existing tools for QA in SLRs of studies eliciting HSUV is the need for consistency, reproducibility and comparability of research. Consistency, reproducibility and comparability are key to all scientific methods and or research regardless of domain. Undeniably, using multivariable QA tools and methods, informed by many published critical appraisal tools and GPRs (35 in our study), does not ensure consistency, reproducibility and comparability of either QA results or overall conclusions [21, 22].

The 14 ISPOR items drawn from the few available GPRs specific for studies eliciting HSUVs [4, 5, 10, 16–18, 93] and the 14 Additional items which were informed mainly by literature [38], theoretical considerations [39–45] and the study team’s conceptual understanding of HSUV elicitation process can be considered a plausible list of items to include when conducting QA of studies eliciting HSUVs. Nevertheless, besides the list being too extensive or broad, there is, and would be high heterogeneity in the contribution of these items to QA. Therefore, there is a strong need for a scientific and evidence-based process to streamline the list into a standardised one and hope that it can be widely accepted.

Although SLRs and checklists, tools, and GPRs shared the same top five ISPOR items (i.e., response rates, loss of follow up, sample size, respondent selection and recruitment, and missing data), the ISPOR items are more often considered in the SLRs than they appear in checklists, tools, and GPRs reviewed. Moreover, our results showed that Additional items, which are also valuable in QA, have a considerably lower prevalence than ISPOR items in the QA presented in the SLRs. This is of concern since relying only on NICE/ISPOR tools may overlook relevant items for the QA of studies eliciting HSUVs such as ‘statistical or data analysis’, ‘confounding’, ‘blinding’, ‘reporting of results’, and ‘study design features’. Arguably too, relying on the current set of the QA tools, checklist and GPRs that have a noticeable lack of attention (as implied by their low frequency of occurrence) to items that capture the core elements for studies eliciting HSUVs, such as techniques used to value health states, the population used to collect the HSUVs, appropriate use of valuation method, and proper use of generic preference-based methods will not address the present challenges.

Another critical area where SLR authors are undecided is which QA system to use. While the guidelines seem to favour domain-based over the checklist and scale-based systems, SLR authors still seem to favour checklist and scale-based QA, presumably due to their simplicity. Our results suggest that scale-based checklists were used in more than 66% of the SLRs that appraised quality. The pros and cons of either system are well documented in the literature [15, 21, 22, 38]. Notably, the two systems will produce different QA judgements [15, 21, 22, 38]. The combined effect of such heterogeneity and inconsistencies in QA is a correspondingly wide variation and uncertainty in the QA results, conclusions and recommendations for policy.

Our analysis also revealed an alarmingly low rate of SLRs in which the conducted QA impacts the analysis. Congruent with previous studies in other disciplines of general medicine, public health, and trials of therapeutic or preventive interventions [15, 94, 95], only 11% (5/35) of the SLRs that conducted a QA explicitly informed the synthesis stage based on the QA results [47, 50, 60, 62, 69]. The reasons for this low prevalence of incorporating QA findings into the synthesis stage of SLRs remain unclear. However, it could be firmly attributed to a lack of specific guidance and disagreements on how QA results can be incorporated into the analysis process [95].

Commonly used methods for incorporating QA results into the analysis process include sensitivity analysis, narrative discussion and exclusion of studies at high RoB [15]. The five SLRs in our review [47, 50, 60, 62, 69] excluded studies with high or unclear risk of bias (or moderate or low quality) from the synthesis. These findings are a cause of concern since the empirical evidence suggests that combining evidence from low-quality (RoB) articles with high-quality leads to bias in the overall review conclusions, which can be detrimental to policy-making [15]. Therefore, incorporating the QA findings into the synthesis and conclusion drawing of any SLR [28–30], mainly of HSUVs, which are heterogeneous and considered a highly sensitive input parameter in many CUA [3, 5, 6], is highly recommendable. Nevertheless, the lack of clear guidance and agreement on how to do so remains a significant barrier.

To explore the potential impact of QA, we calculated counterfactual acceptance rates for individual studies and corresponding summary statistics (mean, median and IQR). While there has been an increasing number of empirical studies eliciting HSUVs over the years, our results suggest that a staggering 46% of individual studies would be excluded from the SLRs analysis because of their lower quality. However, this needs to be interpreted with caution. First, there is a mixed bag of QA tools (reporting quality vs methodological flaws and RoB, domain-based vs scale-based). Second, there could be an overlap of individual primary studies across the 40 SLRs that appraised quality. Third, although informed by previous studies, the QA threshold we used is arbitrary. There is currently no agreed standard or recommended threshold cut-off point to use during QA. This has resulted in considerable heterogeneity on the threshold used to exclude studies for synthesis in the previous literature [14]. Forth, there are variations in approaches recommended by different tools on how to summarise the individual domain ratings into an overall score [14, 15].

Two main strengths can be highlighted in our review. First, in comparison to Yepes-Nuñez et al. [13], who focused on RoB and included 43 SRLs (to our knowledge, the only review that looked at RoB items considered in the QA of SLRs studies eliciting HSUVs), our findings are based on a larger sample (73 SLRs) with a broader focus (three dimensions: RoB, reporting, and relevancy\applicability) [13]. Second, in addition to examining QA in SRLs, we systematically evaluated the original articles related to each of the 35 identified checklists, tools, and GPRs [13]. Consequently, our comprehensive list of items reflects the QA methods applied in the SLRs and the current practices applied in checklists, tools, and GPRs. More importantly, based on both types of articles (i.e., SLRs and checklists, tools and GPRs), we propose a subsample of 28 main items that can serve as the basis for developing a standardised QA tool for the evaluation of HSUVs.

A limitation of our study is that the understanding of how QA was done was solely based on our comprehension of the reported information in the SLRs. Since this was a rapid review, we did not contact the corresponding SLR authors for clarifications regarding extracted items and QA methodology. A second limitation is that the SLRs were selected from published articles between 2015 and 2021. We adopted this approach to capture only the recent trends in the QA of studies on HSUVs, including the current challenges. Furthermore, the review by Yepes-Nuñez et al. [13], which reviewed all SLRs of HSUV from inception to 2015, has been used as part of the evidence that informed the development of the “Additional items”. As a result, our list captured all the 23 items identified by Yepes-Nuñez et al. and considered relevant before 2015.

## Conclusions

Our comprehensive review reveals a low prevalence of QA in identified SLRs of studies eliciting HSUVs. Most importantly, the review depicts wide inconsistencies in approaches to the QA process ranging from the tools used, QA dimensions, the corresponding QA items, use of scale- or domain-based tools, and how the overall QA outcomes are summarised (summary scores vs risk judgements). The origins of these variations can be attributed to an absence of consensus on the definition of quality and the consequent lack of a standardised and widely accepted QA tool to evaluate studies eliciting HSUVs.

Overall, the practice of QA of individual studies in SLRs of studies eliciting HSUVs is still in its infancy stage. There is a strong need to promote QA in such assessments. The use of a rigorously and scientifically developed QA tool specifically designed for studies on HSUVs will, to a greater extent, ensure the much-needed consistency, reproducibility and comparability of research. A key question remains: Is it feasible to have a gold standard, comprehensive and widely accepted tool for QA of studies eliciting HSUVs? Downs and Black [85] concluded that it is indeed feasible to create a "checklist" for assessing the methodological quality of both randomised and non-randomised studies of health care interventions.

Therefore, the next step to developing a much-needed QA tool in the field of HSUVs is for researchers to reach a consensus on the working definition of quality, particularly for HSUVs where contextual considerations matter. Once that is established, an agreement on the core dimensions, domains and items that can be used to measure the quality, based on the agreed concept of quality, then follows. This work provides a valuable pool of items that should be considered for any future QA tool development.

## Supplementary Information

**Additional file 1.**

## Data Availability

All data is provided in the paper or supplementary material.

## Abbreviations

CAR: Counterfactual Acceptance Rate
CUA: Cost-Utility Analysis
EQ-5D: Euroqol- 5 Dimension
GPBM: Generic Preference-Based Methods
GPR(s): Good Practice Recommendation(s)
HSUVs: Health States Utility Value(s)
HTA: Health Technology Assessment
HUI: Health Utilities Index
IQR: Interquartile Range
ISPOR: The Professional Society for Health Economics And Outcomes Research
NICE: The National Institute for Health and Care Excellence
QA: Quality Appraisal or Quality Assessment
RCT(s): Randomised Controlled Trial(s)
RoB: Risk of Bias
ROBINS-I: Risk Of Bias In Non-Randomised Studies of Interventions
RR(s): Rapid Review(s)
SF6D: Short-Form Six-Dimension
SG: Standard Gamble
SLR(s): Systematic Literature Review(s)
TTO: Time Trade-Off
VAS: Visual Analogue Scale

## Footnotes

1 The checklist is comprised of 27 items across four subscales, including completeness of reporting (9 items); internal validity (13 items); precision (1 item), and; external validity (3 items).

2 RoB2 is the revised, second edition of the Cochrane Risk of Bias tool for RCTs with five RoB domains [1) bias arising from the randomisation process; 2) bias due to deviations from intended interventions; 3) bias due to missing outcome data; 4) bias in measurement of the outcome; and 5) bias in the selection of the reported results.
