# Supplementary material for: Quality appraisal for systematic literature reviews of health state utility values: a descriptive analysis
Source: BMC Med Res Methodol. 2022 Nov 25;22:303. doi: 10.1186/s12874-022-01784-6 (PMC9700894; doi:10.1186/s12874-022-01784-6)
Supplement: Supplementary file 1 — Additional file 1. [file 12874_2022_1784_MOESM1_ESM.pdf]

Quality appraisal for systematic literature reviews of health state utility values: A descriptive analysis
Additional File

Muchandifunga Trust Muchadeyi[1, 2], Karla Vanessa Hernandez-Villafuerte[1], Michael Schlander[1, 2]

[1] Division of Health Economics, German Cancer Research Center (DKFZ), Heidelberg, Germany
[2] Medical Faculty Mannheim, University of Heidelberg, Mannheim, Germany

* Corresponding author:

Muchandifunga Trust Muchadeyi
Division of Health Economics
German Cancer Research Center (DKFZ), ,
Im Neuenheimer Feld 280,
69120 Heidelberg,
Germany;
Email: m.muchadeyi@dkfz-heidelberg.de

Contents

Supplementary material 1- Literature Search in PubMed ... 4

a. Combined new search strategy ... 4

Supplementary material 2- Literature Search in Embase ... 6

Supplementary material 3: Inclusion and exclusion criteria ... 8

Supplementary material 4: Data extraction matrix ... 11

Supplementary material 5: Quality appraisal – Items used and their relative importance ... 13

a. Assignment of new names to items extracted from SLRs, QA tools and GRPs ... 13

b. Items recommended by NICE, ISPOR and related publications (ISPOR items) ... 42

c. Additional items considered "relevant" based on literature, theoretical considerations and conceptual understanding of HSUV elicitation ... 43

Supplementary material 6: Characteristics of included SLRs, tools and good recommendation practices ... 44

Supplementary material 7: Frequency of occurrence of items in SLRs, checklists, tools and GPRs . 63

References ... 69

Table of figures

Table A.1: Search strategy in PubMed 4

Table A.2: Search strategy in Embase 6

Table A.3: Inclusion & Exclusion Criteria, based on PRISMA Guidelines 8

Table A.4 List of items for data abstraction 11

Table A.5: List of original items in SLRs included and assigned names by each SLR: 13

Table A.6: List of original items and assigned names per each tool and GPR 28

Table A.7: Comprehensive list of items found in SLRs, QA tools, checklists and GPRs 41

Table A.8: Detailed characteristics of included SLRs 44

Table A.9: Detailed characteristics of the tools and good practice recommendations commonly used for QA in SLR of HSUVs 58

Table A.10: Frequency of use of QA items in SLRs that appraised quality 63

Table A.11: Frequency of occurrence of QA items in checklist, tools and GPRs 66

List of figures

Figure A.1: Screening Algorithm for included studies. 10

Supplementary material 1- Literature Search in PubMed

Table A.1: Search strategy in PubMed

Search category	Field	Search terms	Hits
**Utilities**	Title	1. Utilit* or disutilit* or HSUV* or "utility elicitation" or "health related utilities" or "health state utility valu*" or "utility preference*" or "utility score*" or "patient preference*" or "Utility assessment" or "Utility" or "Health state utility" or "Health utility"	36,917
**Indirect valuation methods**	Title or abstract	2. EQ-5D or EQ?5D or "EQ 5D" or EQ5D or Euroqol or "Euro qol" or EQ-5D-Y or "EQ 5D Y"<br>3. "short form 6D" or "SF-6D" or "SF 6D" or "SF6D" or "SF?36" or "RAND-36"<br>4. "health utilities index" or HUI<br>5. "quality of well being" or "quality of well-being" or QWB<br>6. "16D Health-Related Quality of Life" or "16D HRQoL" or "17D Health-Related Quality of Life or 17D HRQoL"<br>7. "AQoL?6D" or "Assessment of Quality of Life-6D" or "aqol 6d"<br>8. "Child Health Utility 9 Dimension" or CHU9D or CHU-9D or "CHU?9D" or "CHU 9D"<br>9. "15-dimensional instrument" or "15 dimensional instrument" or "15D"<br>10. "preference-based measure of HRQoL" or "preference based measure of HRQoL" or "preference?based measure"<br>11. "multi-attribute utility instrument" or "multiattribute utility instrument" or "multi attribute utility instrument" or "MAUI*" or "multiattribute?utility instrument"	12 582<br>23 144<br>1 849<br>382<br>1<br>30<br>67<br>2 061<br>168<br>427
**Direct valuation methods**	Title or abstract	12. "Standard Gamble" or "standard-gamble"<br>13. "Time trade off" or "time trade-off" or "time trade*" or "TTO"<br>14. "visual analog scales" or "visual analog scale" or "VAS"	870<br>2 037<br>67 075
**Combine 1 to 14 with OR and apply filters (2015.01.01 and Systematic reviews and English)***			**1 997** *(1994 after removing duplicates in Endnote)*

*Search was performed on 11.05.2021

a. Combined new search strategy

(("utilit*"[Title] OR "disutilit*"[Title] OR "hsuv*"[Title] OR "utility elicitation"[Title] OR "health related utilities"[Title] OR "health state utility valu*"[Title] OR "utility preference*"[Title] OR "utility score*"[Title] OR "patient preference*"[Title] OR "Utility assessment"[Title] OR "Utility"[Title] OR "Health state utility"[Title] OR "Health utility"[Title] OR ("eq 5d"[Title/Abstract] OR "eq 5d"[Title/Abstract] OR "eq 5d"[Title/Abstract] OR "EQ5D"[Title/Abstract] OR "Euroqol"[Title/Abstract] OR "Euro qol"[Title/Abstract] OR "eq 5d y"[Title/Abstract] OR "eq 5d y"[Title/Abstract]) OR ("short form 6D"[Title/Abstract] OR "sf

6d"[Title/Abstract] OR "sf 6d"[Title/Abstract] OR "SF6D"[Title/Abstract] OR "SF?36"[Title/Abstract] OR "RAND-36"[Title/Abstract]) OR ("health utilities index"[Title/Abstract] OR "HUI"[Title/Abstract]) OR ("quality of well-being"[Title/Abstract] OR "quality of well-being"[Title/Abstract] OR "QWB"[Title/Abstract]) OR "16D Health-Related Quality of Life"[Title/Abstract] OR ("AQoL?6D"[Title/Abstract] OR "AQoL?6D"[Title/Abstract]) OR ("CHU9D"[Title/Abstract] OR "chu 9d"[Title/Abstract] OR "chu 9d"[Title/Abstract] OR "chu 9d"[Title/Abstract]) OR ("15-dimensional instrument"[Title/Abstract] OR "15-dimensional instrument"[Title/Abstract] OR "15D"[Title/Abstract]) OR "preference?based measure"[Title/Abstract] OR ("multi-attribute utility instrument"[Title/Abstract] OR "multiattribute?utility instrument"[Title/Abstract] OR "multi-attribute utility instrument"[Title/Abstract] OR "maui*"[Title/Abstract] OR "multiattribute?utility instrument"[Title/Abstract]) OR ("standard gamble"[Title/Abstract] OR "standard gamble"[Title/Abstract]) OR ("visual analog scales"[Title/Abstract] OR "visual analog scale"[Title/Abstract] OR "VAS"[Title/Abstract]) OR ("time trade off"[Title/Abstract] OR "time trade off"[Title/Abstract] OR "time trade*"[Title/Abstract] OR "TTO"[Title/Abstract])) AND ("systematic review"[Filter] AND 2015/01/01:3000/12/12[Date - Publication] AND "english"[Language]) AND ("systematic review"[Filter] AND 2015/01/01:3000/12/12[Date - Publication] AND "english"[Language])) AND ((systematicreview[Filter]) AND (2015/1/1:3000/12/12[pdat]) AND (english[Filter]))

Supplementary material 2- Literature Search in Embase

Table A.2: Search strategy in Embase

Search Category or Term	Field	Search Terms	Hits
Utility Terms		'utility value'/exp OR 'utility value'<br>'utility value':ti,ab,kw<br>'health state utility value'/exp OR 'health state utility value'<br>'health state utility value':ab,ti,kw<br>'patient preference'/exp OR 'patient preference'<br>'patient preference':ab,kw,ti<br>'utility preference*':ab,kw,ti<br>'utility elicitation':ti,ab,kw<br>'utility assessment':ti,ta,ab,kw<br>'health related utilities':ti,ta,ab,kw<br>**Combine all with OR and no filters**	**31 737**
Indirect Valuation Method Terms	EQ-5D	'european quality of life 5 dimensions questionnaire'/exp OR 'european quality of life 5 dimensions questionnaire'/de<br><br>'european quality of life 5 dimensions 3 level questionnaire'/exp OR 'european quality of life 5 dimensions 3 level questionnaire'<br><br>'european quality of life 5 dimensions 5 level questionnaire'/exp OR 'european quality of life 5 dimensions 5 level questionnaire'<br><br>'eq 5d questionnaire':ta,ti,ab,kw<br>'eq 5d':ti,ta,ab,kw<br>eq5d:ta,ab,kw<br>'eqol 5d':ta,ab,kw<br>'eq-5d-y':ta,ab,kw<br>'eq 5d y':ta,ab,kw<br>**Combine all with OR**	**26 296**
	SF36 terms	'short form 6d'/exp OR 'short form 6d'<br>'short form 36'/exp OR 'short form 36'<br>'short form 6d':ti,ta,ab,kw<br>'sf 6d':ti,ta,ab,kw<br>'sf6d':ti,ta,ab,kw<br>'sf?36':ti,ta,ab,kw<br>'rand 36':ti,ta,ab,kw<br>**Combine all with OR**	**64 799**
	HUI	'health utilities index'/exp OR 'health utilities index'<br>'health utilities index mark 3'/exp OR 'health utilities index mark 3'<br>'health utilities index mark 2'/exp OR 'health utilities index mark 2'<br>'health utilities index mark iii'/exp OR 'health utilities index mark iii'<br>'health utilities index':ti,ta,ab,kw<br>**Combine all with OR**	**1064**
	QWB	'quality of well being scale'/exp OR 'quality of well being scale'<br>'quality of well-being':ti,ta,ab,kw<br>'quality of well being scale':ti,ta,ab,kw<br>qwb:ti,ta,ab,kw<br><br>**Combine all with OR**	**489**

	16Dand 17D	‘16d health-related quality of life':ti,ta,ab,kw<br>'16d hrqol':ti,ta,ab,kw<br>'17d health-related quality of life':ti,ta,ab,kw<br>'17d hrqol':ti,ta,ab,kw<br>**Combine all with OR**	**4**
	Assessment of Quality of Life-6D	'assessment of quality of life-6d'<br>'assessment of quality of life-6d':ti,ta,ab,kw<br>'aqol?6d':ti,ta,ab,kw<br>'aqol 6d':ti,ta,ab,kw<br>**Combine all with OR**	**55**
	CHU 9D	'child health utility 9 dimension'<br>'child health utility 9 dimension':ti,ta,ab,kw<br>chu9d:ti,ta,ab,kw<br>'chu 9d':ti,ta,ab,kw<br>'chu?9d':ti,ta,ab,kw<br><br>**Combine all with OR**	**118**
	15-dimensional instrument	“15-dimensional instrument”<br>'15-dimensional instrument':ti,ta,ab,kw<br>'15d':ti,ta,ab,kw<br><br>**Combine all with OR**	**3 191**
		“preference-based measure of HRQoL”<br>'preference-based measure of hrqol':ti,ta,ab,kw<br>'preference based measure of hrqol':ti,ta,ab,kw<br><br>**Combine all with OR**	**5**
	MAUI	'multi-attribute utility'/exp OR 'multi-attribute utility'<br>'multi-attribute utility':ti,ta,ab,kw<br>'multi attribute utility':ti,ta,ab,kw<br>'multiattribute utility':ti,ta,ab,kw<br>maui:ti,ta,ab,kw<br>**Combine all with OR**	**804**
Direct valuation	Standard Gamble	'standard gamble'/exp OR 'standard gamble'<br>'standard gamble':ti,ta,ab,kw<br>'standard-gamble':ti,ta,ab,kw<br>**Combine all with OR**	**1 190**
	TTO	'time trade-off method'/exp OR 'time trade-off method'<br>'time trade-off method':ti,ta,ab,kw<br>'time trade-off':ti,ta,ab,kw<br>tto:ti,ta,ab,kw<br>**Combine all with OR and no filters**	**3 103**
	VAS	'visual analog scale'/exp OR 'visual analog scale'<br>'visual analog scale':ti,ta,ab,kw<br>'visual analogue scale':ti,ta,ab,kw<br>'visual analog scaling':ti,ta,ab,kw<br>**Combine all with OR and no filters**	**130 306**
Combine all with OR without filters			241 205
Combine all with OR and apply filters (2015.01.01 and full text and Systematic reviews and English and Humans)		(#1 OR #2 OR #3 OR #4 OR #5 OR #6 OR #7 OR #8 OR #9 OR #10 OR #11 OR #12 OR #13 OR #14) AND ([systematic review]/lim OR [meta analysis]/lim) AND [article]/lim AND [humans]/lim AND [english]/lim AND [2015-2021]/py<br><br>(#1 OR #2 OR #3 OR #4 OR #5 OR #6 OR #7 OR #8 OR #9 OR #10 OR #11 OR #12 OR #13 OR #14) AND ([cochrane review]/lim OR [systematic review]/lim OR [meta analysis]/lim) AND [article]/lim AND [humans]/lim AND [english]/lim AND ([embase]/lim OR [medline]/lim OR [pubmed-not-medline]/lim) AND [01-01-2015]/sd NOT [01-05-2021]/sd AND [2015-2021]/py	2190<br><br><br><br><br>1710

Supplementary material 3: Inclusion and exclusion criteria

The PICOS framework based on the research question is a widely used inclusion and exclusion exit criteria. Nevertheless, based on our research question, this criteria do not apply to our case.

1. Population of interest (P): We aim to disentangle the nature of QA in SLRs of HSUVs regardless of the population of interest, disease condition, specific health states, target group, or any setting.
2. Intervention (1) and comparators (C): One of the unique features of primary studies of HSUVs is that not all studies eliciting HSUVs are intervention and comparative studies. As a result, not all of them must have an intervention or a comparator. Unlike clinical effectiveness studies, where randomized controlled trials are a gold standard and are entitled to describe a comparator and intervention arm, primary studies of HSUVs can be observational studies from the population of interest (or the whole population) without regard to an intervention or treatment. We comprehensively described the unique features of primary studies of HSUVs in the introduction section on the last paragraph, page 9 of the introduction section.
3. Outcome measure (O): The outcome measure of this review is health states utility values.
4. Time (T): All SLRs published between 01.01.2015 and 29.04.2021 were included.

Overall, the study will include all SLRs that performed a descriptive synthesis and or meta-analysis of primary HSUVs studies (direct or indirect elicitation) and were published in English from January 01, 2015, to April 29, 2021.

Table A.3: Inclusion & Exclusion Criteria, based on PRISMA Guidelines

**Stage of screening**	**Inclusion**	**Exclusion**
**Title**	1. Mention terms related to HSUV:<br>¬ Utility or disutilities<br>¬ Preferences, patient preferences<br>¬ Patient values,<br>¬ Outcome important<br>¬ Utility weight<br>¬ QALY weights<br><br>2. Utility values are for health states or outcomes resulting from intervention or treatment.	1. Title just relates to HRQOL without being specific to HSUV.<br>2. Utilities or disutilities for technology or intervention e.g. clinical utility of MRI scan.<br>3. Reviews of Cost utility analysis or full health economic evaluation.<br>4. Duplicates.<br>5. Reviews of Cost utility analysis or full health economic evaluation.
**Abstract**	**Aim:**<br><br>¬ Described in relation to systematic review of HSUVs.<br><br>**Methods:**	1. Not a SLR.<br>2. The aim and objective of the review is not for HSUV<br>3. Review relates to HRQOL or PROs without being specific to HSUV<br>4. Only health profiles or summary scores e.g. for the sf36 are reviewed and reported.

	¬ Inclusion criteria is restricted to studies that aim to report on studies reporting on elicitation of HSUV using direct or indirect instrument.<br>¬ Identifies the instruments for measuring or elicitation of HSUV of interest to the review.<br>**Results:**<br>¬ Summary of reviewed HSUVs.<br>¬ Summary of pooled results (means medians ranges etc.<br>¬ Qualitative description of utility values for given health states.	5. Utilities or disutilities for technology or intervention e.g. clinical utility of MRI scan.<br>6. Review relates to ordinal patient preferences (choices)<br>7. Cost utility or economic evaluation studies.<br>8. Review relates to instrument related issues (e.g. validity, reliability and sensitivity).<br>9. Review of mapping studies only.
**Full text**	1. SLR summarises and or describes primary studies of HSUVs of forgiven health states.	1. Not a review of HSUVs for example:<br>¬ Review focus is on instruments and methods.<br>¬ Review only reports on CEA /CUA without description of HSUVs.

HSUV, Health states utility values; QALY quality adjusted life years; HRQOL, health related quality of life; MRI, magnetic resonance imaging; SLR, systematic literature review; PRO, patient reported outcomes; CEA, Cost effectiveness analysis; CUA, cost utility analysis

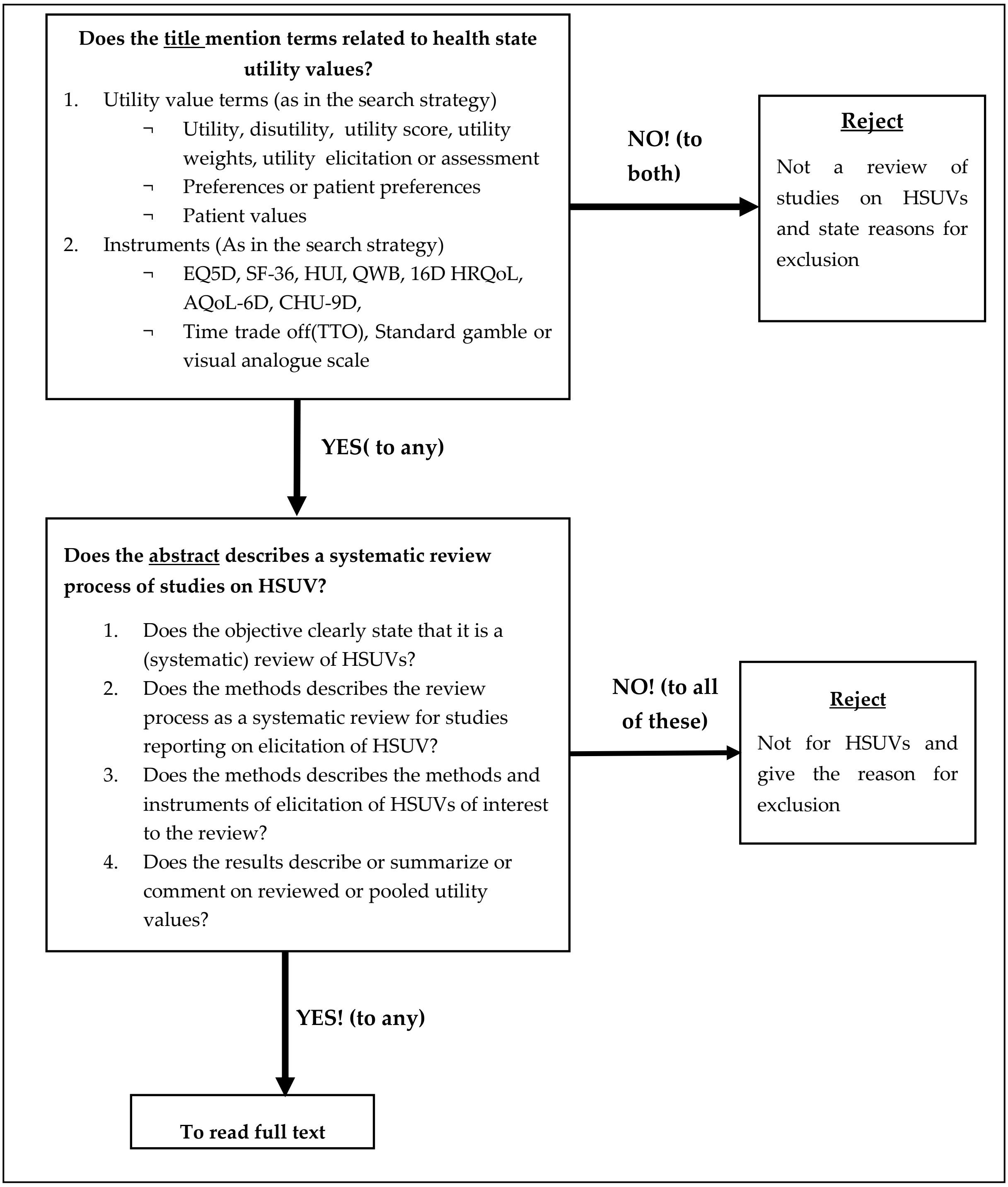

Figure A.1: Screening Algorithm for included studies.

HSUVs, Health states utility values; EQ5D Euroqol- 5 Dimension; SF-36 Short-Form Six-Dimension; HUI; Health Utilities Index; QWB, quality wellbeing; 16D; HRQoL, health related quality of life; AQoL-6D, assessment of quality of life six dimension; CHU-9D, child health utility nine dimensions

Supplementary material 4: Data extraction matrix

Table A.4 shows the list and description of data abstraction matrix separate for SLRs and of good practice recommendations, tools and checklist extracted.

Table A.4 List of items for data abstraction

**4.a Data extraction variables for included SLRs**	
**Outcome variables extracted**	**Levels of each outcome variable**
Author	Cooper, J. T., et al.
Year	2020
Title	
Objectives	
Disease area	
Instruments included in the review	
Was quality appraisal done?	Yes<br>No<br>Unclear
Tools, checklist or recommendation cited	1. Brazier J et al 2019: Identification, review, and use of health state utilities in cost-effectiveness models: an ISPOR good practices for outcomes research task force<br>2. Papaioannou D et al 2010: NICE Decision Support Unit Technical Support Document 9.
Quality assessment tool used (or developed)	**Adapted tool**: Systematic review authors used a section or part of published tool that suits their study requirements.<br>**Custom or Ad-hoc tool**: Systematic review authors used a combination of two or more tools and or recommendations to develop their own tool.<br>**Standard tool**[1]: Systematic review authors used a published tool in its entirety.
Based on NICE/ISPOR tools	Yes if based on any of Ara et al.2017; Braizer al.2011; Brazier et al.2019; Papaioannou et al. 2010 and 2013; Petrou et al. 2018 and Wolowacz et al.2015 publications<br>No if based on other QA tools, checklists and GPRs
Critical assessment tool format	**Scale**: Multiple tool items were assigned a quantitative value and combined to generate a single numeric summary score.<br>**Checklist**: Tool items possessed a descriptive, discrete outcome (e.g., “yes”, “no”, “maybe”) without an assigned numeric value.<br>**Domain-based tool**: Separate assessments were made for different risk of bias domains, with subjective judgements of “low risk”, “unclear”, and “high risk” determined by established criteria.
Quality dimensions evaluated	Reporting<br>RoB<br>Relevancy<br>Any mix of the above
List of QA items in the specific SLR	The smallest element of a tool that can be measured as single entity. For example sample size, response rates, loss to follow up
Number of items	
Number of identified QA tools, checklists and GPRs	
How were QA results summarized?	**Summary score**: Tool items were summed to generate a single numeric value.

[1] A standardized tool by definition is one that is evidence based, scientifically developed and tested for its psychometric properties (reliability, reproducibility, validity and feasibility). Therefore a standardized tool offers consistent procedures and uniform application, has the potential to compile and compare findings across studies. Here we refer to a tool that has e been scientifically developed and published with or without psychometric testing.

	**Threshold summary score**: Tool items were summed to generate a single numeric value with arbitrary cut-off values used to classify studies as being at 'low', 'moderate'/ 'unclear', or 'high' quality/risk of bias.<br>**Risk judgment:** An overall risk of bias or quality grade is determined based on the individual domain summary judgments.
Method used to incorporate critical assessment findings into systematic review findings	Sensitivity analysis<br>Meta-regression<br>Exclude studies at high or unclear risk of bias (or moderate or low quality) from the synthesis<br>Narrative discussion (with minimal evidence for incorporation deemed acceptable)<br>No attempt to incorporate risk of bias assessment findings into systematic review findings
**4.b Data extraction variables for good practice recommendations, tools and checklist extracted**	
Author	Cooper, J. T., et al.
Authors' affiliations	
Year of original publication	2020
Title of original publication	
NICE/ISPOR tool	Yes if a cited tool belong to any of Ara et al.2017; Braizer al.2011; Brazier et al.2019; Papaioannou et al. 2010 and 2013; Petrou et al. 2018 and Wolowacz et al.2015<br>No if Other QA tools, checklists and GPRs
Document type	Reviews<br>Technical Documents (Recommendations) with a QA tool developed or added<br>Technical Documents (Recommendations)<br>Systematic literature reviews(SLRs)<br>Standardized Tools
Critical assessment tool format	Domain based (Ranking)<br>Checklist<br>Scale based<br>Not specific<br>NA
QA dimensions included in the tool	RoB only<br>Reporting quality only<br>RoB and Relevancy<br>Reporting and RoB<br>Reporting, RoB(Methodological) and Relevancy<br>NA
Original items	

QA, Quality appraisal or assessment; GPRs, Good practice recommendations; NICE, The National Institute for Health and Care Excellence; ISPOR, The Professional Society for Health Economics And Outcomes Research; SLR, Systematic literature review

Supplementary material 5: Quality appraisal – Items used and their relative importance

a. Assignment of new names to items extracted from SLRs, QA tools and GRPs

Much heterogeneity in naming the same item, which may lead to some items being redundant or duplicated, was observed on the initial comprehensive list of items generated from the SLR that appraised quality. We resolved this by listing all the items as they were presented in the SLRs and the tools, checklist, and GPRs. After familiarising all the items, we iteratively identified common and or related items and described them with a more "standardised" nomenclature. Where plausible and feasible, we retained the original names of the items as spelt out in QA tools, checklists and GPRs or by the SLR authors. A new name or description was assigned to those items that used similar wording and/or reflected the same constructs. Any apparent discrepancies in wording, spellings and expressions in the items were made consistent. For example, incomplete information, missing data, and the extent of incomplete data are all standard terms that can be described using missing (incomplete) data as a "standardised" nomenclature. Duplicate items were then removed using Microsoft Excel. A single comprehensive list of items used in SLRs or extracted QA tools, checklists, and GPRs was then produced.

Table A.5: List of original items in SLRs included and assigned names by each SLR:

**Author and year**	**Based on NICE/ISPOR tools**	**Original list of items per SLR**	**Assigned name per SLR**
Di Tanna et al. 2021 [1]	Yes	1. Response rates (reported and likelihood to threaten validity)<br>2. Loss of follow up(reported and likelihood to threaten validity)<br>3. Missing data(reported and likelihood to threaten validity)<br>4. Methods to deal with missing data reported<br>5. Country patients match those modelled<br>6. Population characteristics match those modelled<br>7. The use of generic preference e-based instrument (preferably EQ-5D)<br>8. Change in HRQoL undertaken directly from the patients<br>9. Valuation of changes undertaken from general population<br>10. Choice based methods used to value the health states	1. Response rates<br>2. Loss of follow up (Attrition or withdrawals)<br>3. Missing (incomplete) data<br>4. Statistical Analysis/ Data Analysis<br>5. Appropriateness of study country,(setting or facilities)<br>6. Appropriateness of study population<br>7. Appropriate use of generic preference-based method<br>8. Population used to value the health states within the measure<br>9. Population the HSUVs collected from

Author and year	Based on NICE/ISPOR tools	Original list of items per SLR	Assigned name per SLR
Aceituno et al. 2020 [2]	Yes	1. Population characteristics<br>2. Respondent selection and recruitment<br>3. Clearly defined Inclusion/exclusion criteria<br>4. Response rate (%)<br>5. Numbers (%) lost to follow up<br>6. Missing data<br>7. Appropriate use of valuation method<br>8. Appropriate use of generic preference-based method	1. Appropriateness of study population<br>2. Respondent selection and recruitment (Sample selection bias)<br>3. Inclusion and exclusion criteria<br>4. Response rates<br>5. Loss of follow up (Attrition or withdrawals)<br>6. Missing (incomplete) data<br>7. Appropriate use of valuation method<br>8. Appropriate use of generic preference-based method
Bloom et al. 2020 [3]	Yes	1. Respondents<br>2. Valuation Population<br>3. Valuation method<br>4. Inclusion criteria and resulting sample size<br>5. HRQoL response rates<br>6. Missing data and how it was handled<br>7. Loss to follow-up	1. Population used to value the health states within the measure<br>2. Population the HSUVs collected from<br>3. Technique used to value the health states<br>4. Inclusion and exclusion criteria<br>5. Sample size<br>6. Response rates<br>7. Missing (incomplete) data<br>8. Statistical Analysis/ Data Analysis<br>9. Loss of follow up (Attrition or withdrawals)
Cooper, et al. 2020 [4]	Yes	1. The study was conducted in a CKD population (generalizability)<br>2. The study reports original empirical HSU weights<br>3. Data were collected using a generic HRQL measure (i.e. EQ-5D, short-form 6-dimention [SF-6D] (Instrument reporting) -or a mappable equivalent such as short-form 36 [SF-36] or short-form 12 [SF-12]; or the Health Utility Index [HUI]) (Mapping algorithms)<br>4. The study sample size was at least 25 patients (Sample size)<br>5. The study was conducted in a country of interest (i.e., USA, Canada, Australia, China, UK, Spain, Italy, France or Germany) (generalizability and applicability)<br>6. HSU weights were presented in a comprehensive way that is useful to inform cost-effectiveness analysis (e.g. HSU	1. Population the HSUVs collected from<br>2. Data sources/Source of HSUVs<br>3. Technique used to value the health states<br>4. Sample size<br>5. Appropriateness of study country,(setting or facilities)<br>6. Reporting of results<br>7. Respondent selection and recruitment (Sample selection bias)<br>8. Statistical Analysis/ Data Analysis<br>9. Loss of follow up (Attrition or withdrawals)<br>10. Adherence to a predefined study protocol

Author and year	Based on NICE/ISPOR tools	Original list of items per SLR	Assigned name per SLR
		weights were available by CKD stage) (HSUV presentation)<br>**Then methodological bias was assessed based on**:<br>7. Selection bias<br>8. bias in data analysis or interpretation<br>9. Dropouts or missing data<br>10. Bias in study execution	
Golicki et al.2020 [5]	**Yes**	**The following were described for non RCT**<br>1. Sample size<br>2. Response rate<br>3. Measure of variability<br>4. Handling missing data<br>**The following were rated for RCT**<br>5. Randomization(0-2 points)<br>6. Blinding (0-2 points)<br>7. Withdrawals and dropouts (0-1 points)	1. Sample size<br>2. Response rates<br>3. Precision of estimates<br>4. Statistical Analysis/ Data Analysis<br>5. Missing (incomplete) data<br>6. Randomization<br>7. Blinding<br>8. Loss of follow up (Attrition or withdrawals)
Petrou et al. 2020 [6]	**Yes**	**Used all of the domains of the checklist published by Papaioannou and colleagues [18], which generates a maximum score of 8.**<br>1. Sample size<br>2. Respondent selection and recruitment<br>3. Inclusion/exclusion criteria<br>4. Response rates to instrument used to<br>5. Loss to follow-up<br>6. Missing data<br>7. Appropriateness of measure (Measurement instrument)<br>8. Any other problems with the study: Example: Relevance of location (e.g., if patients recruited in non-UK country)	1. Sample size<br>2. Respondent selection and recruitment (Sample selection bias)<br>3. Inclusion and exclusion criteria<br>4. Response rates<br>5. Loss of follow up (Attrition or withdrawals)<br>6. Missing (incomplete) data<br>7. Appropriate use of valuation method<br>8. Appropriate use of generic preference-based method<br>9. Appropriate health state description<br>10. Other sources of bias
Saeed et al. 2020 [7]	Yes	1. Respondent selection and recruitment<br>2. Inclusion/exclusion criteria<br>3. Response rates to instrument used<br>4. Loss to follow-up (if longitudinal measure)<br>5. Missing data<br>6. Appropriateness of measure<br>7. Other problems with the study	1. Respondent selection and recruitment (Sample selection bias)<br>2. Inclusion and exclusion criteria<br>3. Response rates<br>4. Loss of follow up (Attrition or withdrawals)<br>5. Missing (incomplete) data<br>6. Appropriate use of valuation method<br>7. Appropriate use of generic preference-based method<br>8. Appropriate health state description<br>9. Other sources of bias

Author and year	Based on NICE/ISPOR tools	Original list of items per SLR	Assigned name per SLR
Szabo, et al. 2020 [8]	Yes	1. Sample size ≥ 100<br>2. Description of respondent selection and recruitment<br>3. Description of inclusion/exclusion criteria<br>4. Response rate ≥ 60%<br>5. Reporting of attrition/loss to follow-up (for longitudinal studies only)<br>6. Reporting of missingness of data and approaches to deal with it<br>7. Appropriateness of measure (based on the review authors' judgment)	1. Sample size<br>2. Respondent selection and recruitment (Sample selection bias)<br>3. Inclusion and exclusion criteria<br>4. Response rates<br>5. Loss of follow up (Attrition or withdrawals)<br>6. Missing (incomplete) data<br>7. Appropriate use of valuation method<br>8. Appropriate use of generic preference-based method<br>9. Appropriate health state description
Buchanan-Hughes, et al. 2019 [9]	Yes	1. Description of health states<br>2. Sample sizes for different health states<br>3. Reporting of valuation methods used (e.g. time trade-off or standard gamble) to transform the generic EQ-5D responses to HSUVs)	1. Appropriate health state description<br>2. Sample size<br>3. Technique used to value the health states
Li et al.2019 [10]	Yes	1. Sample size<br>2. Respondent selection and recruitment<br>3. Inclusion/exclusion criteria<br>4. Response rates to instrument used to<br>5. Loss to follow-up<br>6. Missing data<br>7. Any other problems with the study: Example: Relevance of location (e.g. if patients recruited in non-UK country)<br>8. Relevancy questions<br>9. Appropriateness of measure (Measurement instrument)<br>10. Time frame	1. Sample size<br>2. Respondent selection and recruitment (Sample selection bias)<br>3. Inclusion and exclusion criteria<br>4. Response rates<br>5. Loss of follow up (Attrition or withdrawals)<br>6. Missing (incomplete) data<br>7. Other sources of bias<br>8. Measure used to describe the HSUVs<br>9. Population the HSUVs collected from<br>10. Population used to value the health states within the measure<br>11. Technique used to value the health states<br>12. Appropriate use of valuation method<br>13. Appropriate use of generic preference-based method<br>14. Appropriate health state description<br>15. Follow up period
Magnus et al. 2019 [11]	Yes	1. Numerical detail of patient recruitment (Recruitment rate)<br>2. Acknowledgement of the non-normality of the distribution of utility values<br>3. Clarity on the treatment of missing values	1. Response Rates<br>2. Non-normality of the distribution of utility values<br>3. Missing (incomplete) data<br>4. Discussions, limitations and conclusions<br>5. Generalizability of findings

Author and year	Based on NICE/ISPOR tools	Original list of items per SLR	Assigned name per SLR
		4. Discussion of the limitations **and,**<br>5. Generalizability of their findings<br>6. Detail on the source of funding<br>7. Source of tariff<br>8. Age adjusted to life expectancy<br>9. Propensity weighting<br>10. Dropouts quantification	6. Source of funding<br>7. Source of tariff<br>8. Indifference search procedure<br>9. Statistical Analysis/ Data Analysis<br>10. Loss of follow up (Attrition or withdrawals)
Paracha et al. 2018 [12]	Yes	1. Sample size<br>2. Measure of variability<br>3. Response rates<br>4. Other quality indictors (e.g., loss of follow up and handling of missing data)	1. Sample size<br>2. Precision of estimates<br>3. Response rates<br>4. Other sources of bias
Meregaglia and Cairns, 2017 [13]	Yes	1. Sample size ≥100<br>2. Description of respondent selection and recruitment<br>3. Description of inclusion/exclusion criteria<br>4. Response rate ≥ 60%<br>5. Reporting of the amount and reasons of loss to follow-up (only for longitudinal studies)<br>6. Reporting of missing data pattern and methods to deal with it<br>**7. Appropriateness of measure (based on the authors' judgment).**<br>8. Any other problems arising from the studies	1. Sample size<br>2. Respondent selection and recruitment (Sample selection bias)<br>3. Inclusion and exclusion criteria<br>4. Response rate<br>5. Loss of follow up (Attrition or withdrawals)<br>6. Missing (incomplete) data<br>7. Appropriate use of valuation method<br>8. Appropriate use of generic preference-based method<br>9. Appropriate health state description<br>10. Other sources of bias
Kua and Davis, 2016 [14]	**Yes**	1. Sample size<br>2. Respondent selection and recruitment<br>3. Inclusion/exclusion criteria<br>4. Response rates to instrument used to<br>5. Lost to follow-up<br>6. Missing data from the instruments<br>7. Any other problems with the study<br>8. Population characteristics<br>9. Population used to measure changes in HRQL<br>10. Population used for valuation of the changes in HRQL<br>11. Technique used to value the health states?<br>12. Instrument is used to describe the health states?	1. Sample size<br>2. Respondent selection and recruitment (Sample selection bias)<br>3. Inclusion and exclusion criteria<br>4. Response rates<br>5. Loss of follow up (Attrition or withdrawals)<br>6. Missing (incomplete) data<br>7. Other sources of bias<br>8. Appropriateness of study population<br>9. Population the HSUVs collected from<br>10. Population used to value the health states within the measure<br>11. Technique used to value the health states?<br>12. Appropriate use of generic preference-based method

**Author and year**	**Based on NICE/ISPOR tools**	**Original list of items per SLR**	**Assigned name per SLR**
Paracha et al. 2016 [15]	Yes	1. Sample size<br>2. Response rates<br>3. Variability around the HSUV to allow quality assessment	1. Sample size<br>2. Response rates<br>3. Precision of estimates
Carter et al. 2015 [16]	Yes	1. Sample size (small, reasonable or sufficient)<br>2. Comparable to advanced GC population<br>3. Response rates (Yes, numbers reported)<br>4. Reporting of loss of follow up<br>5. Reporting of missing data<br>6. Other considerations (health states descriptions, tariff of sources)	1. Sample size<br>2. Appropriateness of study population<br>3. Response rates<br>4. Loss of follow up (Attrition or withdrawals)<br>5. Missing (incomplete) data<br>6. Other sources of bias
Afshari et al.2021 [17]	No	1. Literature Review:<br>2. Research Questions **and** Design:<br>3. Population **and** Sampling<br>¬ Setting<br>¬ target population<br>¬ approach to sampling<br>4. Data Collection **and** Capture<br>¬ Concepts/measures/variabl es<br>¬ Data collection<br>¬ Response or participation rate<br>¬ Completeness of information capture<br>5. Analysis **and** Results Reporting<br>6. Conclusions	1. Literature Review:<br>2. Study aims, objectives and rationale (Research question)<br>3. Study design features /Experimental design<br>4. Appropriateness of study country (setting or facilities)<br>5. Appropriateness of study population<br>6. Respondent selection and recruitment (Sample selection bias)<br>7. Data collection methods/Measurement instrument<br>8. Confounding/Baseline equivalency of groups<br>9. Response rates<br>10. Missing (incomplete) data<br>11. Statistical Analysis/ Data Analysis<br>12. Reporting of results<br>13. Discussions, limitations and conclusions
Carrello et al.2021 [18]	No	1. Indirectness	1. Data sources/Source of HSUVs
Etxeandia-Ikobaltzeta et al. 2020 [19]	No	1. Selection of study population<br>2. Completeness of data<br>3. Selection of measurement instruments<br>4. Administration of measurement instruments<br>5. Presentation of outcomes<br>6. Understanding of participants<br>2. Data analysis	1. Respondent selection<br>2. and recruitment Missing (incomplete) data<br>3. Response rate<br>4. Data collection methods/Measurement instrument<br>5. Administration procedure<br>6. Appropriate health state description<br>7. Understanding of participants<br>2. Statistical Analysis/ Data Analysis

Author and year	Based on NICE/ISPOR tools	Original list of items per SLR	Assigned name per SLR
Han et al. 2021 [20]	No	1. Systematic review<br>2. Sensitivity analysis<br>3. Elicitation method<br>4. Responder<br>5. Population comparability<br>6. Health states comparability<br>7. Discussion on proper use	1. Data sources/Source of HSUVs<br>2. Sensitivity analysis<br>3. Technique used to value the health state<br>4. Population the HSUVs collected from<br>5. Population used to value the health states within the measure<br>6. Appropriateness of study country (setting or facilities)<br>7. Appropriate health state description<br>8. Discussions, limitations and conclusions
Haridoss et al. 2021 [21]	**No**	**Case control studies (NOS for Case studies)**<br>1. Case definition adequacy<br>2. Representativeness of cases<br>3. Selection of controls<br>4. Definition of controls<br>5. Comparability of cases and controls<br>6. Ascertainment of exposure<br>7. Same method of ascertainment of cases and controls<br>8. Non response rate<br>**Cohort studies (NOS for Cohort studies)**<br>9. Representative of exposed cohorts<br>10. Selection of non-exposed cohorts<br>11. Demonstration that outcome of interest was not present at the start<br>12. Assessment of outcome<br>13. Length of follow up period<br>14. Adequacy of follow up of cohorts<br>**CS studies (AXIS Tool)**<br>15. Study aims and or objectives<br>16. Study design<br>17. Sample size<br>18. Target or reference population<br>19. Appropriate sample frame<br>20. Measures to address non-responders<br>21. Appropriateness of risk factors and outcome variables<br>22. Determination of statistical significance<br>23. Methods and statistical methods sufficiency<br>24. Description of basic data<br>25. Respond rate (bias)<br>26. Internal consistency of results	1. Case definition<br>2. Representativeness of cases<br>3. Selection of controls<br>4. Control and contemporary groups<br>5. Confounding/Baseline equivalency of groups<br>6. Ascertainment of exposure<br>7. Ascertainment of cases and controls<br>8. Response rates<br>9. Representative of exposed cohorts<br>10. Selection of non-exposed cohorts<br>11. Absence of outcome of interest at index date<br>12. Assessment of outcome<br>13. Follow up period<br>14. Adequacy of follow up of cohorts<br>15. Study aims, objectives and rationale (Research question)<br>16. Study design features /Experimental design<br>17. Sample size<br>18. Appropriateness of study population<br>19. Respondent selection and recruitment (Sample selection bias)<br>20. Statistical Analysis/ Data Analysis<br>21. Confounding/Baseline equivalency of groups<br>22. Statistical Analysis/ Data Analysis<br>23. Statistical Analysis/ Data Analysis<br>24. Description of basic data<br>25. Response rates<br>26. Internal consistency of results<br>27. Reporting bias

Author and year	Based on NICE/ISPOR tools	Original list of items per SLR	Assigned name per SLR
		27. Reporting of all results<br>28. Discussions, limitations and conclusions<br>29. Limitations of studies<br>30. Sources of funding<br>31. Ethical approval	28. Discussions, limitations and conclusions<br>29. Discussions, limitations and conclusions<br>30. Sources of funding<br>31. Ethical approval
Jiang et al. 2021 [22]	No	1. Representativeness of the sample<br>2. Sample size<br>3. Non-respondents<br>4. Ascertainment of the exposure<br>5. Comparability<br>6. Assessment of the outcome<br>7. Statistical test<br>8. Case definition adequate?<br>9. Selection of Controls<br>10. Definition of Controls<br>11. Representativeness of the cases<br>12. Same method of ascertainment for cases and controls<br>13. Ascertainment of exposure<br>14. Non-Response rate	1. Appropriateness of study population<br>2. Sample size<br>3. Response rates<br>4. Ascertainment of the exposure<br>5. Confounding/Baseline equivalency of groups<br>6. Assessment of the outcome<br>7. Statistical Analysis/ Data Analysis<br>8. Case definition<br>9. Selection of Controls<br>10. Control and contemporary groups<br>11. Representativeness of the cases<br>12. Ascertainment of cases and controls<br>13. Reponses rates
Park et al. 2021 [23]	No	RoBANS evaluated the following:<br>1. Selection of participants<br>2. Confounding variables<br>3. Intervention measurement<br>4. Blinding of outcome assessment<br>5. Incomplete outcome data<br>6. Selective outcome reporting<br>The NOS evaluated the following<br>7. Representativeness of the exposed cohort<br>8. Selection of the non-exposed cohort<br>9. Ascertainment of exposure<br>10. Absence of outcome of interest at the start of the study<br>11. Comparability of cohorts based on the design<br>12. Assessment of outcome<br>13. Enough follow-up for outcomes to occur<br>14. Non-response rate	1. Respondent selection and recruitment (Sample selection bias)<br>2. Confounding/Baseline equivalency of groups<br>3. Intervention measurement<br>4. Blinding<br>5. Missing (incomplete) data<br>6. Reporting bias<br>7. Appropriateness of study population<br>8. Selection of the non-exposed cohort<br>9. Ascertainment of exposure<br>10. Absence of outcome of interest at index date<br>11. Confounding/Baseline equivalency of groups<br>12. Assessment of outcome<br>13. Follow up period<br>14. Response rates
Landeiro et al. 2020 [24]	No	1. Selection bias<br>2. Study Design<br>3. Confounders<br>4. Blinding<br>5. Data collection methods<br>6. Withdrawals and dropouts	1. Respondent selection and recruitment (Sample selection bias)<br>2. Study design features /Experimental design<br>3. Confounding/Baseline equivalency of groups<br>4. Blinding

Author and year	Based on NICE/ISPOR tools	Original list of items per SLR	Assigned name per SLR
			5. Data collection methods/Measurement instrument<br>6. Loss of follow up (Attrition or withdrawals)
Rebchuk et al. 2020 [25]	No	1. Inclusion and exclusion criteria clearly described?<br>2. Randomly selected sample or community-dwelling?<br>3. Rationale for removing outlier data reported?<br>4. mRS assessors blinded to the status of the patient?<br>5. Multiple investigators assess mRS outcomes?<br>6. Structured mRS questionnaire?<br>7. Comorbidities reported?<br>8. Time post-stroke reported?<br>9. Proxy rates by mRS level?	1. Inclusion and exclusion criteria<br>2. Respondent selection and recruitment (Sample selection bias)<br>3. Statistical Analysis/ Data Analysis<br>4. Blinding<br>5. Assessment of outcome<br>6. Data collection methods/Measurement instrument<br>7. Confounding/Baseline equivalency of groups<br>8. Time of assessment<br>9. Proxy rates by mRS level?
Yang et al. 2020 [26]	No	1. Missing data	1. Missing (incomplete) data
Yuan et al. 2020 [27]	**No**	**Case control studies (NOS for Case studies)**<br>1. Case definition adequacy<br>2. Representativeness of the exposed cohort<br>3. Selection of controls<br>4. Definition of controls<br>5. Comparability of cases and controls<br>6. Ascertainment of exposure<br>7. Same method of ascertainment of cases and controls<br>8. Non response rate<br>**Cohort studies (NOS for Cohort studies)**<br>9. Representative of exposed cohorts<br>10. Selection of non-exposed cohorts<br>11. Demonstration that outcome of interest was not present at the start<br>12. Assessment of outcome<br>13. Length of follow up period<br>**14.** Adequacy of follow up of cohorts	1. Case definition<br>2. Representativeness of cases<br>3. Selection of controls<br>4. Control and contemporary groups<br>5. Confounding/Baseline equivalency of groups<br>6. Ascertainment of exposure<br>7. Ascertainment of cases and controls<br>8. Response rates<br>9. Representative of exposed cohorts<br>10. Selection of non-exposed cohorts<br>11. Absence of outcome of interest at index date<br>12. Assessment of outcome<br>13. Follow up period<br>14. Adequacy of follow up of cohorts
Foster et al. 2019 [28]	**No**	**Reporting Items**<br>1. The hypothesis / aim / objective<br>2. Characteristics of the patients included<br>3. Interventions of interest<br>4. Principal confounders in each group<br>5. Findings of the study	1. Study aims, objectives and rationale (Research question)<br>2. Inclusion and exclusion criteria<br>3. Control and contemporary groups<br>4. Confounding/Baseline equivalency of groups<br>5. Reporting of results

Author and year	Based on NICE/ISPOR tools	Original list of items per SLR	Assigned name per SLR
		6. Estimates of the random variability<br>7. Important adverse events<br>8. Lost to follow-up<br>9. Actual probability values<br>**External validity (3 items) – the ability to generalize findings of the study**;<br>10. Representativeness of study subjects<br>11. Representativeness of staff, settings and facilities<br>**Study bias (7 items) – to assess bias in the intervention and outcome measure(s);**<br>12. Blinding of subjects<br>13. Blinding of outcome accessors<br>14. Data dredging<br>15. Adjusting for different length of follow up<br>16. Statistical tests used for main outcomes<br>17. Compliance with the intervention/s<br>18. Main outcome measured used accurate (valid and reliable)?<br>**Confounding and selection bias (6 items) – to determine bias from sampling or group assignment;**<br>19. Baseline characteristics<br>20. Recruited over the same period of time?<br>21. Randomization<br>22. Concealment of allocation<br>23. Adequate adjustment for confounding in<br>24. Loss of follow up accounted for<br>**Power of the study (1 items) – to determine if findings are due to chance."**<br>25. Sample size	6. Precision of estimates<br>7. Important adverse events<br>8. Loss of follow up (Attrition or withdrawals)<br>9. P value reporting<br>10. Appropriateness of study population<br>11. Appropriateness of study country,(setting or facilities)<br>12. Blinding<br>13. Blinding<br>14. Reporting bias<br>15. Statistical Analysis/ Data Analysis<br>16. Statistical Analysis/ Data Analysis<br>17. Adherence to a predefined study protocol<br>18. Confounding/Baseline equivalency of groups<br>19. Confounding/Baseline equivalency of groups<br>20. Randomization<br>21. Respondent selection and recruitment (Sample selection bias)<br>22. Statistical Analysis/ Data Analysis<br>23. Statistical Analysis/ Data Analysis<br>24. Sample size
Hatswell et al. 2019 [29]	**No**	The article did not provide sufficient information to understand how the QA was conducted.	
Khadka et al. 2019 [30]	No	1. Bibliographic details, including year of publication<br>2. Country/ geographical jurisdiction<br>3. Setting (hospital inpatient ward, hospital outpatient clinic, general practice, school, via post, via internet, other)<br>4. Health descriptor(s), which could take the form of a health condition/disease, health state, or intervention descriptor	1. Bibliographic details, including year of publication<br>2. Appropriateness of study country,(setting or facilities)<br>3. Respondent selection and recruitment (Sample selection bias)<br>4. Appropriate health state description<br>5. Population used to value the health states within the measure

Author and year	Based on NICE/ISPOR tools	Original list of items per SLR	Assigned name per SLR
		5. Respondent type (self-assessment by children, proxy assessment by parents, caregivers, nurses, physicians, other proxies)<br>6. Age of target childhood group (reported as age at diagnosis, age at study, and associated descriptive statistics)<br>7. Size of study population<br>8. Direct valuation method applied (if applicable)<br>9. Indirect valuation method applied (if applicable)<br>10. Utility tariff if indirect valuation method was applied<br>11. Utility or VAS scores (including central statistics and measures of variability)<br>12. Study design (cross-sectional study, clinical trial, prospective observational, internet survey, other)<br>13. Response quality (response rate, information on dropouts, reasons for loss to follow-up, etc.)<br>14. Statistical method for analyzing utilities; and<br>15. Any reported methodological concerns.<br><br>**A point for each of these items if reported.**	6. Confounding/Baseline equivalency of groups<br>7. Sample size<br>8. Appropriate use of valuation method<br>9. Appropriate use of generic preference-based method<br>10. Source of tariff (value set)<br>11. Reporting of results<br>12. Study design features /Experimental design<br>13. Response Rates<br>14. Loss of follow up (Attrition or withdrawals)<br>15. Statistical Analysis/ Data Analysis<br>16. Other sources of bias
Van Wilder et al. 2019 [31]	No	1. Study aim and objectives<br>2. Study design<br>3. Subject group selection<br>4. Subject and comparison group<br>5. Outcome measures<br>6. Sample size<br>7. Statistical analysis<br>8. Estimate of variance<br>9. Results reported<br>10. Confounding<br>11. Conclusions	1. Study aims, objectives and rationale (Research question)<br>2. Study design features /Experimental design<br>3. Respondent selection and recruitment (Sample selection bias)<br>4. Control and contemporary groups<br>5. Assessment of outcome<br>6. Sample size<br>7. Statistical Analysis/ Data Analysis<br>8. Precision of estimates<br>9. Reporting of results<br>10. Confounding/Baseline equivalency of groups<br>11. Discussions, limitations and conclusions
Kwon et al. 2018 [32]	No	1. Bibliographic details, including year of publication	1. Bibliographic details, including year of publication

Author and year	Based on NICE/ISPOR tools	Original list of items per SLR	Assigned name per SLR
		2. Country/ geographical jurisdiction<br>3. Setting (hospital inpatient ward, hospital outpatient clinic, general practice, school, via post, via internet, other)<br>4. Health descriptor(s), which could take the form of a health condition/disease, health state, or intervention descriptor<br>5. Respondent type (self-assessment by children, proxy assessment by parents, caregivers, nurses, physicians, other proxies)<br>6. Age of target childhood group (reported as age at diagnosis, age at study, and associated descriptive statistics)<br>7. Size of study population<br>8. Direct valuation method applied (if applicable)<br>9. Indirect valuation method applied (if applicable)<br>10. Utility tariff if indirect valuation method was applied<br>11. Utility or VAS scores (including central statistics and measures of variability)<br>12. Study design (cross-sectional study, clinical trial, prospective observational, internet survey, other<br>13. Response quality (response rate,<br>14. Information on dropouts, reasons for loss to follow-up, etc.)<br>15. Statistical method for analyzing utilities; and<br>16. **Any reported methodological concerns.**<br><br>**A point for each of these items** <u>if reported</u>**.**	2. Appropriateness of study country,(setting or facilities)<br>3. Respondent selection and recruitment (Sample selection bias)<br>4. Appropriate health state description<br>5. Population used to value the health states within the measure<br>6. Confounding/Baseline equivalency of groups<br>7. Sample size<br>8. Appropriate use of valuation method<br>9. Appropriate use of generic preference-based method<br>10. Source of tariff (value set)<br>11. Reporting of results<br>12. Study design features /Experimental design<br>13. Response Rates<br>14. Loss of follow up (Attrition or withdrawals)<br>15. Statistical Analysis/ Data Analysis<br>16. Other sources of bias
Tran et al. 2018 [33]	No	1. Missing data	1. Missing (incomplete) data
Ó Céilleachair et al. 2017 [34]	No	1. HRQoL instruments employed<br>2. Sample size<br>3. Use of comparison groups,<br>4. Elicitation group<br>5. Clarity of analysis<br>6. Clearly stated research aims<br>7. Appropriate use of population norms<br>8. Discussion of limitations	1. Technique used to value the health states<br>2. Sample size<br>3. Control and contemporary groups<br>4. Population the HSUVs collected from<br>5. Statistical Analysis/ Data Analysis

Author and year	Based on NICE/ISPOR tools	Original list of items per SLR	Assigned name per SLR
			6. Study aims, objectives and rationale (Research question)<br>7. Source Of Tariff<br>8. Discussions, limitations and conclusions
Ward Fuller et al. 2017 [35]	No	1. Response rates<br>2. Loss to follow up<br>3. Missing data<br>4. Content validity<br>5. Face validity<br>6. Construct validity<br>7. Responsiveness<br>8. Reliability<br>9. Choice v feeling based valuation<br>10. Credible extrapolation of health state valuations?<br>11. Empirical validity of valuation method (against revealed, stated or hypothesized preferences)<br>12. Other sources of bias	1. Response rates<br>2. Loss of follow up (Attrition or withdrawals)<br>3. Missing (incomplete) data<br>4. Instrument validity (Face, Content and Construct)<br>5. Instrument responsiveness<br>6. Instrument reliability<br>7. Technique used to value the health states<br>8. Credible extrapolation of health state valuations?<br>9. Appropriate use of generic preference-based method<br>10. Other sources of bias
Zrubka et al. 2017 [36]	No	1. Selection (sequence generation/ allocation concealment)<br>2. Performance (blinding of participants and personnel)<br>3. Detection (blinding of outcome assessment)<br>4. Attrition (incomplete outcome data)<br>5. Reporting bias<br>6. Other bias<br>7. Mode of administration<br>8. EQ5D respondent (%)<br>9. Version of EQ-5D descriptive system<br>10. Reporting of outcomes<br>11. Value-set used	1. Respondent selection and recruitment (Sample selection bias)<br>2. Performance (blinding of participants and personnel),<br>3. Blinding<br>4. Loss of follow up (Attrition or withdrawals)<br>5. Reporting bias<br>6. Other sources of bias<br>7. Administration procedure<br>8. Response rates<br>9. Instrument version<br>10. Reporting of results<br>11. Source of tariff (value set)
Brennan et al. 2015 [37]	No	1. Study sample was representative of the T2DM population<br>2. A valid instrument was used<br>3. A valid scoring algorithm was used<br>4. Utility estimates were controlled for confounding by other factors known to be associated with utility.	1. Appropriateness of study population<br>2. Data collection methods/Measurement instrument<br>3. Scoring algorithm<br>4. Confounding/Baseline equivalency of groups<br>5. Response rates
Eiring et al. 2015 [38]		**A. <u>External validity of the study</u>**<br>1. Patient population<br>2. Sample size<br>3. recruitment procedures (generalisability)<br>4. Randomization and/or stratification	1. Appropriateness of study population<br>2. Sample size<br>3. Respondent selection and recruitment (Sample selection bias)<br>4. Randomization

Author and year	Based on NICE/ISPOR tools	Original list of items per SLR	Assigned name per SLR
		5. Completion rates<br>6. Decision problem<br>**B. <u>Construct representation</u>**<br>7. Relevance of options, attributes and attribute levels<br>8. sources used in the construction of options, attributes and attribute levels<br>9. Level of detail and accuracy of options attributes and attribute levels<br>**C. <u>Construct-irrelevant variance</u>**<br>10. Piloting and pre testing of the study<br>11. Question framing<br>12. Evidence of interviewer influence<br>13. Skills, emotions and cognitive ability of participants<br>14. Cognitive burden<br>15. Scaling methods<br>16. Indifference procedure<br>**D. <u>Reporting and analysis?</u>**<br>17. Completeness of the outcome data<br>18. Distribution of data (skewed or normally distributed)<br>19. Statistical techniques<br>20. Reporting of uncertainty of estimates<br>21. Heterogeneity and patient subgroups analysis<br>**E. <u>Other aspects</u>**<br>22. Tests of internal validity<br>23. Answerable research questions<br>24. Comparability with other studies<br>25. Protocol or supplementary information<br>26. limitations and weaknesses of the study	5. Missing (incomplete) data<br>6. Study aims, objectives and rationale (Research question)<br>7. Attributes and levels<br>8. Attributes and levels<br>9. Attributes and levels<br>10. Appropriate health state description<br>11. Data collection methods/Measurement instrument<br>12. Administration procedure<br>13. Appropriate health state description<br>14. Appropriate health state description<br>15. Piloting and pre testing of the study<br>16. Question framing<br>17. Administration procedure<br>18. Understanding of participants<br>19. Instrument acceptability (level of detail, comprehensibility vs burden vs cognitive overload)<br>20. Technique used to value the health states<br>21. and indifference procedure<br>22. Reporting bias<br>23. Non-normality of the distribution of utility values<br>24. Statistical Analysis/ Data Analysis<br>25. Reporting of results<br>26. Statistical Analysis/ Data Analysis<br>27. Tests of internal validity<br>28. Study aims, objectives and rationale (Research question)<br>29. Discussions, limitations and conclusions<br>30. Availability and accessibility of a study protocol<br>31. Discussions, limitations and conclusions
Gheorghe et al. 2015 [39]	No	1. Data sources/Source of HSUVs	1. Data sources/Source of HSUVs<br>2.
Li et al. 2015 [40]	**No**	**MINOR Scale for NRS(reporting)**<br>1. Clearly stated aim<br>2. Inclusion of consecutive patients<br>3. Prospective collection of data	1. Study aims, objectives and rationale (Research question)<br>2. Inclusion and exclusion criteria<br>3. Study design features /Experimental design

Author and year	Based on NICE/ISPOR tools	Original list of items per SLR	Assigned name per SLR
		4. End points appropriate<br>5. Unbiased assessment of the study end point<br>6. Follow-up period appropriate to the aim of the study<br>7. Loss to follow-up < 5%<br>8. Prospective calculation of the study size<br>9. An adequate control group<br>10. Contemporary groups<br>11. Baseline equivalence of groups<br>12. Adequate statistical analysis<br>**Jadad scale for RCT(reporting)**<br>13. Randomization<br>**14.** Blinding<br>**15.** Withdrawals and dropouts	4. Appropriateness of endpoints<br>5. Assessment of outcome<br>6. Follow up period<br>7. Loss of follow up (Attrition or withdrawals)<br>8. Sample size<br>9. Control and contemporary groups<br>10. Confounding/Baseline equivalency of groups<br>11. Statistical Analysis/ Data Analysis<br>12. Randomization<br>13. Blinding<br>14. Loss of follow up (Attrition or withdrawals)

Table A.6: List of original items and assigned names per each tool and GPR

Author and year	NICE/ISPOR tools	Original list of items per tool or GPR	Assigned name per each item of a tool or GPR
Brazier et al.2019 [41]	**Yes**	**Methodological flaws:**<br>1. Precision of the evidence:<br>¬ Variance in HSUV and<br>¬ Sample size<br>2. Response rates<br>3. Loss of follow up<br>4. Missing (incomplete) data<br>**Data applicability**<br>5. Study population matching modelled population<br>6. Health states matching the modelled health states<br>7. Timing of data collection<br>8. Validity and sensitivity of instrument<br>9. Measure used to describe the HSUVs<br>10. Population used to value the health states within the measure<br>11. Technique used to value the health state	1. Sample size<br>2. Precision of estimates<br>3. Response rates<br>4. Loss of follow up (Attrition or withdrawals)<br>5. Missing (incomplete) data<br>6. Appropriateness of study population<br>7. Appropriate health state description<br>8. Time of assessment<br>9. Instrument validity (Face, Content and Construct)<br>10. Sensitivity of instrument<br>11. Measure used to describe the HSUVs<br>12. Technique used to value the health states<br>13. Appropriate health state description<br>14. Population used to value the health states within the measure)<br>15. Population the HSUVs collected from
Ara et al.2017 [42]	Yes	5 domains or components as recommended by ISPOR are described and elaborated:<br>**1. Relevance to the decision problem**<br>¬ Population characteristics<br>¬ Respondent selection and recruitment<br>-Inclusion and exclusion criteria<br>**2. Free from known sources of bias**<br>¬ Sample size<br>¬ Response rates<br>¬ Loss to follow up<br>¬ Missing (incomplete) data<br>**3. Measured using a validated method**<br>¬ Appropriate use of valuation method<br>¬ Appropriate use of generic preference-based method<br>Appropriate health state description<br>**4. Appropriate to the condition and population of interest and the perspective of the decision maker**<br>5. **In line with reimbursement agency** requirements<br>-Measure used to describe the HSUVs	1. Sample size<br>2. Respondent selection and recruitment (Sample selection bias)<br>3. Inclusion and exclusion criteria<br>4. Response rates<br>5. Loss of follow up (Attrition or withdrawals)<br>6. Missing (incomplete) data<br>7. Statistical Analysis/ Data Analysis<br>8. Appropriate use of valuation method<br>9. Other sources of bias<br>10. Appropriateness of study population<br>11. Measure used to describe the HSUVs<br>12. Population used to value the health states within the measure

Author and year	NICE/ISPOR tools	Original list of items per tool or GPR	Assigned name per each item of a tool or GPR
		-Population used to value the health states within the measure<br>-Technique used to value the health state	13. Technique used to value the health state<br>14. Appropriate use of generic preference-based method
Papaioannou et al. 2010 [43] and 2013 [44]	Yes	1. Sample size<br>2. Respondent selection and recruitment<br>3. Inclusion/exclusion criteria<br>4. Response rates to instrument used to<br>5. Lost to follow-up<br>6. Missing (incomplete) data from the instruments **and** handling of it<br>7. Any other problems with the study<br>8. Population characteristics<br>9. Population used to measure changes in HRQL<br>10. Population used for valuation of the changes in HRQL<br>11. Technique used to value the health states?<br>12. Instrument is used to describe the health states?	1. Sample size<br>2. Respondent selection and recruitment (Sample selection bias)<br>3. Inclusion and exclusion criteria<br>4. Response rates<br>5. Loss of follow up (Attrition or withdrawals)<br>6. Missing (incomplete) data<br>7. Statistical Analysis/ Data Analysis<br>8. Other sources of bias<br>9. Appropriateness of study population<br>10. Population the HSUVs collected from<br>11. Population used to value the health states within the measure<br>12. Technique used to value the health states?<br>13. Appropriate use of generic preference-based method
Sterne et al. 2019 [45]	No	1. Bias arising from the randomization process<br>2. Bias due to deviations from intended interventions<br>3. Bias due to missing outcome data<br>4. Bias due to measurement of outcome<br>5. Bias due to selection of reported results<br>Note the RoB 2 do not classify the RoB by stage of trial.	1. Randomization<br>2. Allocation sequence concealment<br>3. Confounding/Baseline equivalency of groups<br>4. Adherence to a predefined study protocol<br>5. Blinding<br>6. Missing (incomplete) data<br>7. Assessment of outcome<br>8. Statistical Analysis/ Data Analysis<br>9. Reporting bias
Xie et al 2019 [46]	**No**	**Methodological issues**<br>1. Sources of preferences<br>2. Scaling methods<br>3. Use of anchors<br>4. Measurement properties (reliability and validity)	1. Source of utility values<br>2. Technique used to value the health states<br>3. Description and use of anchor states<br>4. Instrument reliability

Author and year	NICE/ISPOR tools	Original list of items per tool or GPR	Assigned name per each item of a tool or GPR
		**Applicability**<br>5. Target populations<br>6. Health states<br>7. Preference elicitation methods<br>8. Modeling techniques	5. Instrument validity (Face, Content and Construct)<br>6. Appropriateness of study population<br>7. Appropriate health state description<br>8. Technique used to value the health states<br>9. Modeling techniques
Zhang et al 2019 [47]	No	**A. <u>Risk of bias</u>**<br>1. Selection of participants into the study<br>2. Completeness of data<br>3. Measurement instrument<br>¬ Validity and reliability<br>¬ Administered appropriately<br>¬ Representation of health states<br>¬ Understanding of instrument<br>4. Data analysis<br>**B. <u>Indirectness</u>**<br>5. Indirectness due to PICO elements<br>¬ Population characteristics<br>¬ Intervention and comparators<br>¬ Outcomes assessed<br>6. Indirectness due to methodological elements<br>¬ Direct vs Indirect methodologies<br>¬ Direct elicitation vs mapping studies	1. Respondent selection and recruitment<br>2. Missing (incomplete) data<br>3. Response rates<br>4. Data collection methods/Measurement instrument<br>5. Administration procedure<br>6. Appropriate health state description<br>7. Understanding of participants<br>8. Statistical Analysis/ Data Analysis<br>9. Intervention and comparators<br>10. Outcomes assessed<br>11. Appropriate use of generic preference-based method<br>10. Appropriate use of valuation method
Petrou et al. 2018 [48]	No	1. Face validity<br>2. Empirical validity<br>3. Preferred utility measure<br>4. Preferred population group	1. Instrument validity (Face, Content and Construct)<br>2. Technique used to value the health states<br>3. Appropriateness of study population
Wailoo et al.2017 [49]	**No**	**Items for reporting of mapping studies**<br>1. Data set<br>2. Study design<br>3. Patient sampling<br>4. Baseline and follow-up characteristics up characteristics<br>5. Missing data<br>6. Response rates<br>7. Statistical model and justification<br>8. Covariates used<br>9. Model Selection and Performance	1. Data sets<br>2. Study design features /Experimental design<br>3. Respondent selection and recruitment (Sample selection bias)<br>4. Confounding/Baseline equivalency of groups<br>5. Missing (incomplete) data<br>6. Response rates<br>7. Statistical Analysis/ Data Analysis<br>8. Confounding/Baseline equivalency of groups

Author and year	NICE/ISPOR tools	Original list of items per tool or GPR	Assigned name per each item of a tool or GPR
		10. Coefficient values<br>11. Uncertainty in the estimated coefficients	9. Model Selection and Performance<br>10. Reporting of results<br>11. Reporting of results
Downes et al.2016 [50]	No	1. Study aims and or objectives<br>2. Study design<br>3. Sample size<br>4. Target or reference population<br>5. Appropriate sample frame<br>6. Selection process<br>7. Measures to address non-responders<br>8. Appropriateness of risk factors and outcome variables<br>9. Determination of statistical significance<br>10. Methods and statistical methods sufficiency<br>11. Description of basic data<br>12. Respond rate (bias)<br>13. Internal consistency of results<br>14. Reporting of all results<br>15. Discussions, limitations and conclusions<br>16. Limitations of studies<br>17. Sources of funding<br>18. Ethical approval	1. Study aims, objectives and rationale (Research question)<br>2. Study design features /Experimental design<br>3. Sample size<br>4. Appropriateness of study population<br>5. Respondent selection and recruitment (Sample selection bias)<br>6. Statistical Analysis/ Data Analysis<br>7. Confounding/Baseline equivalency of groups<br>8. Statistical Analysis/ Data Analysis<br>9. Statistical Analysis/ Data Analysis<br>10. Description of basic data<br>11. Response rates<br>12. Internal consistency of results<br>13. Reporting of all results<br>14. Discussions, limitations and conclusions<br>15. Discussions, limitations and conclusions<br>16. Sources of funding<br>17. Ethical approval
Gupta et al. 2016 [51]	No	1. Selection of participants<br>2. Randomization<br>3. Inclusion and exclusion criteria<br>4. Study objectives<br>5. Study design<br>6. Blinding of investigators<br>7. Assessment of exposure<br>8. Misclassification bias<br>9. Losses to follow up<br>10. Confounding<br>11. Reporting of results	1. Respondent selection and recruitment (Sample selection bias)<br>2. Randomization<br>3. Inclusion and exclusion criteria<br>4. Study aims, objectives and rationale (Research question)<br>5. Study design features /Experimental design<br>6. Blinding<br>7. Ascertainment of exposure<br>8. Misclassification bias<br>9. Loss of follow up (Attrition or withdrawals)<br>10. Confounding/Baseline equivalency of groups<br>11. Reporting of results
O'Connor et al. 2016 [52]	No	No quality appraisal was done in the SLR. Yet it is cited as a source of inspiration for developing an ad-hoc QA tool by A, Ó. C., et al.2015	No quality appraisal was done in the SLR. Yet it is cited as a source of inspiration for developing an ad-hoc QA tool by A, Ó. C., et al.2015
Sterne et al. 2016 [53]	No	Groups A: Pre intervention domains<br>2. Bias due to confounding	2. Confounding/Baseline equivalency of groups

Author and year	NICE/ISPOR tools	Original list of items per tool or GPR	Assigned name per each item of a tool or GPR
		3. Bias due to selection participants into study<br>**Group B: During the intervention domains**<br>4. Bias in classification of intervention<br>**Group C: Post intervention bias**<br>5. Bias due to deviation from intended intervention (often referred to as performance bias in Cochrane)<br>6. Bias due to Missing (incomplete) data<br>7. Bias due to measurement of outcome<br>8. Bias is selection of reported results"	3. Respondent selection and recruitment (Sample selection bias)<br>4. Misclassification bias<br>5. Adherence to a predefined study protocol<br>6. Missing (incomplete) data<br>7. Assessment of outcome<br>8. Reporting bias
Gupta et al. 2015 [54]	No	1. Risk of outcome ascertainment bias<br>¬ Blinded<br>2. Risk of confounding bias<br>¬ Collected and analyzed<br>3. Completeness of follow-up data	1. Assessment of outcome<br>2. Blinding<br>3. Confounding/Baseline equivalency of groups<br>4. Missing (incomplete) data
Wolowacz et al.2015 [55]	No	1. Choice of instrument<br>2. Timing and frequency of assessments and period of follow-up<br>3. Collection of covariates at baseline and every stage of HSUV assessment<br>4. Choice of respondents<br>5. Mode of administration<br>6. Methods to address any heterogeneity of the patient sample or issues with generalizability of results	1. Data collection methods/Measurement instrument<br>2. Time of assessment<br>3. Follow up period<br>4. Confounding/Baseline equivalency of groups<br>5. Population used to value the health states within the measure<br>6. Administration procedure<br>7. Statistical Analysis/ Data Analysis
Calvert et al. 2013 [56]	No	1. Title and abstract<br>2. Background and objectives<br>3. Trial design<br>4. Participants<br>5. Interventions<br>6. Outcomes<br>7. Sample size<br>8. Sequence generation<br>9. Allocation concealment mechanism<br>10. Implementation<br>11. Blinding<br>12. Statistical methods<br>13. Participant flow (a diagram is strongly recommended)<br>14. Recruitment<br>15. Baseline data<br>16. Numbers analyzed<br>17. Outcomes and estimation<br>18. Ancillary analyses	3. Study aims, objectives and rationale (Research question)<br>4. Study design features /Experimental design<br>5. Inclusion and exclusion criteria<br>6. Appropriateness of study country,(setting or facilities)<br>7. Interventions ,control and contemporary groups<br>8. Appropriateness of endpoints<br>9. Sample size<br>10. Respondent selection and recruitment (Sample selection bias)<br>11. Respondent selection and recruitment (Sample selection bias)<br>12. Response rates<br>13. Blinding

Author and year	NICE/ISPOR tools	Original list of items per tool or GPR	Assigned name per each item of a tool or GPR
		19. Harms<br>20. Limitations<br>21. Generalizability<br>22. Interpretation<br>23. Registration<br>24. Protocol<br>25. Funding	14. Statistical Analysis/ Data Analysis<br>15. Study design features /Experimental design<br>16. Recruitment period<br>17. Follow up period<br>18. Confounding/Baseline equivalency of groups<br>19. Sample size<br>20. Assessment of outcome<br>21. Statistical Analysis/ Data Analysis<br>22. Important adverse events<br>23. Generalizability of findings<br>24. Discussions, limitations and conclusions<br>25. Availability and accessibility of a study protocol<br>26. Sources of funding
Herzog et al. 2013 [57]	**No**	**Adapted from NOS**<br>1. Representativeness sample<br>2. Ascertainment of exposure<br>3. Non response rates<br>4. Sample size<br>5. Comparability<br>6. Design and analysis<br>7. Assessment of outcome<br>8. Assessment of outcome	1. Appropriateness of study population<br>2. Ascertainment of exposure<br>3. Response rates<br>4. Sample size<br>5. Confounding/Baseline equivalency of groups<br>6. Study design features /Experimental design<br>7. Statistical Analysis/ Data Analysis<br>8. Assessment of outcome
Braizer and Rowen, 2011 [58]	No	Focus on instrument's **appropriateness** to the decision maker (NICE) and considers issues to do with:<br>1. Validity<br>¬ Content<br>¬ Construct<br>¬ Face<br>2. Responsiveness:<br>3. Practicality: acceptability to respondents<br>4. Reliability:	1. Instrument validity (Face, Content and Construct)<br>2. Instrument responsiveness<br>3. Instrument acceptability (level of detail, comprehensibility vs burden vs cognitive overload)<br>4. Instrument reliability
Higgins et al. 2011 [59]	No	27. Selection bias<br>¬ Sequence generation<br>¬ Allocation concealment<br>28. Performance bias<br>¬ Blinding of participants and personnel<br>29. Detection biais<br>¬ Blinding of outcome assessment<br>30. Attrition bias<br>¬ Missing (incomplete) data/incomplete outcomes data	6. Respondent selection and recruitment (Sample selection bias)<br>7. Blinding<br>8. Blinding<br>9. Loss of follow up (Attrition or withdrawals)<br>10. Missing (incomplete) data<br>11. Reporting bias<br>12. Other sources of bias

Author and year	NICE/ISPOR tools	Original list of items per tool or GPR	Assigned name per each item of a tool or GPR
		31. Reporting bias<br>¬ Selective reporting<br>32. Other bias"	
Park et al. 2011 [60]	No	1. Selection of participants<br>2. Confounding variables<br>3. Measurement of exposure<br>4. Blinding of outcome assessments- Detection bias<br>5. Incomplete outcome data<br>6. Selective outcome reporting	1. Respondent selection and recruitment (Sample selection bias)<br>2. Confounding/Baseline equivalency of groups<br>3. Ascertainment of exposure<br>4. Blinding<br>5. Missing (incomplete) data<br>6. Reporting bias
Accent and RAND Europe 2010 [61]	No	1. Population sampling<br>2. Sample size<br>3. Recruitment procedures (generalisability)<br>4. Randomization and/or stratification<br>5. Completion rates<br>6. Non responses<br>7. Question framing<br>8. Length of questions<br>9. Understandability of tasks<br>10. Piloting and pretesting	1. Respondent selection<br>2. and recruitment<br>3. Sample size<br>4. Respondent selection and recruitment (Sample selection bias)<br>5. Randomization<br>6. Missing (incomplete) data<br>7. Response rates<br>8. Question framing<br>9. Questionnaire response time<br>10. Piloting and pre testing of the study
Arnold et al. 2009 [62]	No	Not applicable: No quality appraisal done in the review	Not applicable: No quality appraisal done in the review
Wells et al 2009 *	**No**	**Case control studies (NOS for Case studies)**<br>1. Case definition adequacy<br>2. Representativeness of cases<br>3. Selection of controls<br>4. Definition of control<br>5. Comparability of cases and controls<br>6. Ascertainment of exposure<br>7. Same method of ascertainment of cases and controls<br>8. Non response rates<br>**Cohort studies (NOS for Cohort studies)**<br>9. Representative of exposed cohorts<br>10. Selection of non-exposed cohorts<br>11. Demonstration that outcome of interest was not present at the start<br>12. Assessment of outcome<br>13. Length of follow up period Adequacy of follow up of cohorts	1. Case definition<br>2. Representativeness of cases<br>3. Selection of controls<br>4. Control and contemporary groups<br>5. Confounding/Baseline equivalency of groups<br>6. Ascertainment of exposure<br>7. Ascertainment of cases and controls<br>8. Response rates<br>9. Representative of exposed cohorts<br>10. Selection of non-exposed cohorts<br>11. Absence of outcome of interest at index date<br>12. Assessment of outcome<br>13. Follow up period<br>14. Adequacy of follow up of cohorts
Mitton et al.2007 [63]	No	1. Literature Review:<br>2. Research Questions **and** Design:<br>3. Population **and** Sampling	1. Literature Review:<br>2. Study aims, objectives and rationale (Research question)

Author and year	NICE/ISPOR tools	Original list of items per tool or GPR	Assigned name per each item of a tool or GPR
		¬ Setting<br>¬ Target population<br>¬ Approach to sampling<br>4. Data Collection **and** Capture<br>¬ Concepts/measures/variables<br>¬ Data collection<br>¬ Response or participation rate<br>¬ Completeness of information capture<br>5. Analysis **and** Results Reporting<br>6. Conclusions	3. Study design features /Experimental design<br>4. Appropriateness of study country,(setting or facilities)<br>5. Appropriateness of study population<br>6. Respondent selection and recruitment (Sample selection bias)<br>7. Data collection methods/Measurement instrument<br>8. Confounding/Baseline equivalency of groups<br>9. Response rates<br>10. Missing (incomplete) data<br>11. Statistical Analysis/ Data Analysis<br>12. Reporting of results<br>13. Discussions, limitations and conclusions
Vistad et al.2006 [64]	No	1. reliability,<br>2. validity,<br>3. acceptability,<br>4. feasibility, and<br>5. appropriateness of instrument<br>6. Homogeneity of sample,<br>7. Sample size,<br>8. Use of comparison groups,<br>9. Treatment modality-specific analyses,<br>10. Time since diagnosis,<br>11. Statistics, and<br>12. Quality of the measures used.	1. Instrument reliability<br>2. Instrument validity (Face, Content and Construct)<br>3. Instrument acceptability (level of detail, comprehensibility vs burden vs cognitive overload)<br>4. Instrument feasibility<br>5. Appropriateness of instrument<br>6. Homogeneity of sample<br>7. Sample size<br>8. Control and contemporary groups<br>9. Statistical Analysis/ Data Analysis<br>10. Time of assessment<br>11. Statistical Analysis/ Data Analysis<br>12. Study design features /Experimental design
Coper et al. 2005 [65]	No	No components but rather ranking of sources of evidence as follows:<br>For utility data:<br>1. Direct utility assessment for the specific study from a sample either:<br>¬ of the general population<br>¬ with knowledge of the disease(s) of interest<br>¬ of patients with the disease(s) of interest<br>**OR**<br>Indirect utility assessment from specific study from patient sample with disease(s) of interest, using a tool validated for the patient population	1. Source of utility values<br>OR<br>Indirectness

Author and year	NICE/ISPOR tools	Original list of items per tool or GPR	Assigned name per each item of a tool or GPR
		2. Indirect utility assessment from a patient sample with disease(s) of interest, using a tool not validated for the patient population<br>3. Direct utility assessment from a previous study from a sample either:<br>¬ of the general population<br>¬ with knowledge of the disease(s) of interest<br>¬ of patients with the disease(s) of interest<br>**OR**<br>Indirect utility assessment from previous study from patient sample with disease(s) of interest, using a tool validated for the patient population<br>4. Unsourced utility data from previous study – method of elicitation unknown<br>5. Patient preference values obtained from a visual analogue scale<br>6. Delphi panels, expert opinion"	
Kmet et al. 2004 [66]	**No**	**No domains or components listed, only signaling questions**<br>1. Question / objective sufficiently described?<br>2. Study design evident and **appropriate?**<br>3. Method of subject/comparison group selection or source of information/input variables described and appropriate?<br>**4.** Subject (and comparison group, if applicable) characteristic sufficiently **described?**<br>5. If interventional and random allocation was possible was it described?<br>6. If interventional and blinding of investigators was possible, was it reported?<br>7. If interventional and blinding of subjects was possible, was it reported?<br>8. Outcome and (if applicable) exposure measure(s) well defined and robust to measurement / **misclassification bias**? Means of assessment reported?<br>9. Sample size appropriate?	1. Study aims, objectives and rationale (Research question)<br>2. Study design features /Experimental design<br>3. Respondent selection and recruitment (Sample selection bias)<br>4. Control and contemporary groups<br>5. Randomization<br>6. Blinding<br>7. Blinding<br>8. Ascertainment of exposure<br>9. Assessment of outcomes<br>10. Sample size<br>11. Statistical Analysis/ Data Analysis<br>12. Precision of estimates<br>13. Confounding/Baseline equivalency of groups<br>14. Reporting of results<br>15. Discussions, limitations and conclusions

Author and year	NICE/ISPOR tools	Original list of items per tool or GPR	Assigned name per each item of a tool or GPR
		10. Analytic methods described/justified and appropriate?<br>11. Some estimate of variance is reported for the main results?<br>12. Controlled for confounding?<br>13. Results reported in sufficient detail?<br>14. Conclusions supported by the results?	
Thomas et al. 2004 [67]<br><br>Effective Public Health Practice Project.	No	1. Study design<br>2. Selection bias<br>3. Confounding<br>4. Blinding of assessors<br>5. Blinding of participants<br>6. Validity of data collection tools<br>7. Reliability of data collection tools<br>8. Exposure to intervention<br>9. Integrity of intervention<br>10. Contamination/co-intervention<br>11. Participation rates<br>12. Withdrawals/dropouts<br>13. Data analysis	1. Study design features /Experimental design<br>2. Respondent selection and recruitment (Sample selection bias)<br>3. Confounding/Baseline equivalency of groups<br>4. Blinding<br>5. Blinding<br>6. Instrument validity (Face, Content and Construct)<br>7. Instrument reliability<br>8. Ascertainment of exposure<br>9. Integrity of intervention<br>10. Adherence to a predefined study protocol<br>11. Response rates<br>12. Loss of follow up (Attrition or withdrawals)<br>13. Statistical Analysis/ Data Analysis
Slim et al. 2003 [68]	No	1. A clearly stated study aim<br>2. Inclusion of consecutive patients<br>3. Prospective collection of data<br>4. Endpoint appropriate to the study aim<br>5. Unbiased evaluation of endpoints<br>6. Follow-up period appropriate to the major endpoint<br>7. Loss to follow up not exceeding 5%<br>8. Prospective calculation of the study size<br>**And in the case of comparative studies**<br>9. An adequate control group<br>10. Contemporary groups<br>11. Baseline equivalence of groups (confounding)<br>12. Adequate statistical analyses adapted to the study design"	1. Study aims, objectives and rationale (Research question)<br>2. Inclusion and exclusion criteria<br>3. Adherence to a predefined study protocol<br>4. Appropriateness of endpoints<br>5. Assessment of outcome<br>6. Follow up period<br>7. Loss of follow up (Attrition or withdrawals)<br>8. Sample size<br>9. Control and contemporary groups<br>10. Control and contemporary groups<br>11. Confounding/Baseline equivalency of groups<br>12. Statistical Analysis/ Data Analysis
Ryan et al 2001 [69]		1. Validity of the technique used<br>¬ Content validity<br>¬ Criterion validity	1. Instrument validity<br>2. Reproducibility<br>3. Internal consistency of results

**Author and year**	**NICE/ISPOR tools**	**Original list of items per tool or GPR**	**Assigned name per each item of a tool or GPR**
		¬ Construct validity<br>2. Reproducibility<br>3. Internal consistency<br>4. Acceptability to respondents<br>¬ Question framing<br>¬ Time to complete<br>¬ Response rate<br>¬ Completion rates<br>5. Data analysis<br>6. Generalisability<br>7. Clarity of research question<br>8. Appropriateness of the design to the question:<br>13. Sampling	4. Question framing<br>5. Questionnaire response time<br>6. Response rates<br>7. Missing (incomplete) data<br>8. Statistical Analysis/ Data Analysis<br>9. Respondent selection and recruitment (Sample selection bias<br>10. Study aims, objectives and rationale (Research question)<br>11. Study design features /Experimental design<br>13. Respondent selection and recruitment (Sample selection bias)
Lenert et al 2000 [70]	NO	**A. <u>Construct representation or underrepresentation</u>**<br>1. Health states descriptions<br>¬ Relevance of options, attributes and attribute levels.<br>¬ Sources used in the construction of options, attributes and attribute levels.<br>¬ Level of detail and accuracy of options attributes and attribute level<br>¬ Question framing<br>2. Scaling methods (SG, TTO, RSs etc.)<br>3. Cognitive skills and understandability of tasks<br>4. Protocol adherence<br>5. Protocol accessibility<br>**B. <u>Construct-irrelevant variance</u>**<br>6. Piloting and pre testing of the study<br>7. Assessment procedures<br>¬ Indifference search procedure<br>¬ Framing of the gamble<br>¬ Sequencing effect for RSs.<br>¬ Interviewer effects.<br>8. Question framing<br>9. Skills, emotions and cognitive ability of participants<br>C. Internal consistency	1. Appropriate health state description<br>2. Attributes and levels<br>3. Instrument acceptability (level of detail, comprehensibility vs burden vs cognitive overload<br>4. Technique used to value the health states<br>5. Instrument acceptability (level of detail, comprehensibility vs burden vs cognitive overload<br>6. Adherence to a predefined study protocol<br>7. Availability and accessibility of a study protocol<br>8. Piloting and pre testing of the study<br>9. Indifference search procedure<br>10. Question framing<br>11. Question and methods sequencing<br>12. Question framing<br>13. Instrument acceptability (level of detail, comprehensibility vs burden vs cognitive overload<br>14. Internal consistency
Downs and Black 1998 [71]	**No**	**Reporting Items**<br>1. The hypothesis / aim / objective<br>2. Characteristics of the patients included<br>3. Interventions of interest<br>4. Principal confounders in each group	1. Study aims, objectives and rationale (Research question)<br>2. Inclusion and exclusion criteria<br>3. Control and contemporary groups<br>4. Confounding/Baseline equivalency of groups

Author and year	NICE/ISPOR tools	Original list of items per tool or GPR	Assigned name per each item of a tool or GPR
		5. Findings of the study<br>6. Estimates of the random variability<br>7. Important adverse events<br>8. Lost to follow-up<br>9. Actual probability values<br>**External validity (3 items) – the ability to generalize findings of the study**;<br>10. Representativeness of study subjects<br>11. Representativeness of staff, settings and facilities<br>**Study bias (7 items) – to assess bias in the intervention and outcome measure(s);**<br>12. Blinding of subjects<br>13. Blinding of outcome accessors<br>14. Data dredging<br>15. Adjusting for different length of follow up<br>16. Statistical tests used for main outcomes<br>17. Compliance with the intervention/s<br>18. Main outcome measured used accurate (valid and reliable)?<br>**Confounding and selection bias (6 items) – to determine bias from sampling or group assignment;**<br>19. Baseline characteristics<br>20. Randomization<br>21. Adequate adjusting for confounding<br>22. Account for loss of follow up<br>23. Recruited over the same period of time?	5. Reporting of results<br>6. Precision of estimates<br>7. Important adverse events<br>8. Loss of follow up (Attrition or withdrawals)<br>9. P value reporting<br>10. Appropriateness of study population<br>11. Appropriateness of study country,(setting or facilities)<br>12. Blinding<br>13. Blinding<br>14. Reporting bias<br>15. Statistical Analysis/ Data Analysis<br>16. Statistical Analysis/ Data Analysis<br>17. Adherence to a predefined study protocol<br>18. Confounding/Baseline equivalency of groups<br>19. Confounding/Baseline equivalency of groups<br>20. Randomization<br>21. Respondent selection and recruitment (Sample selection bias)<br>22. Statistical Analysis/ Data Analysis<br>23. Statistical Analysis/ Data Analysis<br>24. Sample size
Jadad et al. 1996 [72]	**No**	**3 important domains for control of bias in RCT:**<br>1. Randomization<br>2. Blinding (Double blinding)<br>3. Management of withdrawals and or dropouts"	1. Randomization<br>2. Blinding<br>3. Loss of follow up (Attrition or withdrawals)

**Author and year**	**NICE/ISPOR tools**	**Original list of items per tool or GPR**	**Assigned name per each item of a tool or GPR**
Torrance 1986 [73]	**No**	**Measured utilities should:**<br>1. Match the study objective.<br>2. Subjects used should also match the study objective<br>¬ Patients<br>¬ Society<br>¬ Experts<br>3. The measurement instrument used should be credible<br>4. Health state description should be functional not clinical (MAU) -PF, EF and SF levels at least<br>5. Age of onset and duration in the health state should be described or stated<br>6. Control for confounding from other states other than the one being measured.<br>7. Description of anchor states (Death and Health (perfect health)<br>8. Level of detail (comprehensibility vs burden vs cognitive overload)<br>9. Perspective of evaluation (measuring own health or someone's health).	1. Study aims, objectives and rationale (Research question)<br>2. Population used to value the health states within the measure<br>3. Data collection methods/Measurement instrument<br>4. Appropriate health state description<br>5. Age of onset and duration in the health state<br>6. Confounding/Baseline equivalency of groups<br>7. Description and use of anchor states<br>8. Instrument acceptability (level of detail, comprehensibility vs burden vs cognitive overload)<br>9. Perspective of evaluation (measuring own health or someone's health).
Soares M and Dumville JC 2008	**Critical appraisal of cost-effectiveness and cost-utility studies in health care, not included in the analysis**		
Braizer et al. 2007	**A textbook, not included in the analysis**		
Drummond et al 2005	**A textbook, not included in the analysis**		

*No peer reviewed publication could be found for this tool. Information about the tool is available on the developer's website http://www.ohri.ca/programs/clinical_epidemiology/oxford.htm

Table A.7: Comprehensive list of items found in SLRs, QA tools, checklists and GPRs

1. Absence of outcome of interest at index date
2. Adequacy of follow up of cohorts
3. Adherence to a predefined study protocol
4. Administration procedure
5. Age of onset and duration in the health state
6. Allocation sequence concealment
7. Appropriate health state description
8. Appropriate use of generic preference-based method
9. Appropriate use of valuation method
10. Appropriateness of endpoints
11. Appropriateness of instrument
12. Appropriateness of study country,(setting or facilities)
13. Appropriateness of study population
14. Ascertainment of cases and controls
15. Ascertainment of exposure
16. Assessment of outcome
17. Availability and accessibility of a study protocol
18. Bibliographic details, including year of publication
19. Blinding
20. Case definition
21. Confounding/Baseline equivalency of groups
22. Control and contemporary groups
23. Credible extrapolation of health state valuations?
24. Data collection methods/Measurement instrument
25. Data sets
26. Data sources/Source of HSUVs
27. Description and use of anchor states

47. Instrument version
48. Integrity of intervention
49. Internal consistency of results
50. Intervention measurement
51. Interventions ,control and contemporary groups
52. Literature Review:
53. Loss of follow up (Attrition or withdrawals)
54. Measure used to describe the HSUVs
55. Misclassification bias
56. Missing (incomplete) data
57. Model Selection and Performance
58. Modeling techniques
59. Non-normality of the distribution of utility values
60. Other sources of bias
61. P value reporting
62. Perspective of evaluation (measuring own health or someone's health).
63. Population the HSUVs collected from
64. Population used to value the health states within the measure
65. Precision of estimates
66. Proxy rates by mRS level?
67. Randomization
68. Recruitment period
69. Reporting bias
70. Reporting of results
71. Representative of exposed cohorts
72. Representativeness of cases
73. Respondent selection and recruitment (Sample selection bias)
74. Response rates

28. Description of basic data
29. Discussions, limitations and conclusions
30. Ethical approval
31. Follow up period
32. Generalizability of findings
33. Homogeneity of sample
34. Important adverse events
35. Inclusion and exclusion criteria
36. Indifference search procedure
37. Indirectness
38. Instrument acceptability (level of detail, comprehensibility vs burden vs cognitive overload)
39. Instrument feasibility
40. Instrument reliability
41. Instrument responsiveness
42. Instrument validity (Face, Content and Construct)
43. Attributes and levels
44. Understanding of participants
45. Outcomes assessed
46. Piloting and pre-testing of the study
75. Sample size
76. Scoring algorithm
77. Selection of controls
78. Selection of non-exposed cohorts
79. Sensitivity analysis
80. Sensitivity of instrument
81. Source of tariff (value set)
82. Source of utility values
83. Sources of funding
84. Statistical Analysis/ Data Analysis
85. Study aims, objectives and rationale (Research question)
86. Study design features /Experimental design
87. Technique used to value the health states
88. Time of assessment
89. Publication bias
90. Reproducibility
91. Question framing
92. Question and methods sequencing
93. Questionnaire response time

b. Items recommended by NICE, ISPOR and related publications (ISPOR items)

Reporting and RoB

1. Sample size.
2. Respondent selection and recruitment.
3. Inclusion and exclusion criteria.
4. Response rates to instrument used.
5. Loss to follow-up.
6. Missing (incomplete) data.
7. Any other problems with the study: Example: Relevance of location (e.g. if patients recruited in non-UK country).

Relevancy questions

8. Measure used to describe the HSUVs.
9. Population the HSUVs collected from.
10. Population used to value the health states within the measure.
11. Technique used to value the health state.

Appropriateness of measure (Measurement instrument)

12. Appropriate use of valuation method.
13. Appropriate use of generic preference-based method.
14. Appropriate health state description.

c. Additional items considered "relevant" based on literature, theoretical considerations and conceptual understanding of HSUV elicitation

1. Adherence to a predefined study protocol.
2. Administration procedure.
3. Blinding.
4. Confounding/Baseline equivalency of groups.
5. Indifference search procedure.
6. Randomization.
7. Reporting bias.
8. Reporting of results.
9. Source of funding.
10. Source of utility values.
11. Source of tariff.
12. Statistical analysis/ Data Analysis.
13. Study design features /Experimental design.
14. Time of assessment or follow up.

Supplementary material 6: Characteristics of included SLRs, tools and good recommendation practices

Table A.8: Detailed characteristics of included SLRs

Author	Study objectives	Disease type	Quality appraisal	Type of tool used	Critical assessment tool format	Scope or type of data synthesis	Quality appraisal focus	How where QA appraisal results incorporated into data synthesis?
**Afshari et al. 2021 [17]**	To explore the psychometric properties of the EQ-5D-5L instrument in patients with asthma and identify the EQ-5D-5L scores in these patients.	Asthma	Yes	Standard tool	Scale (Score Based)	Both Qualitative and Quantitative	Reporting quality only	Exclude studies at high or unclear risk of bias (or moderate or low quality) from the synthesis
**Carrello et al. 2021 [18]**	To conduct a systematic review and meta-analysis of health state utility decrements associated with overweight and obesity in adults 18 years and over, for use in modelled economic evaluations in Australia	Overweight and Obesity	Yes	Adapted tool	Domain Based	Quantitative Synthesis	RoB only	Narrative discussion (with minimal evidence for incorporation deemed acceptable)
**Di Tanna et al. 2021* [1]**	To identify and summarize heart failure utility values for use in economic evaluations.	Heart Failure	Yes	Adapted tool	Checklist	Qualitative Synthesis	Reporting, RoB(Methodological) and Relevancy	Narrative discussion (with minimal evidence for incorporation deemed acceptable)
**Han et al. 2021 [20]**	To identify HSUVs used in cost-utility analyses (CUAs) for CHC in Europe and to evaluate the impact of HSUV selection on cost-effectiveness results in terms of the incremental cost per quality-adjusted life-year (QALY) gained (ICER).	Chronic Hepatitis C	Yes	Custom made (Ad-hoc tool)	Checklist	Quantitative Synthesis	Reporting, RoB(Methodological) and Relevancy	Sensitivity analysis
**Haridoss et al. 2021[21]**	To identify EQ-5D utility scores of RA from Asia to provide a pooled estimate of EQ-5D utility and EQ-5D VAS scores.	Rheumatoid arthritis	Yes	Standard tool	Checklist, Scale and Domain based	Both Qualitative and Quantitative	Reporting, RoB(Methodological) and Relevancy	Narrative discussion (with minimal evidence for incorporation deemed acceptable)
**Jiang et al. 2021 [22]**	To conduct a systematic review and meta-analysis to compare differences in health	Not specific (Children)	Yes	Both Standard Tool and	Scale (Score Based)	Both Qualitative	0	Exclude studies at high or unclear risk of bias (or

Author	Study objectives	Disease type	Quality appraisal	Type of tool used	Critical assessment tool format	Scope or type of data synthesis	Quality appraisal focus	How where QA appraisal results incorporated into data synthesis?
	utilities (HUs) assessed by self and proxy respondents in children, as well as to evaluate the effects of health conditions, valuation methods, and proxy types on the differences.	34 Health conditions		Custom made. (two tools were used)		and Quantitative		moderate or low quality) from the synthesis
**Park et al. 2021 [23]**	To evaluate the HRQOL based on EQ-5D utility score according to various health statuses of TB patients.	Tuberculosis	Yes	Standard tool	Both Scale and Domain based	Both Qualitative and Quantitative	RoB only	Narrative discussion (with minimal evidence for incorporation deemed acceptable)
**Aceituno et al. 2020* [2]**	To synthesize the evidence about HSUVs in schizophrenia.	Schizophrenia	Yes	Custom made (Ad-hoc tool)	Checklist	Both Qualitative and Quantitative	Reporting, RoB(Methodological) and Relevancy	Narrative discussion (with minimal evidence for incorporation deemed acceptable)
**Blom et al. 2020* [3]**	To provide a current systematic review of HSUVs for all types of lung cancer, including an overview of study characteristics and a critical appraisal, and a pooled set of community- and choice-based HSUVs for use in economic evaluations.	Lung Cancer	Yes	Custom made (Ad-hoc tool)	Both Checklist and Domain based	Both Qualitative and Quantitative	Reporting and RoB(Methodological) and Relevancy	Exclude studies at high or unclear risk of bias (or moderate or low quality) from the synthesis
**Cooper et al. 2020* [4]**	To identify HSU weights for different stages of chronic kidney disease (CKD), renal replacement therapy (RRT) and complications.	Chronic kidney disease	Yes	Custom made (Ad-hoc tool)	Both Checklist and Domain based	Qualitative Synthesis	RoB and Relevancy	Exclude studies at high or unclear risk of bias (or moderate or low quality) from the synthesis
**Golicki et al. 2020* [5]**	To provide a catalog of EQ-5D-based HSUVs for various hematologic malignancies, based on a systematic review of available evidence.	Hematologic malignancies (C81-C86, C88, C90-C94, C96)	Yes	Both Standard Tool and Custom made (two tools were used)	Both Scale and Checklist	Qualitative Synthesis	Reporting, RoB(Methodological) and Relevancy	Narrative discussion (with minimal evidence for incorporation deemed acceptable)
**Landeiro et al. 2020 [24]**	To provide an overview of which quality of life instruments are being used to	Predementia Alzheimer's disease [AD]	Yes	Standard tool	Domain Based	Both Qualitative	RoB only	Narrative discussion (with minimal evidence for

Author	Study objectives	Disease type	Quality appraisal	Type of tool used	Critical assessment tool format	Scope or type of data synthesis	Quality appraisal focus	How where QA appraisal results incorporated into data synthesis?
	assess HR-QoL in people with predementia AD, MCI or dementia; and, to summarise their reported HR-QoL levels at each stage of the disease and by type of respondent.	(preclinical or prodromal AD), Mild cognitive impairment (MCI) and Dementia				and Quantitative		incorporation deemed acceptable)
**Petrou et al. 2020* [6]**	To perform a systematic review and meta-analysis of health utility values associated with preterm birth generated using preference-based approaches to health-related quality of life measurement.	Preterm Birth	Yes	Adapted tool	Scale (Score Based)	Both Qualitative and Quantitative	Reporting, RoB(Methodological) and Relevancy	No attempt to incorporate quality assessment findings into systematic review findings
**Rebchuk et al. 2020 [25]**	To characterize the between-study variability in utility weighting of the mRS in a population of patients who experienced stroke and its implications when applied to the results of a clinical trial.	Stroke	Yes	Custom made (Ad-hoc tool)	Checklist	Quantitative Synthesis	Reporting quality	No attempt to incorporate quality assessment findings into systematic review findings
**Saeed et al. 2020* [7]**	To summarize the available data on utilities in CHC patients and facilitate analyses of CHC treatment and elimination strategies.	Chronic hepatitis C (CHC)	Yes	Adapted tool	Checklist	Both Qualitative and Quantitative	Reporting, RoB(Methodological) and Relevancy	No attempt to incorporate quality assessment findings into systematic review findings
**Szabo et al. 2020* [8]**	To synthesize published evidence for health state utility from the DMD patient and caregiver perspectives.	Duchenne muscular dystrophy	Yes	Adapted tool	Scale (Score Based)	Qualitative Synthesis	RoB and Relevancy	No attempt to incorporate quality assessment findings into systematic review findings
**Yang et al. 2020 [26]**	To determine pooled EQ-5D utility scores for psoriasis as a general condition, plaque psoriasis, and psoriatic arthritis.	Psoriasis	Yes	Adapted tool	Domain Based	Both Qualitative and Quantitative	RoB only	Narrative discussion (with minimal evidence for incorporation deemed acceptable)
**Yuan et al. 2020[27]**	To determine pooled estimates of utility-based quality of life in patients with CSU.	Chronic Spontaneous Urticaria (CSU)	Yes	Standard tool	Scale (Score Based)	Both Qualitative and Quantitative	Reporting quality only	No attempt to incorporate quality assessment findings into systematic review findings

Author	Study objectives	Disease type	Quality appraisal	Type of tool used	Critical assessment tool format	Scope or type of data synthesis	Quality appraisal focus	How where QA appraisal results incorporated into data synthesis?
**Buchanan-Hughes et al. 2019* [9]**	To identify and summarize the current evidence base for health state utility values (HSUVs) in patients with chronic hepatitis C infection, generated using the EuroQol 5-dimensions (EQ-5D) questionnaire.	Hepatitis C	Yes	Adapted tool	Checklist	Both Qualitative and Quantitative	RoB and Reporting	Narrative discussion (with minimal evidence for incorporation deemed acceptable)
**Foster et al. 2019 [28]**	To describe instruments used to estimate utilities in epilepsy populations, and how results (mean HSUVs) differ according to methods use.	Epilepsy	Yes	Standard tool	Scale (Score Based)	Qualitative Synthesis	Reporting, RoB (Methodological) and Relevancy	No attempt to incorporate quality assessment findings into systematic review findings
**Hatswell et al. 2019 [29]**	To provide an internally consistent set of utility estimates across the entire disease pathway that has been drawn from the same source data and patients, and then synthesise all available data (including registry data) that can be used in health economic modelling by conducting a systematic review augmented with analysis of primary registry data.	Multiple Myeloma	Yes	Unknow n	Unknown	Both Qualitative and Quantitative	RoB only	Unknown
**Khadka et al. 2019 [30]**	To investigate the degree of convergence in childhood utilities generated directly or indirectly within dyad child and proxy assessments.	Not specific Childhood conditions	Yes	Custom made (Ad-hoc tool)	Scale (Score Based)	Qualitative Synthesis	Reporting quality only	No attempt to incorporate quality assessment findings into systematic review findings
**Li et al. 2019* [10]**	To evaluate disutility related to cancer screening applying a utility theory framework.	Multiple Cancer (breast, cervical, lung, colorectal, and prostate cancer screening)	Yes	Adapted tool	Both Scale and Domain based	Qualitative Synthesis	Reporting, RoB(Methodological) and Relevancy	No attempt to incorporate quality assessment findings into systematic review findings

Author	Study objectives	Disease type	Quality appraisal	Type of tool used	Critical assessment tool format	Scope or type of data synthesis	Quality appraisal focus	How where QA appraisal results incorporated into data synthesis?
**Magnus et al. 2019* [11]**	To provide up-to-date utility values along the prostate cancer care continuum (i.e., from prescreening through to palliative care) for use where future trial-based or modelled economic evaluations cannot collect primary data from men and/or partners.	Prostate Cancer	Yes	Custom made (Ad-hoc tool)	Scale (Score Based)	Both Qualitative and Quantitative	Reporting, RoB (Methodological) and Relevancy	Narrative discussion (with minimal evidence for incorporation deemed acceptable)
**Van Wilder et al. 2019 [31]**	To develop a catalogue with EQ-5D scores in chronic non-communicable diseases, and to compare these scores with reference values from the general population.	Chronic non-communicable diseases Cancers, endocrine disorders, eye and adnexa, ear and mastoid, respiratory etc.)	Yes	Adapted tool	Scale (Score Based)	Qualitative Synthesis	RoB and Reporting	Narrative discussion (with minimal evidence for incorporation deemed acceptable)
**Kwon et al. 2018 [32]**	To conduct the first comprehensive systematic review and meta-analysis of primary utility data for childhood conditions and descriptors, and to determine the effects of methodological factors on childhood utilities.	Not specific Childhood conditions (reported based on ICD-10 classification)	Yes	Custom made (Ad-hoc tool)	Scale (Score Based)	Both Qualitative and Quantitative	Reporting quality only	No attempt to incorporate quality assessment findings into systematic review findings
**Paracha et al. 2018* [12]**	To identify utility scores for patients with metastatic non-small cell lung cancer (mNSCLC), as well as disutilities or utility decrements relevant to the experience of patients with mNSCLC, by treatment line and health state.	Lung cancer	Yes	Custom made (Ad-hoc tool)	Checklist	Qualitative Synthesis	RoB and Reporting	No attempt to incorporate quality assessment findings into systematic review findings
**Tran et al. 2018 [33]**	To determine pooled estimates of utility based HRQOL (utilities) for people with	Melanoma	Yes	Adapted tool	Domain Based	Quantitative Synthesis	RoB only	Narrative discussion (with minimal evidence for

Author	Study objectives	Disease type	Quality appraisal	Type of tool used	Critical assessment tool format	Scope or type of data synthesis	Quality appraisal focus	How where QA appraisal results incorporated into data synthesis?
	American Joint Cancer Committee stage I/II, III or IV melanoma for use in economic evaluations.							incorporation deemed acceptable)
**Meregaglia et al. 2017* [13]**	To identify HSUVs in head and neck cancer and provides guidance for selecting them from a growing body of health-related quality of life studies.	Head and neck cancer	Yes	Adapted tool	Scale (Score Based)	Qualitative Synthesis	Reporting quality only	No attempt to incorporate quality assessment findings into systematic review findings
**Ó Céilleachair et al. 2017 [34]**	To review the effects of a screen test on EQ-5D HRQoL (utilities) for cervical prevention and the corresponding effect on CEA of screening.	Cervical diseases (including cancer and its pre cursor lesions)	Yes	Custom made (Ad-hoc tool)	Scale (Score Based)	Qualitative Synthesis	RoB and Reporting	Narrative discussion (with minimal evidence for incorporation deemed acceptable)
**Ward Fuller et al. 2017 [35]**	To characterize existing HSPW estimates and model the EuroQol five-dimensional questionnaire (EQ-5D) from the GOS, to inform parameterization of future economic models.	Brain Injury	Yes	Custom made (Ad-hoc tool)	Domain Based	Qualitative Synthesis	RoB only	No attempt to incorporate quality assessment findings into systematic review findings
**Zrubka et al. 2017 [36]**	To assess the state of EQ-5D research in musculoskeletal disorders in 8 Central and Eastern European countries and to provide a meta-analysis of EQ-5D index scores.	Musculoskeletal and connective tissue disorders: including rheumatoid arthritis (RA), ankylosing spondylitis (AS), psoriatic arthritis (PsA), osteoporosis (OP), and systemic lupus erythematosus (SLE)	Yes	Custom made (Ad-hoc tool)	Domain Based	Both Qualitative and Quantitative	RoB and Reporting	Narrative discussion (with minimal evidence for incorporation deemed acceptable)

Author	Study objectives	Disease type	Quality appraisal	Type of tool used	Critical assessment tool format	Scope or type of data synthesis	Quality appraisal focus	How where QA appraisal results incorporated into data synthesis?
**Kua et al. 2016* [14]**	To identify preference-based utility values for children with day-to-day asthma symptoms (baseline utility) and children experiencing an asthma exacerbation, and to review the appropriateness of the utility values to be used in the PLEASANT economic analysis.	Asthma	Yes	Adapted tool	Domain Based	Qualitative Synthesis	Reporting, RoB(Methodological) and Relevancy	No attempt to incorporate quality assessment findings into systematic review findings
**Paracha et al. 2016* [15]**	To identify health state utility values (HSUVs) for late-stage breast cancer, derived using methods preferred by health technology assessment (HTA) agencies, by treatment line.	Breast cancer	Yes	Custom made (Ad-hoc tool)	Domain Based	Qualitative Synthesis	RoB and Reporting	Narrative discussion (with minimal evidence for incorporation deemed acceptable)
**Brennan et al. 2015 [37]**	To identify studies eliciting utility decrements from myocardial infarction and stroke in patients with Type 2 Diabetes Mellitus and review how these have been used in economic models of new diabetes treatments.	Myocardial infarction or stroke related type 2 Diabetes Mellitus	Yes	Custom made (Ad-hoc tool)	Checklist	Qualitative Synthesis	RoB and Relevancy	Narrative discussion (with minimal evidence for incorporation deemed acceptable)
**Carter et al. 2015* [16]**	To systematically identify utility values associated with advanced gastric cancer (GC), esophageal cancer (OC), or gastro-esophageal junction (GEJ) cancer.	Advanced gastric, esophageal, or gastroesophageal junction cancer	Yes	Adapted tool	Domain Based	Qualitative Synthesis	Reporting, RoB(Methodological) and Relevancy	Narrative discussion (with minimal evidence for incorporation deemed acceptable)
**Gheorghe et al. 2015 [39]**	To summarize the evidence base on Surgical Site Infection health utility values reported in patient-level studies and decision models.	Not specific (Surgical site infection)	Yes	Custom made (Ad-hoc tool)	Domain Based	Qualitative Synthesis	Relevancy only	No attempt to incorporate quality assessment findings into systematic review findings

Author	Study objectives	Disease type	Quality appraisal	Type of tool used	Critical assessment tool format	Scope or type of data synthesis	Quality appraisal focus	How where QA appraisal results incorporated into data synthesis?
**Li et al. 2015 [40]**	To systematically review the literature to determine if utilities (a quantitative way to express patient preferences for health outcomes) have been measured in hand surgery studies.	Not specific (Hand surgery)	Yes	Both Adapted and Custom made. (two tools were used)	Both Scale and Checklist	Qualitative Synthesis	Reporting quality only	No attempt to incorporate quality assessment findings into systematic review findings
**Brockbank et al. 2021 [74]**	To identify health state utility values (HSUVs) and disutilities in MDD for use in future economic evaluations of pharmacological treatments.	Major depressive disorder	No	Not Applicab le	Not Applicable		Not Applicable	Not Applicable
**Chataway et al. 2021 [75]**	To identify systematically the available evidence of the economic burden and HSUVs associated with multiple sclerosis (MS) and report the burden of Secondary progressive multiple sclerosis (SPMS) compared with relapsing–remitting multiple sclerosis (RRMS).	Multiple sclerosis	No	Not Applicab le	Not Applicable		Not Applicable	Not Applicable
**Houten et al. 2021 [76]**	To summarise the utility values available to represent the health-related quality of life (HRQoL) of patients with thyroid cancer.	Thyroid cancer	No	Not Applicab le	Not Applicable		Not Applicable	Not Applicable
**Thorrington et al. 2015 [77]**	To evaluate the use (application) of all direct and indirect methods used to estimate health utilities in both children and adolescents.	Not specific (children)	No	Not Applicab le	Not Applicable		Not Applicable	Not Applicable
**Betts et al. 2020 [78]**	To identify the most recent utility value estimates for cardiovascular disease (CVD) via systematic literature review (SLR) and explore	Cardiovascula r disease (CVD) (Stroke, Stable Angina,	No	Not Applicab le	Not Applicable		Not Applicable	Not Applicable

Author	Study objectives	Disease type	Quality appraisal	Type of tool used	Critical assessment tool format	Scope or type of data synthesis	Quality appraisal focus	How where QA appraisal results incorporated into data synthesis?
	trends in utility elicitation methods in the last 6 years.	Undefined/ Unstable Angina, Revascularization, Myocardial Infarction)						
**Etxeandia-Ikobaltzeta et al. 2020 [19]**	To inform the American Society of Hematology guidelines for management of venous thromboembolism (VTE) disease.	Venous thromboembolism (VTE) disease	Yes	Standard tool	Domain Based	Qualitative Synthesis	RoB only	Narrative discussion (with minimal evidence for incorporation deemed acceptable)
**Mohindru et al. 2020 [79]**	To provide a summary of HSU-related research in CF and highlight related research gaps.	Cystic Fibrosis	No	Not Applicable	Not Applicable		Not Applicable	Not Applicable
**Xia et al. 2020 [80]**	To generate a database of HSUs that could be used to populate future model-based cost-utility analyses of bariatric surgery procedures through:<br>1. Obtaining bariatric surgery-related pooled HSUs (from baseline [pre-surgery] to various post-surgery time points) and HSU changes over time (where feasible) that can<br>2. Undertaking a systematic overview of published studies reporting HSU-based HRQoL in patients who underwent bariatric surgery procedures be used to adjust life expectancy in future CUA.	Bariatric surgery procedures	No	Not Applicable	Not Applicable		Not Applicable	Not Applicable
**Zhao et al. 2020 [81]**	To systematically review and meta-analyse the HSUVs for people with OA.	Osteoarthritis (OA)	No	Not Applicable	Not Applicable		Not Applicable	Not Applicable

Author	Study objectives	Disease type	Quality appraisal	Type of tool used	Critical assessment tool format	Scope or type of data synthesis	Quality appraisal focus	How where QA appraisal results incorporated into data synthesis?
**Prevolnik Rupel et al. 2019 [82]**	To systematically review and analyse the available empirical studies using the EQ-5D instrument as a measure of the health-related quality of life (HRQoL) in patients with neurological diseases.	Neurological diseases(Multiple sclerosis, Parkinson's disease, Stroke, Neuropathic pain, Epilepsy, Essential tremor, Dystonia, Carpal tunnel syndrome, Duchenne muscular dystrophy)	No	Not Applicable	Not Applicable		Not Applicable	Not Applicable
**Shiri et al. 2019 [83]**	To summarize evidence on resource use, costs, health utilities, and cost-effectiveness for pneumococcal disease and associated interventions to inform future economic analyses.	Pneumococcal disease<br>1. Invasive pneumococcal disease that includes meningitis, septicemia or bacteremia, and empyema<br>2. Noninvasive pneumococcal disease that includes community-acquired pneumonia (CAP), sinusitis, and otitis media	No	Not Applicable	Not Applicable		Not Applicable	Not Applicable
**Tonmukayakul et al. 2019 [84]**	1. To identify reported utility values associated with CP in children aged ≤ 18 years.<br>2. To examine performance of	cerebral palsy(Children)	No	Not Applicable	Not Applicable		Not Applicable	Not Applicable

Author	Study objectives	Disease type	Quality appraisal	Type of tool used	Critical assessment tool format	Scope or type of data synthesis	Quality appraisal focus	How where QA appraisal results incorporated into data synthesis?
	the measures and/or elicitation approaches.<br>3. To explore utility value elicitation techniques in published studies.							
**Wittenberg et al. 2019 [85]**	To catalog spillover-related health utilities to facilitate their consideration in CEAs.	Multiple patient diseases (not specific)	No	Not Applicab le	Not Applicable		Not Applicable	Not Applicable
**Yoon et al. 2019 [86]**	To compile a repository of utility scores in breast cancer and to assess gaps in the current literature.	Breast Cancer	No	Not Applicab le	Not Applicable		Not Applicable	Not Applicable
**Zhou et al. 2019 [87]**	To review utility values used in schizophrenia Pharmacoeconomics evaluations and evaluated the impact of their selection on the incremental cost-effectiveness ratio (ICER).	Schizophrenia	No	Not Applicab le	Not Applicable		Not Applicable	Not Applicable
**Batóg et al. 2018 [88]**	To systematically review EQ-5D literature on cardiovascular diseases in eight Central and Eastern European (CEE) countries.	Cardiovascula r diseases	No	Not Applicab le	Not Applicable		Not Applicable	Not Applicable
**Blieden Betts et al. 2018 [89]**	To explore how methods of elicitation impact utility values for Cardiovascular diseases (Variability in utility values based on the method of elicitation, tariff, or type of respondent).	Cardiovascula r disease(Stoke MI, Angina and Heart Failure)	No	Not Applicab le	Not Applicable		Not Applicable	Not Applicable
**Brown et al. 2018 [90]**	To systematically review studies reporting utility values by weight status in children and adolescents aged ≤18 years.	Childhood obesity	No	Not Applicab le	Not Applicable		Not Applicable	Not Applicable
**Dossa et al. 2018 [91]**	To identify studies reporting utilities for colostomies or ileostomies using direct or	Ileostomy and Colostomy irrespective of	No	Not Applicab le	Not Applicable		Not Applicable	Not Applicable

Author	Study objectives	Disease type	Quality appraisal	Type of tool used	Critical assessment tool format	Scope or type of data synthesis	Quality appraisal focus	How where QA appraisal results incorporated into data synthesis?
	indirect, preference-based elicitation tools and summarise (pooled estimates) based on elicitation groups.	primary reason (CRC, GIT diseases etc.)						
**Forsythe et al. 2018 [92]**	To review AML-related HSUVs that could be used in economic evaluation studies.	Acute myeloid leukemia	No	Not Applicab le	Not Applicable		Not Applicable	Not Applicable
**Li Li et al. 2018**	To assess the performance of available utility-based instruments for people with dementia by comparing their psychometric properties and to explore factors that cause variations in the reported health state values generated from those instruments by conducting meta-regression analyses.	Dementia	No	Not Applicab le	Not Applicable		Not Applicable	Not Applicable
**Vo et al. 2017 [93]**	To define the status of using quality of life or health-related quality of life instruments and to find out which articles can be applied to the process of establishing utility scores in Vietnam.	Not defined General population	No	Not Applicab le	Not Applicable		Not Applicable	Not Applicable
**Blanchard et al. 2016 [94]**	To provide a qualitative assessment of preferences (both ordinal -choices/ranking and HSUV in Head and neck cancer patients.	Head and neck cancer	No	Not Applicab le	Not Applicable		Not Applicable	Not Applicable
**Herdman et al. 2016 [95]**	To investigate how utility weights have been elicited and used in this context.	17 infectious diseases Anogenital warts; nfluenza; Hepatitis B; Haemophilus influenzae type B; Measles;	No	Not Applicab le	Not Applicable		Not Applicable	Not Applicable

Author	Study objectives	Disease type	Quality appraisal	Type of tool used	Critical assessment tool format	Scope or type of data synthesis	Quality appraisal focus	How where QA appraisal results incorporated into data synthesis?
		Meningitis ; Pertussis; Pneumococcal disease; Rotavirus; Varicella						
**Jeong et al. 2016 [96]**	To review CRC-related HSUVs that could be used in economic evaluation and assesses their advantages and disadvantages with respect to valuation methods used and CRC clinical pathways.	Colorectal cancer	No	Not Applicab le	Not Applicable		Not Applicable	Not Applicable
**Malinowski et al. 2016 [97]**	To collect and summarize the current data on the utilities of patients with Crohn's disease (CD) and ulcerative colitis (UC).	Crohn's disease	No	Not Applicab le	Not Applicable		Not Applicable	Not Applicable
**Moayeri et al. 2016 [98]**	To estimate mean utility value in COPD using meta-analysis and explore degree of heterogeneity in the utility values across a variety of clinical and study characteristic.	Chronic Obstructive Pulmonary Disease (COPD)	No	Not Applicab le	Not Applicable		Not Applicable	Not Applicable
**Smith-Palmer et al. 2016 [99]**	To identify and compare published health state utility values for adults with type 1 diabetes both, with and without diabetes-related complications.	Type 1 diabetes Mellitus	No	Not Applicab le	Not Applicable		Not Applicable	Not Applicable
**Stevanović et al. 2016 [100]**	To summarize and synthesize instrument-specific preference-based values in CHD and the underlying disease-subgroups, stable angina and post-acute coronary syndrome (postACS), for developed countries, while accounting for	Coronary Heart Disease	No	Not Applicab le	Not Applicable		Not Applicable	Not Applicable

Author	Study objectives	Disease type	Quality appraisal	Type of tool used	Critical assessment tool format	Scope or type of data synthesis	Quality appraisal focus	How where QA appraisal results incorporated into data synthesis?
	study-level characteristics, and within and between-study correlation.							
**Møller et al. 2015 [101]**	To understand the disutility of patients with psoriasis vulgaris, using mean baseline EuroQoL five dimensions (EQ-5D) index scores reported in the published literature, and to compare this to patients with other chronic diseases.	Psoriasis	No	Not Applicable	Not Applicable		Not Applicable	Not Applicable
**Thorrington et al. 2015 [77]**	To evaluate the use (application) of all direct and indirect methods used to estimate health utilities in both children and adolescents.	Not specific (children)	No	Not Applicable	Not Applicable		Not Applicable	Not Applicable
**Tran et al. 2015 [102]**	To examine the performance of preference-based instruments, estimate health utility of patients with HIV/AIDS by disease stages, and investigate changes in their health utility over the course of antiretroviral treatment.	HIV/AIDS	No	Not Applicable	Not Applicable		Not Applicable	Not Applicable

*Based on NICE/ISPOR tools

Table A.9: Detailed characteristics of the tools and good practice recommendations commonly used for QA in SLR of HSUVs

Author and year	ISPOR, NICE and related publications	Title of original publication	Document Type 2	Applicable study type(s)	Type of tool	QA components incorporated
**Brazier et al. 2019 [41]**	Yes	International Society of Pharmacoeconomics and Outcomes Research: Identification, review and use of health state utility data in cost-effectiveness models: good practices for outcomes research	Technical Document (Recommendations)	HSUVs	Not applicable	RoB and Relevancy
**Zhang et al 2019[47]**	No	GRADE Guidelines: 19. Assessing the certainty of evidence in the importance of outcomes or values and preferences-Risk of bias and indirectness	Standardized Tool	Importance of outcomes or values and preference studies	Domain based (Ranking)	Risk of bias<br>Indirectness
**Zhang et al 2019[103]**	No	GRADE guidelines: 20. Assessing the certainty of evidence in the importance of outcomes or values and preferences-inconsistency, imprecision, and other domains	Standardized Tool	Importance of outcomes or values and preference studies	Domain based (Ranking	Inconsistency<br>Imprecision<br>Publication bias<br>Reporting bias
**Petrou et al. 2018 [48]**	Yes	A practical guide to conducting a systematic review and meta-analysis of health state utility values	Technical Document (Recommendations)	HSUVs	Not applicable	Reporting, RoB(Methodological) and Relevancy
**Ara et al. 2017**	Yes	The identification, review and synthesis of health state utility values from the literature	Technical Document (Recommendations) with a QA tool developed or added	HSUVs	Checklist	Reporting, RoB(Methodological) and Relevancy
**Wolowacz et al. 2016 [55]**	Yes	Estimating Health-State Utility for Economic Models in Clinical Studies: An ISPOR Good Research Practices Task Force Report	Technical Document (Recommendations)	HSUVs	Not applicable	RoB only
**Papaioannou et al. 2013 [44]**	Yes	Systematic searching and selection of health state utility values from the literature	Technical Document (Recommendations) with a QA tool developed or added	Multiple studies for HSUVs	Checklist	Reporting, RoB(Methodological) and Relevancy
**Papaioannou et al. 2010 [43]**	Yes	NICE DSU Technical Support Document 9: The Identification, Review and	Technical Document (Recommendations)	HSUVs	Checklist	Reporting, RoB(Methodological) and Relevancy

Author and year	ISPOR, NICE and related publications	Title of original publication	Document Type 2	Applicable study type(s)	Type of tool	QA components incorporated
		Synthesis of Health State Utility Values from the Literature	with a QA tool developed or added			
**Sterne et al. 2019 [45]**	No	RoB 2: A revised tool for assessing risk of bias in randomized trials.	Standardized Tool	Randomized Trials	Domain based (Ranking)	RoB only
**Xie et al. 2019 [104]**	No	Toward a Centralized, Systematic Approach to the Identification, Appraisal, and Use of Health State Utility Values for Reimbursement Decision Making: Introducing the Health Utility Book (HUB).	Technical Document (Recommendations)	HSUVs	Not applicable	Reporting, RoB(Methodological) and Relevancy
**Wailoo et al. 2017 [49]**	No	Mapping to estimate health-state utility from non-preference-based outcome measures: an ISPOR good practices for outcomes research task force.	Technical Document (Recommendations) with a QA tool developed or added	Mapping studies of any type	Not applicable	Reporting quality only
**Downes et al. 2016 [50]**	No	Development of a critical appraisal tool to assess the quality of cross-sectional studies (AXIS.)	Standardized Tool	Cross-sectional studies (CSSs).	Checklist	Reporting, RoB(Methodological) and Relevancy
**Gupta et al. 2016 [51]**	No	Silent Brain Infarction and Risk of Future Stroke: A Systematic Review and Meta-Analysis. Stroke.	Systematic literature review(SLR)	Observational longitudinal	Checklist	Reporting and RoB
**O'Connor et al. 2016 [105]**	No	Adverse psychological outcomes following colposcopy and related procedures: a systematic review.	Systematic literature review(SLR)	Not applicable	Not applicable	Not applicable
**Sterne et al. 2016 [53]**	No	ROBINS-I: a tool for assessing risk of bias in non-randomized studies of interventions.	Standardized Tool	Non-randomized studies of interventions (NRSI)<br>1. Observational studies such as cohort studies and case-control studies<br>2. quasi-randomized studies	Domain based (Ranking)	RoB only
**Gupta et al. 2015 [54]**	No	Plaque echolucency and stroke risk in asymptomatic carotid stenosis: a systematic review and meta-analysis. Stroke.	Systematic literature review(SLR)	Prospective observational studies	Domain based (Ranking)	RoB only

Author and year	ISPOR, NICE and related publications	Title of original publication	Document Type 2	Applicable study type(s)	Type of tool	QA components incorporated
**Calvert et al. 2013 [56]**	No	Reporting of patient-reported outcomes in randomized trials: the CONSORT PRO extension.	Standardized Tool	Randomized trials (reporting PRO outcomes -health-related quality of life (HRQL), symptoms, utilities, and satisfaction ratings and are defined as assessments that are patient reported rather than observer reported)	Checklist	Reporting quality only
**Herzog et al. 2013 [57]**	No	Are healthcare workers' intentions to vaccinate related to their knowledge, beliefs and attitudes? A systematic review.	Systematic literature review(SLR)	Case control and cohort studies	Checklist	RoB only
**Braizer et al. 2011 [58]**	No	NICE DSU Technical Support Document 8: An Introduction to the Measurement and Valuation of Health for NICE Submissions.	Technical Document (Recommendations)	HSUVs	Not applicable	Not applicable
**Higgins et al. 2011 [106]**	No	The Cochrane Collaboration's tool for assessing risk of bias in randomized trials.	Standardized Tool	Randomized trials	Domain based (Ranking)	RoB only
**Park et al. 2011 [60]**	No	Risk of Bias Assessment tool for Non-randomized Studies (RoBANS): Development and validation of a new instrument.	Standardized Tool	Nonrandomized studies	Domain based (Ranking)	RoB only
**Accent and Rand Europe 2010 [61]**	No	Review of stated preference and willingness to pay methods.	Technical Document (Recommendations)	WTP studies	Not applicable	Reporting, RoB(Methodological) and Relevancy
**Arnold et al. 2009 [62]**	No	Comparison of direct and indirect methods of estimating health state utilities for resource allocation: review and empirical analysis.	Systematic literature review(SLR)	No quality appraisal was done in the review, yet it is cited as an inspiration for QA of SLRs reviewed		
**Wells et al. 2009 ***	No	Critical evaluation of the Newcastle-Ottawa scale for the assessment of the quality of nonrandomized studies in meta-analyses.	Standardized Tool	Case control and cohort studies	Domain based (Ranking)	RoB only

Author and year	ISPOR, NICE and related publications	Title of original publication	Document Type 2	Applicable study type(s)	Type of tool	QA components incorporated
**Coper et al. 2007 [65]**	No	Use of evidence in decision models: an appraisal 1997.	Review	Not specific Economic decision models	Not specific	
**Mitton et al. 2007 [63]**	No	Knowledge Transfer and Exchange: Review and Synthesis of the Literature.	Systematic literature review(SLR)	Qualitative and quantitative studies	Scale based	Reporting quality only
**Vistad et al. 2006 [64]**	No	A critical review of patient-rated quality of life studies of long-term survivors of cervical cancer.	Systematic literature review(SLR)	RCT and Non-Randomized	Scale based	RoB and Relevancy
**Kmet et al. 2004 [66]**	No	Standard quality assessment criteria for evaluating primary research papers from a variety of fields.	Standardized Tool	Qualitative and quantitative studies Multiple designs simultaneously	Scale based	Reporting and RoB
**Thomas B. H. 2004** [67]	No	A process for systematically reviewing the literature: providing the research evidence for public health nursing interventions.	Technical Document (Recommendations) with a QA tool developed or added	Randomized and Non-randomized studies	Domain based (Ranking)	RoB only
**Slim et al. 2003 [68]**	No	Methodological index for non-randomized studies (MINORS): Development and validation of a new instrument.	Standardized Tool	Non randomized studies	Scale based	Reporting quality only
**Ryan et 2001 [69]**	No	Eliciting public preferences for healthcare: a systematic review of techniques.	Systematic literature review(SLR)	Not applicable	Not applicable	Reporting, RoB(Methodological) and Relevancy
**Lenert et al 2000 [70]**	No	Validity and Interpretation of Preference-Based Measures of Health-Related Quality of Life.	Technical Document (Recommendations)	Not applicable	Not applicable	RoB only
**Downs and Black. 1998 [71]**	No	The feasibility of creating a checklist for the assessment of the methodological quality both of randomized and non-randomized studies of health care interventions.	Standardized Tool	RCTs and Non RCTs	Scale based	Reporting, RoB(Methodological) and Relevancy
**Jadad et al. 1996 [72]**	No	Assessing the quality of reports of randomized clinical trials: Is blinding necessary?	Standardized Tool	RCTs	Scale based	RoB only
**Torrance et al. 1986 [73]**	No	Measurement of health state utilities for economic appraisal.	Technical Document (Recommendations)	Not applicable	Not applicable	Not applicable

* The year of first publication could not be ascertained. HSUV =Health states utility values; RoB = Risk of bias; QA=Quality appraisal, NICE= National Institute of Health and Care Excellence; PRO = Patient reported outcomes; HRQoL = Health related quality of life

Supplementary material 7: Frequency of occurrence of items in SLRs, checklists, tools and GPRs

Table A.10: Frequency of use of QA items in SLRs that appraised quality

Items	Absolute frequency of use in SLRs (based on NICE/ISPOR tools)	Absolute frequency of use in SLRs (Others)	Total frequency of use	% use (N=38)
Response rates++	14	13	27	68%
Statistical Analysis/ Data Analysis*	5	17	22	55%
Loss of follow up (Attrition or withdrawals)++	13	8	21	53%
Sample size++	12	9	21	53%
Respondent selection and recruitment (Sample selection bias) ++	8	12	20	50%
Missing (incomplete) data++	12	7	19	48%
Confounding/Baseline equivalency of groups*	0	17	17	43%
Appropriate health state description++	6	5	11	28%
Appropriate use of generic preference-based method++	8	3	11	28%
Inclusion and exclusion criteria++	8	3	11	28%
Other sources of bias++	7	4	11	28%
Appropriateness of study population++	4	6	10	25%
Blinding*	1	8	9	23%
Appropriate use of valuation method++	6	2	8	20%
Discussions, limitations and conclusions*	1	7	8	20%
Population the HSUVs collected from++	5	3	8	20%
Population used to value the health states within the measure++	4	4	8	20%
Reporting of results*	1	7	8	20%
Technique used to value the health states++	5	3	8	20%
Appropriateness of study country,(setting or facilities)	2	5	7	18%
Assessment of outcome	0	7	7	18%
Control and contemporary groups	0	7	7	18%
Study aims, objectives and rationale (Research question)	0	7	7	18%
Study design features /Experimental design*	0	7	7	18%
Follow up period	1	4	5	13%
Precision of estimates	3	2	5	13%
Source of tariff (value set) *	1	4	5	13%
Ascertainment of exposure	0	4	4	10%
Data collection methods/Measurement instrument	0	4	4	10%
Data sources/Source of HSUVs	1	3	4	10%
Randomization*	1	3	4	10%

Items	Absolute frequency of use in SLRs (based on NICE/ISPOR tools)	Absolute frequency of use in SLRs (Others)	Total frequency of use	% use (N=38)
Reporting bias	0	4	4	10%
Absence of outcome of interest at index date	0	3	3	8%
Ascertainment of cases and controls	0	3	3	8%
Case definition	0	3	3	8%
Selection of controls	0	3	3	8%
Selection of non-exposed cohorts	0	3	3	8%
Adequacy of follow up of cohorts	0	2	2	5%
Adherence to a predefined study protocol*	1	1	2	5%
Bibliographic details, including year of publication	0	2	2	5%
Indifference search procedure	1	1	2	5%
Measure used to describe the HSUVs++	1	1	2	5%
Non-normality of the distribution of utility values	1	1	2	5%
Representative of exposed cohorts	0	2	2	5%
Representativeness of cases	0	2	2	5%
Sources of funding*	1	1	2	5%
Administration procedure*	0	1	1	3%
Appropriateness of endpoints	0	1	1	3%
Attributes and levels	0	1	1	3%
Availability and accessibility of a study protocol	0	1	1	3%
Credible extrapolation of health state valuations?	0	1	1	3%
Description of basic data	0	1	1	3%
Ethical approval	0	1	1	3%
Generalizability of findings	1	0	1	3%
Important adverse events	0	1	1	3%
Instrument acceptability (level of detail, comprehensibility vs burden vs cognitive overload)	0	1	1	3%
Instrument reliability	0	1	1	3%
Instrument responsiveness	0	1	1	3%
Instrument validity (Face, Content and Construct)	0	1	1	3%
Instrument version	0	1	1	3%
Internal consistency of results	0	1	1	3%
Intervention measurement	0	1	1	3%
Literature Review:	0	1	1	3%
P value reporting	0	1	1	3%
Piloting and pretesting of the study	0	1	1	3%
Proxy rates by mRS level?	0	1	1	3%

Items	Absolute frequency of use in SLRs (based on NICE/ISPOR tools)	Absolute frequency of use in SLRs (Others)	Total frequency of use	% use (N=38)
Question framing	0	1	1	3%
Scoring algorithm*	0	1	1	3%
Sensitivity analysis	0	1	1	3%
Tests for internal validity	0	1	1	3%
Time of assessment	0	1	1	3%
Age of onset and duration in the health state	0	0	0	0%
Allocation sequence concealment	0	0	0	0%
Appropriateness of instrument	0	0	0	0%
Data sets	0	0	0	0%
Description and use of anchor states	0	0	0	0%
Homogeneity of sample	0	0	0	0%
Indirectness	0	0	0	0%
Instrument feasibility	0	0	0	0%
Integrity of intervention	0	0	0	0%
Interventions, control and contemporary groups	0	0	0	0%
Misclassification bias	0	0	0	0%
Model Selection and Performance	0	0	0	0%
Modeling techniques	0	0	0	0%

++ ISPOR Items (as recommended by ISPOR NICE and related publications)

+ Additional items (as informed by literature, theoretical considerations and internal expertise)

Source: Author's elaborations

Table A.11: Frequency of occurrence of QA items in checklist, tools and GPRs

Items	Absolute frequency of use in SLRs (NICE/ISPOR tools') [n=6]	Absolute frequency of use in SLRs (Other checklists, tools and GPRs)[n=27]	Total	% use (N=30)
Statistical Analysis/ Data Analysis*	3	20	23	66%
Confounding/Baseline equivalency of groups*	1	19	20	57%
Respondent selection and recruitment (Sample selection bias)++	2	15	17	49%
Blinding*	0	14	14	40%
Missing (incomplete) data++	3	10	13	37%
Response rates++	3	10	13	37%
Sample size++	3	9	12	34%
Study design features /Experimental design*	0	11	11	31%
Appropriateness of study population++	3	6	9	26%
Loss of follow up (Attrition or withdrawals) ++	3	6	9	26%
Study aims, objectives and rationale (Research question)	0	9	9	26%
Assessment of outcome	0	8	8	23%
Instrument validity (Face, Content and Construct)	1	6	7	20%
Adherence to a predefined study protocol*	0	6	6	17%
Appropriate health state description++	2	4	6	17%
Ascertainment of exposure	0	6	6	17%
Control and contemporary groups	0	6	6	17%
Discussions, limitations and conclusions	0	6	6	17%
Inclusion and exclusion criteria++	2	4	6	17%
Randomization*	0	6	6	17%
Reporting bias	0	6	6	17%
Reporting of results*	0	6	6	17%
Technique used to value the health states++	3	3	6	17%
Population used to value the health states within the measure++	4	1	5	14%
Follow up period	0	4	4	11%
Instrument acceptability (level of detail, comprehensibility vs burden vs cognitive overload)	0	4	4	11%
Instrument reliability	0	4	4	11%
Internal consistency of results	0	4	4	11%
Precision of estimates	1	3	4	11%
Appropriate use of generic preference-based method++	2	1	3	9%
Appropriateness of study country,(setting or facilities)	0	3	3	9%

Items	Absolute frequency of use in SLRs (NICE/ISPOR tools') [n=6]	Absolute frequency of use in SLRs (Other checklists, tools and GPRs)[n=27]	Total	% use (N=30)
Data collection methods/Measurement instrument	0	3	3	9%
Measure used to describe the HSUVs[++]	2	1	3	9%
Other sources of bias[++]	2	1	3	9%
Question framing	0	3	3	9%
Time of assessment	2	1	3	9%
Appropriate use of valuation method	1	1	2	6%
Appropriateness of endpoints	0	2	2	6%
Availability and accessibility of a study protocol	0	2	2	6%
Description and use of anchor states	0	2	2	6%
Important adverse events	0	2	2	6%
Interventions ,control and contemporary groups	0	2	2	6%
Misclassification bias	0	2	2	6%
Piloting and pre testing of the study	0	2	2	6%
Population the HSUVs collected from[++]	2	0	2	6%
Questionnaire response time	0	2	2	6%
Source of utility values*	0	2	2	6%
Sources of funding*	0	2	2	6%
Absence of outcome of interest at index date	0	1	1	3%
Adequacy of follow up of cohorts	0	1	1	3%
Administration procedure*	0	1	1	3%
Age of onset and duration in the health state	0	1	1	3%
Allocation sequence concealment	0	1	1	3%
Appropriateness of instrument	0	1	1	3%
Ascertainment of cases and controls	0	1	1	3%
Attributes and levels	0	1	1	3%
Case definition	0	1	1	3%
Data sets	0	1	1	3%
Description of basic data	0	1	1	3%
Ethical approval	0	1	1	3%
Generalizability of findings	0	1	1	3%
Homogeneity of sample	0	1	1	3%
Indifference search procedure*	0	1	1	3%
Indirectness	0	1	1	3%
Instrument feasibility	0	1	1	3%
Instrument responsiveness	0	1	1	3%
Integrity of intervention	0	1	1	3%
Literature Review:	0	1	1	3%
Model Selection and Performance	0	1	1	3%
Modeling techniques	0	1	1	3%
Outcomes assessed	0	1	1	3%

Items	Absolute frequency of use in SLRs (NICE/ISPOR tools') [n=6]	Absolute frequency of use in SLRs (Other checklists, tools and GPRs)[n=27]	Total	% use (N=30)
P value reporting	0	1	1	3%
Perspective of evaluation (measuring own health or someone's health).	0	1	1	3%
Publication bias	0	1	1	3%
Question and methods sequencing	0	1	1	3%
Recruitment period	0	1	1	3%
Representative of exposed cohorts	0	1	1	3%
Representativeness of cases	0	1	1	3%
Reproducibility	0	1	1	3%
Selection of controls	0	1	1	3%
Selection of non-exposed cohorts	0	1	1	3%
Sensitivity of instrument	1	0	1	3%
Understanding of participants	0	1	1	3%
Bibliographic details, including year of publication	0	0	0	0%

++ ISPOR Items (as recommended by ISPOR NICE and related publications)

* Additional items (as informed by literature, theoretical considerations and internal expertise)

Source: Author's elaborations

References

1. Di Tanna GL, Urbich M, Wirtz HS, Potrata B, Heisen M, Bennison C, Brazier J, Globe G: **Health State Utilities of Patients with Heart Failure: A Systematic Literature Review**. *PharmacoEconomics* 2021, **39**(2):211-229.

2. Aceituno D, Pennington M, Iruretagoyena B, Prina AM, McCrone P: **Health State Utility Values in Schizophrenia: A Systematic Review and Meta-Analysis**. *Value in health : the journal of the International Society for Pharmacoeconomics and Outcomes Research* 2020, **23**(9):1256-1267.

3. Blom EF, Haaf KT, de Koning HJ: **Systematic Review and Meta-Analysis of Community- and Choice-Based Health State Utility Values for Lung Cancer**. *PharmacoEconomics* 2020, **38**(11):1187-1200.

4. Cooper JT, Lloyd A, Sanchez JJG, Sörstadius E, Briggs A, McFarlane P: **Health related quality of life utility weights for economic evaluation through different stages of chronic kidney disease: a systematic literature review**. *Health and Quality of Life Outcomes* 2020, **18**(1):310.

5. Golicki D, Jaśkowiak K, Wójcik A, Młyńczak K, Dobrowolska I, Gawrońska A, Basak G, Snarski E, Hołownia-Voloskova M, Jakubczyk M *et al*: **EQ-5D-Derived Health State Utility Values in Hematologic Malignancies: A Catalog of 796 Utilities Based on a Systematic Review**. *Value in health : the journal of the International Society for Pharmacoeconomics and Outcomes Research* 2020, **23**(7):953-968.

6. Petrou S, Krabuanrat N, Khan K: **Preference-Based Health-Related Quality of Life Outcomes Associated with Preterm Birth: A Systematic Review and Meta-analysis**. *PharmacoEconomics* 2020, **38**(4):357-373.

7. Saeed YA, Phoon A, Bielecki JM, Mitsakakis N, Bremner KE, Abrahamyan L, Pechlivanoglou P, Feld JJ, Krahn M, Wong WWL: **A Systematic Review and Meta-Analysis of Health Utilities in Patients With Chronic Hepatitis C**. *Value in health : the journal of the International Society for Pharmacoeconomics and Outcomes Research* 2020, **23**(1):127-137.

8. Szabo SM, Audhya IF, Malone DC, Feeny D, Gooch KL: **Characterizing health state utilities associated with Duchenne muscular dystrophy: a systematic review**. *Quality of life research : an international journal of quality of life aspects of treatment, care and rehabilitation* 2020, **29**(3):593-605.

9. Buchanan-Hughes AM, Buti M, Hanman K, Langford B, Wright M, Eddowes LA: **Health state utility values measured using the EuroQol 5-dimensions questionnaire in adults with chronic hepatitis C: a systematic literature review and meta-analysis**. *Quality of life research : an international journal of quality of life aspects of treatment, care and rehabilitation* 2019, **28**(2):297-319.

10. Li L, Severens JLH, Mandrik O: **Disutility associated with cancer screening programs: A systematic review**. *PloS one* 2019, **14**(7):e0220148.

11. Magnus A, Isaranuwatchai W, Mihalopoulos C, Brown V, Carter R: **A Systematic Review and Meta-Analysis of Prostate Cancer Utility Values of Patients and Partners Between 2007 and 2016**. *MDM policy & practice* 2019, **4**(1):2381468319852332.

12. Paracha N, Abdulla A, MacGilchrist KS: **Systematic review of health state utility values in metastatic non-small cell lung cancer with a focus on previously treated patients**. *Health and Quality of Life Outcomes* 2018, **16**(1):179.

13. Meregaglia M, Cairns J: **A systematic literature review of health state utility values in head and neck cancer**. *Health and Quality of Life Outcomes* 2017, **15**(1):174.

14. Kua WS, Davis S: **PRS49 - Systematic Review of Health State Utilities in Children with Asthma**. *Value in Health* 2016, **19**(7):A557.

15. Paracha N, Thuresson PO, Moreno SG, MacGilchrist KS: **Health state utility values in locally advanced and metastatic breast cancer by treatment line: a systematic review**. *Expert review of pharmacoeconomics & outcomes research* 2016, **16**(5):549-559.

16. Carter GC, King DT, Hess LM, Mitchell SA, Taipale KL, Kiiskinen U, Rajan N, Novick D, Liepa AM: **Health state utility values associated with advanced gastric, oesophageal, or gastro-oesophageal junction adenocarcinoma: a systematic review**. *Journal of medical economics* 2015, **18**(11):954-966.

17. Afshari S, Ameri H, Daroudi RA, Shiravani M, Karami H, Akbari Sari A: **Health related quality of life in adults with asthma: a systematic review to identify the values of EQ-5D-5L instrument**. *J Asthma* 2021:1-10.

18. Carrello J, Hayes A, Killedar A, Von Huben A, Baur LA, Petrou S, Lung T: **Utility Decrements Associated with Adult Overweight and Obesity in Australia: A Systematic Review and Meta-Analysis**. *PharmacoEconomics* 2021, **39**(5):503-519.

19. Etxeandia-Ikobaltzeta I, Zhang Y, Brundisini F, Florez ID, Wiercioch W, Nieuwlaat R, Begum H, Cuello CA, Roldan Y, Chen R *et al*: **Patient values and preferences regarding VTE disease: a systematic review to inform American Society of Hematology guidelines**. *Blood advances* 2020, **4**(5):953-968.

20. Han R, François C, Toumi M: **Systematic Review of Health State Utility Values Used in European Pharmacoeconomic Evaluations for Chronic Hepatitis C: Impact on Cost-Effectiveness Results**. *Applied health economics and health policy* 2021, **19**(1):29-44.

21. Haridoss M, Bagepally BS, Natarajan M: **Health-related quality of life in rheumatoid arthritis: Systematic review and meta-analysis of EuroQoL (EQ-5D) utility scores from Asia**. *Int J Rheum Dis* 2021, **24**(3):314-326.

22. Jiang M, Ma Y, Li M, Meng R, Ma A, Chen P: **A comparison of self-reported and proxy-reported health utilities in children: a systematic review and meta-analysis**. *Health and Quality of Life Outcomes* 2021, **19**(1):45.

23. Park HY, Cheon HB, Choi SH, Kwon JW: **Health-Related Quality of Life Based on EQ-5D Utility Score in Patients With Tuberculosis: A Systematic Review**. *Front Pharmacol* 2021, **12**:659675.

24. Landeiro F, Mughal S, Walsh K, Nye E, Morton J, Williams H, Ghinai I, Castro Y, Leal J, Roberts N *et al*: **Health-related quality of life in people with predementia Alzheimer's disease, mild cognitive impairment or dementia measured with preference-based instruments: a systematic literature review**. *Alzheimers Res Ther* 2020, **12**(1):154.

25. Rebchuk AD, O'Neill ZR, Szefer EK, Hill MD, Field TS: **Health Utility Weighting of the Modified Rankin Scale: A Systematic Review and Meta-analysis**. *JAMA Network Open* 2020, **3**(4):e203767.

26. Yang Z, Li S, Wang X, Chen G: **Health state utility values derived from EQ-5D in psoriatic patients: a systematic review and meta-analysis**. *The Journal of dermatological treatment* 2020:1-8.

27. Yuan Y, Xiao Y, Chen X, Li J, Shen M: **A Systematic Review and Meta-Analysis of Health Utility Estimates in Chronic Spontaneous Urticaria**. *Front Med (Lausanne)* 2020, **7**:543290.

28. Foster E, Chen Z, Ofori-Asenso R, Norman R, Carney P, O'Brien TJ, Kwan P, Liew D, Ademi Z: **Comparisons of direct and indirect utilities in adult epilepsy populations: A systematic review**. *Epilepsia* 2019, **60**(12):2466-2476.

29. Hatswell AJ, Burns D, Baio G, Wadelin F: **Frequentist and Bayesian meta-regression of health state utilities for multiple myeloma incorporating systematic review and analysis of individual patient data**. *Health economics* 2019, **28**(5):653-665.

30. Khadka J, Kwon J, Petrou S, Lancsar E, Ratcliffe J: **Mind the (inter-rater) gap. An investigation of self-reported versus proxy-reported assessments in the derivation of childhood utility values for economic evaluation: A systematic review**. *Social science & medicine (1982)* 2019, **240**:112543.

31. Van Wilder L, Rammant E, Clays E, Devleesschauwer B, Pauwels N, De Smedt D: **A comprehensive catalogue of EQ-5D scores in chronic disease: results of a systematic review**. *Quality of life research : an international journal of quality of life aspects of treatment, care and rehabilitation* 2019, **28**(12):3153-3161.

32. Kwon J, Kim SW, Ungar WJ, Tsiplova K, Madan J, Petrou S: **A Systematic Review and Meta-analysis of Childhood Health Utilities**. *Medical decision making : an international journal of the Society for Medical Decision Making* 2018, **38**(3):277-305.

33. Tran AD, Fogarty G, Nowak AK, Espinoza D, Rowbotham N, Stockler MR, Morton RL: **A systematic review and meta-analysis of utility estimates in melanoma**. *The British journal of dermatology* 2018, **178**(2):384-393.

34. Ó Céilleachair A, O'Mahony JF, O'Connor M, O'Leary J, Normand C, Martin C, Sharp L: **Health-related quality of life as measured by the EQ-5D in the prevention, screening and management of cervical disease: A systematic review**. *Quality of life research : an international journal of quality of life aspects of treatment, care and rehabilitation* 2017, **26**(11):2885-2897.

35. Ward Fuller G, Hernandez M, Pallot D, Lecky F, Stevenson M, Gabbe B: **Health State Preference Weights for the Glasgow Outcome Scale Following Traumatic Brain Injury: A Systematic Review and Mapping Study**. *Value in health : the journal of the International Society for Pharmacoeconomics and Outcomes Research* 2017, **20**(1):141-151.

36. Zrubka Z, Rencz F, Závada J, Golicki D, Rupel VP, Simon J, Brodszky V, Baji P, Petrova G, Rotar A *et al*: **EQ-5D studies in musculoskeletal and connective tissue diseases in eight Central and Eastern European countries: a systematic literature review and meta-analysis**. *Rheumatol Int* 2017, **37**(12):1957-1977.

37. Brennan VK, Mauskopf J, Colosia AD, Copley-Merriman C, Hass B, Palencia R: **Utility estimates for patients with Type 2 diabetes mellitus after experiencing a myocardial infarction or stroke: a systematic review**. *Expert review of pharmacoeconomics & outcomes research* 2015, **15**(1):111-123.

38. Eiring Ø, Landmark BF, Aas E, Salkeld G, Nylenna M, Nytrøen K: **What matters to patients? A systematic review of preferences for medication-associated outcomes in mental disorders**. *BMJ open* 2015, **5**(4):e007848.

39. Gheorghe A, Moran G, Duffy H, Roberts T, Pinkney T, Calvert M: **Health Utility Values Associated with Surgical Site Infection: A Systematic Review**. *Value in health : the journal of the International Society for Pharmacoeconomics and Outcomes Research* 2015, **18**(8):1126-1137.

40. Li YK, Alolabi N, Kaur MN, Thoma A: **A systematic review of utilities in hand surgery literature**. *J Hand Surg Am* 2015, **40**(5):997-1005.

41. Brazier J, Ara R, Azzabi I, Busschbach J, Chevrou-Séverac H, Crawford B, Cruz L, Karnon J, Lloyd A, Paisley S *et al*: **Identification, Review, and Use of Health State Utilities in Cost-Effectiveness Models: An ISPOR Good Practices for Outcomes Research Task Force Report**. *Value in health : the journal of the International Society for Pharmacoeconomics and Outcomes Research* 2019, **22**(3):267-275.

42. Ara R, Brazier J, Peasgood T, Paisley S: **The Identification, Review and Synthesis of Health State Utility Values from the Literature**. *PharmacoEconomics* 2017, **35**(Suppl 1):43-55.

43. Papaioannou D, Brazier J, Paisley S: **NICE Decision Support Unit Technical Support Documents**. In: *NICE DSU Technical Support Document 9: The Identification, Review and Synthesis of Health State Utility Values from the Literature.* edn. London: National Institute for Health and Care Excellence (NICE); 2010.

44. Papaioannou D, Brazier J, Paisley S: **Systematic searching and selection of health state utility values from the literature**. *Value in health : the journal of the International Society for Pharmacoeconomics and Outcomes Research* 2013, **16**(4):686-695.

45. Sterne JAC, Savović J, Page MJ, Elbers RG, Blencowe NS, Boutron I, Cates CJ, Cheng H-Y, Corbett MS, Eldridge SM *et al*: **RoB 2: a revised tool for assessing risk of bias in randomised trials**. *BMJ (Clinical research ed)* 2019, **366**:l4898.

46. Xie F, Pickard AS, Krabbe PF, Revicki D, Viney R, Devlin N, Feeny D: **A Checklist for Reporting Valuation Studies of Multi-Attribute Utility-Based Instruments (CREATE)**. *PharmacoEconomics* 2015, **33**(8):867-877.

47. Zhang Y, Alonso-Coello P, Guyatt GH, Yepes-Nuñez JJ, Akl EA, Hazlewood G, Pardo-Hernandez H, Etxeandia-Ikobaltzeta I, Qaseem A, Williams JW, Jr. *et al*: **GRADE Guidelines: 19. Assessing the certainty of evidence in the importance of outcomes or values and preferences-Risk of bias and indirectness**. *J Clin Epidemiol* 2019, **111**:94-104.

48. Petrou S, Kwon J, Madan J: **A Practical Guide to Conducting a Systematic Review and Meta-analysis of Health State Utility Values**. *PharmacoEconomics* 2018, **36**(9):1043-1061.

49. Wailoo AJ, Hernandez-Alava M, Manca A, Mejia A, Ray J, Crawford B, Botteman M, Busschbach J: **Mapping to Estimate Health-State Utility from Non-Preference-Based Outcome Measures: An ISPOR Good Practices for Outcomes Research Task Force Report**. *Value in health : the journal of the International Society for Pharmacoeconomics and Outcomes Research* 2017, **20**(1):18-27.

50. Downes MJ, Brennan ML, Williams HC, Dean RS: **Development of a critical appraisal tool to assess the quality of cross-sectional studies (AXIS)**. *BMJ open* 2016, **6**(12):e011458.

51. Gupta A, Giambrone AE, Gialdini G, Finn C, Delgado D, Gutierrez J, Wright C, Beiser AS, Seshadri S, Pandya A *et al*: **Silent Brain Infarction and Risk of Future Stroke: A Systematic Review and Meta-Analysis**. *Stroke* 2016, **47**(3):719-725.

52. O'Connor SR, Tully MA, Ryan B, Bradley JM, Baxter GD, McDonough SM: **Failure of a numerical quality assessment scale to identify potential risk of bias in a systematic review: a comparison study**. *BMC Research Notes* 2015, **8**:224.

53. Sterne JAC, Hernán MA, Reeves BC, Savović J, Berkman ND, Viswanathan M, Henry D, Altman DG, Ansari MT, Boutron I *et al*: **ROBINS-I: a tool for assessing risk of bias in non-randomised studies of interventions**. *BMJ (Clinical research ed)* 2016, **355**:i4919.

54. Gupta A, Kesavabhotla K, Baradaran H, Kamel H, Pandya A, Giambrone AE, Wright D, Pain KJ, Mtui EE, Suri JS *et al*: **Plaque echolucency and stroke risk in asymptomatic carotid stenosis: a systematic review and meta-analysis**. *Stroke* 2015, **46**(1):91-97.

55. Wolowacz SE, Briggs A, Belozeroff V, Clarke P, Doward L, Goeree R, Lloyd A, Norman R: **Estimating Health-State Utility for Economic Models in Clinical Studies: An ISPOR Good Research Practices Task Force Report**. *Value in health : the journal of the International Society for Pharmacoeconomics and Outcomes Research* 2016, **19**(6):704-719.

56. Calvert M, Blazeby J, Altman DG, Revicki DA, Moher D, Brundage MD: **Reporting of patient-reported outcomes in randomized trials: the CONSORT PRO extension**. *Jama* 2013, **309**(8):814-822.

57. Herzog R, Álvarez-Pasquin MJ, Díaz C, Del Barrio JL, Estrada JM, Gil Á: **Are healthcare workers' intentions to vaccinate related to their knowledge, beliefs and attitudes? a systematic review**. *BMC public health* 2013, **13**(1):154.

58. Brazier J, Rowen D: **NICE Decision Support Unit Technical Support Documents**. In: *NICE DSU Technical Support Document 11: Alternatives to EQ-5D for Generating Health State Utility Values.* edn.: National Institute for Health and Care Excellence (NICE)

2011.

59. Higgins JPT TJ, Chandler J, Cumpston M, Li T, Page MJ, Welch VA (editors): **Cochrane Handbook for Systematic Reviews of Interventions** In. Edited by VA W. Cochrane: Cochrane; 2021.

60. Park J, Lee Y, Seo H, Jang B, Son H, Kim S, Shin S: **Risk of Bias Assessment tool for Non-randomized Studies (RoBANS): Development and validation of a new instrument**. In: *19th Cochrane Colloquium: 19-22 Oct 2011; Madrid, Spain*: John Wiley & Sons; 2011.

61. Accent and RAND Europe: **Review of stated preference and willingness to pay methods.** . In. Accent and RAND Europe: Accent and RAND Europe; 2010: 110.

62. Arnold D, Girling A, Stevens A, Lilford R: **Comparison of direct and indirect methods of estimating health state utilities for resource allocation: review and empirical analysis**. *BMJ (Clinical research ed)* 2009, **339**:b2688.

63. Mitton C, Adair CE, McKenzie E, Patten SB, Waye Perry B: **Knowledge transfer and exchange: review and synthesis of the literature**. *Milbank Q* 2007, **85**(4):729-768.

64. Vistad I, Fosså SD, Dahl AA: **A critical review of patient-rated quality of life studies of long-term survivors of cervical cancer**. *Gynecologic oncology* 2006, **102**(3):563-572.

65. Cooper N, Coyle D, Abrams K, Mugford M, Sutton A: **Use of evidence in decision models: an appraisal of health technology assessments in the UK since 1997**. *J Health Serv Res Policy* 2005, **10**(4):245-250.

66. Kmet LMLRCCLSAHFfMRAHTAUUoCFoMCH: **Standard quality assessment criteria for evaluating primary research papers from a variety of fields**. Edmonton: Alberta Heritage Foundation for Medical Research; 2004.

67. Thomas BH, Ciliska D, Dobbins M, Micucci S: **A process for systematically reviewing the literature: providing the research evidence for public health nursing interventions**. *Worldviews Evid Based Nurs* 2004, **1**(3):176-184.

68. Slim K, Nini E, Forestier D, Kwiatkowski F, Panis Y, Chipponi J: **Methodological index for non-randomized studies (minors): development and validation of a new instrument**. *ANZ journal of surgery* 2003, **73**(9):712-716.

69. Ryan M, Scott DA, Reeves C, Bate A, van Teijlingen ER, Russell EM, Napper M, Robb CM: **Eliciting public preferences for healthcare: a systematic review of techniques**. *Health technology assessment (Winchester, England)* 2001, **5**(5):1-186.

70. Lenert L, Kaplan RM: **Validity and interpretation of preference-based measures of health-related quality of life**. *Medical care* 2000, **38**(9 Suppl):Ii138-150.

71. Downs SH, Black N: **The feasibility of creating a checklist for the assessment of the methodological quality both of randomised and non-randomised studies of health care interventions**. *Journal of epidemiology and community health* 1998, **52**(6):377-384.

72. Jadad AR, Moore RA, Carroll D, Jenkinson C, Reynolds DJ, Gavaghan DJ, McQuay HJ: **Assessing the quality of reports of randomized clinical trials: is blinding necessary?** *Controlled clinical trials* 1996, **17**(1):1-12.

73. Torrance GW: **Measurement of health state utilities for economic appraisal**. *J Health Econ* 1986, **5**(1):1-30.

74. Brockbank J, Krause T, Moss E, Pedersen AM, Mørup MF, Ahdesmäki O, Vaughan J, Brodtkorb TH: **Health state utility values in major depressive disorder treated with pharmacological interventions: a systematic literature review**. *Health and Quality of Life Outcomes* 2021, **19**(1):94.

75. Chataway J, Murphy N, Khurana V, Schofield H, Findlay J, Adlard N: **Secondary progressive multiple sclerosis: a systematic review of costs and health state utilities**. *Curr Med Res Opin* 2021:1-10.

76. Houten R, Fleeman N, Kotas E, Boland A, Lambe T, Duarte R: **A systematic review of health state utility values for thyroid cancer**. *Quality of life research : an international journal of quality of life aspects of treatment, care and rehabilitation* 2021, **30**(3):675-702.

77. Thorrington D, Eames K: **Measuring Health Utilities in Children and Adolescents: A Systematic Review of the Literature**. *PloS one* 2015, **10**(8):e0135672.

78. Betts MB, Rane P, Bergrath E, Chitnis M, Bhutani MK, Gulea C, Qian Y, Villa G: **Utility value estimates in cardiovascular disease and the effect of changing elicitation methods: a systematic literature review**. *Health and Quality of Life Outcomes* 2020, **18**(1):251.

79. Mohindru B, Turner D, Sach T, Bilton D, Carr S, Archangelidi O, Bhadhuri A, Whitty JA: **Health State Utility Data in Cystic Fibrosis: A Systematic Review**. *PharmacoEconomics - open* 2020, **4**(1):13-25.

80. Xia Q, Campbell JA, Ahmad H, Si L, de Graaff B, Otahal P, Palmer AJ: **Health state utilities for economic evaluation of bariatric surgery: A comprehensive systematic review and meta-analysis**. *Obes Rev* 2020, **21**(8):e13028.

81. Zhao T, Winzenberg T, de Graaff B, Aitken D, Ahmad H, Palmer AJ: **A systematic review and meta-analysis of health state utility values for osteoarthritis-related conditions**. *Arthritis care & research* 2020.

82. Prevolnik Rupel V, Divjak M, Zrubka Z, Rencz F, Gulácsi L, Golicki D, Mirowska-Guzel D, Simon J, Brodszky V, Baji P *et al*: **EQ-5D studies in nervous system diseases in eight Central and East European countries: a systematic literature review**. *The European journal of health economics : HEPAC : health economics in prevention and care* 2019, **20**(Suppl 1):109-117.

83. Shiri T, Khan K, Keaney K, Mukherjee G, McCarthy ND, Petrou S: **Pneumococcal Disease: A Systematic Review of Health Utilities, Resource Use, Costs, and Economic Evaluations of Interventions**. *Value in health : the journal of the International Society for Pharmacoeconomics and Outcomes Research* 2019, **22**(11):1329-1344.

84. Tonmukayakul U, Le LK, Mudiyanselage SB, Engel L, Bucholc J, Mulhern B, Carter R, Mihalopoulos C: **A systematic review of utility values in children with cerebral palsy**. *Quality of life research : an international journal of quality of life aspects of treatment, care and rehabilitation* 2019, **28**(1):1-12.

85. Wittenberg E, James LP, Prosser LA: **Spillover Effects on Caregivers' and Family Members' Utility: A Systematic Review of the Literature**. *PharmacoEconomics* 2019, **37**(4):475-499.

86. Yoon AY, Bozzuto L, Seto AJ, Fisher CS, Chatterjee A: **A Systematic Review of Utility Score Assessments in the Breast Surgery Cost-Analysis Literature**. *Annals of surgical oncology* 2019, **26**(5):1190-1201.

87. Zhou J, Millier A, François C, Aballéa S, Toumi M: **Systematic review of utility values used in the pharmacoeconomic evaluations for schizophrenia: implications on cost-effectiveness results**. *Journal of market access & health policy* 2019, **7**(1):1648973.

88. Batóg P, Rencz F, Péntek M, Gulácsi L, Filipiak KJ, Prevolnik Rupel V, Simon J, Brodszky V, Baji P, Závada J *et al*: **EQ-5D studies in cardiovascular diseases in eight**

Central and Eastern European countries: a systematic review of the literature. *Kardiol Pol* 2018, **76**(5):860-870.

89. Blieden Betts M, Gandra SR, Cheng LI, Szatkowski A, Toth PP: **Differences in utility elicitation methods in cardiovascular disease: a systematic review**. *Journal of medical economics* 2018, **21**(1):74-84.

90. Brown V, Tan EJ, Hayes AJ, Petrou S, Moodie ML: **Utility values for childhood obesity interventions: a systematic review and meta-analysis of the evidence for use in economic evaluation**. *Obes Rev* 2018, **19**(7):905-916.

91. Dossa F, Josse J, Acuna SA, Baxter NN: **Health State Utility Values for Ileostomies and Colostomies: a Systematic Review and Meta-Analysis**. *Journal of gastrointestinal surgery : official journal of the Society for Surgery of the Alimentary Tract* 2018, **22**(5):894-905.

92. Forsythe A, Brandt PS, Dolph M, Patel S, Rabe APJ, Tremblay G: **Systematic review of health state utility values for acute myeloid leukemia**. *ClinicoEconomics and Outcomes Research: CEOR* 2018, **10**:83-92.

93. Vo NX, Van Ha T, Chaikledkaew U: **The quality of life - A systematic review orientation to establish utility score in Vietnam**. *Systematic Reviews in Pharmacy* 2017, **8**(1):92-96.

94. Blanchard P, Volk RJ, Ringash J, Peterson SK, Hutcheson KA, Frank SJ: **Assessing head and neck cancer patient preferences and expectations: A systematic review**. *Oral Oncol* 2016, **62**:44-53.

95. Herdman M, Cole A, Hoyle CK, Coles V, Carroll S, Devlin N: **Sources and Characteristics of Utility Weights for Economic Evaluation of Pediatric Vaccines: A Systematic Review**. *Value in health : the journal of the International Society for Pharmacoeconomics and Outcomes Research* 2016, **19**(2):255-266.

96. Jeong K, Cairns J: **Systematic review of health state utility values for economic evaluation of colorectal cancer**. *Health Economics Review* 2016, **6**(1):36.

97. Malinowski KP, Kawalec P: **Health utility of patients with Crohn's disease and ulcerative colitis: a systematic review and meta-analysis**. *Expert review of pharmacoeconomics & outcomes research* 2016, **16**(4):441-453.

98. Moayeri F, Hsueh YS, Clarke P, Hua X, Dunt D: **Health State Utility Value in Chronic Obstructive Pulmonary Disease (COPD); The Challenge of Heterogeneity: A Systematic Review and Meta-Analysis**. *Copd* 2016, **13**(3):380-398.

99. Smith-Palmer J, Bae JP, Boye KS, Norrbacka K, Hunt B, Valentine WJ: **Evaluating health-related quality of life in type 1 diabetes: a systematic literature review of utilities for adults with type 1 diabetes**. *ClinicoEconomics and Outcomes Research: CEOR* 2016, **8**:559-571.

100. Stevanović J, Pechlivanoglou P, Kampinga MA, Krabbe PF, Postma MJ: **Multivariate Meta-Analysis of Preference-Based Quality of Life Values in Coronary Heart Disease**. *PloS one* 2016, **11**(3):e0152030.

101. Møller AH, Erntoft S, Vinding GR, Jemec GB: **A systematic literature review to compare quality of life in psoriasis with other chronic diseases using EQ-5D-derived utility values**. *Patient Related Outcome Measures* 2015, **6**:167-177.

102. Tran BX, Nguyen LH, Ohinmaa A, Maher RM, Nong VM, Latkin CA: **Longitudinal and cross sectional assessments of health utility in adults with HIV/AIDS: a systematic review and meta-analysis**. *BMC health services research* 2015, **15**:7.

103. Zhang Y, Coello PA, Guyatt GH, Yepes-Nuñez JJ, Akl EA, Hazlewood G, Pardo-Hernandez H, Etxeandia-Ikobaltzeta I, Qaseem A, Williams JW, Jr. *et al*: **GRADE guidelines: 20. Assessing the certainty of evidence in the importance of outcomes or values and preferences-inconsistency, imprecision, and other domains**. *J Clin Epidemiol* 2019, **111**:83-93.

104. Xie F, Zoratti M, Chan K, Husereau D, Krahn M, Levine O, Clifford T, Schunemann H, Guyatt G: **Toward a Centralized, Systematic Approach to the Identification, Appraisal, and Use of Health State Utility Values for Reimbursement Decision Making: Introducing the Health Utility Book (HUB)**. *Medical decision making : an international journal of the Society for Medical Decision Making* 2019, **39**(4):370-378.

105. O'Connor M, Gallagher P, Waller J, Martin CM, O'Leary JJ, Sharp L: **Adverse psychological outcomes following colposcopy and related procedures: a systematic review**. *BJOG : an international journal of obstetrics and gynaecology* 2016, **123**(1):24-38.

106. Higgins JPT, Altman DG, Gøtzsche PC, Jüni P, Moher D, Oxman AD, Savović J, Schulz KF, Weeks L, Sterne JAC: **The Cochrane Collaboration's tool for assessing risk of bias in randomised trials**. *BMJ (Clinical research ed)* 2011, **343**:d5928.
